# Potential Therapeutic Targets for Neuroblastoma Screened through Mendelian Randomization Analysis

**DOI:** 10.34172/aim.35114

**Published:** 2025-11-01

**Authors:** Zhenge Yang, Yunlong Zhang, Shan Wang

**Affiliations:** ^1^Department of Pediatric Surgical Oncology, Children’s Hospital of Chongqing Medical University; National Clinical Research Center for Children and Adolescents’ Health and Diseases; Ministry of Education Key Laboratory of Child Development and Disorders, Chongqing, China.; ^2^Children’s Urogenital Development and Tissue Engineering of Chongqing Education Commission of China, Chongqing, China

**Keywords:** Mendelian randomization (MR), Neuroblastoma (NB), Therapeutic targets

## Abstract

**Background:**

Neuroblastoma (NB) is the most prevalent extracranial solid tumor in children. Therefore, urgent exploration of novel therapeutic targets and more effective approaches is imperative to enhance the prognosis of these children.

**Methods:**

NB datasets were obtained from OpenGWAS—ieu-a-816 (1,627 cases; 3,254 controls; European ancestry) and prot-a-2003 (N=3,301; European ancestry)—and were combined by inverse-variance meta-analysis. Adrenal expression quantitative trait loci (eQTL) data were sourced from GTEx v8 (n=233). Bidirectional Mendelian randomization (MR) and summary-based MR analyses were conducted to infer the causal relationship between adrenal-related genes and NB, with validation by Steiger directionality testing and phenotype scanning. Blood cis-eQTL datasets were obtained from eQTLGen (n=31,684 across 37 cohorts), and immune-cell data were retrieved from OpenGWAS (731 traits; IDs ebi-a-GCST90001391–GCST90002121). Using immune-cell traits as intermediate variables, bidirectional MR assessed the causal relationship between blood-related genes and NB, and colocalization analysis was performed for blood-related genes and immune cells.

**Results:**

Using two-sample bidirectional MR, we identified eight adrenal genes associated with NB risk. Upregulated expression of CPNE1 (OR 0.94, 95% CI [0.93–0.95]; *P*=1.69×10^-31^), ZNF559 (OR 0.93, 95% CI [0.92–0.95]; *P*=5.96×10^-11^), TTC18 (OR 0.86, 95% CI [0.85–0.87]; *P*=1.04×10^-110^), DNAJC9 (OR 0.85, 95% CI [0.84–0.86]; *P*=1.62×10^-109^), and EP400NL (OR 0.84, 95% CI [0.83–0.84]; *P*<1×10^-300^) was associated with lower NB risk, whereas upregulation of BTNL2 (OR 1.07, 95% CI [1.07–1.07]; *P*<1×10^-300^), FAM182B (OR 1.07, 95% CI [1.06–1.08]; *P*=3.94×10^-23^), and NBPF3 (OR 1.13, 95% CI [1.12–1.13]; *P*=6.49×10^-5^) was associated with higher risk. In the blood, 43 genes influence NB by affecting the expression of 54 immune cell phenotypes. Among these genes and immune cells, eight exhibited colocalization effects with seven immune cells, indicating their potential as therapeutic targets.

**Conclusion:**

This study revealed that certain genes in the adrenal glands and blood affect the occurrence of NB, with immune cells playing a crucial role in the process influenced by blood genes. MR and colocalization prioritize candidate genes/putative therapeutic targets for NB.

## Introduction

 Neuroblastoma (NB) is the most common extracranial malignant solid tumor in childhood, accounting for approximately 8%–10% of childhood cancer incidence with a high mortality rate of up to 15%.^1,2^ NB exhibits significant heterogeneity, resulting in unique and variable biological and clinical features. Tumors may undergo spontaneous regression or widespread metastasis.^3^

 Currently, risk stratification in NB is based on tumor staging, age at initial diagnosis, histopathological classification, and molecular characteristics (chromosomal ploidy, *N- Myc* gene amplification, 11q abnormalities), guiding clinical management.^4^ Children with low- or intermediate-risk NB can achieve cure through surgical resection alone or in combination with low-intensity chemotherapy.^5^ However, high-risk patients with NB, even when subjected to multimodal and high-intensity combination therapies (induction chemotherapy, surgery, autologous stem cell transplantation, radiotherapy, anti-GD2 monoclonal antibody therapy, and maintenance therapy with retinoic acid),^6-9^ still face a 5-year survival rate of approximately 50%.^10^ Besides the aforementioned methods, new approaches have emerged in clinical trials in the field of tumor treatment. For instance, antibody-drug conjugates (ADCs) are arguably the most successful conjugate drug representatives. Similar conjugate drugs to ADCs include radiolabeled drug conjugates, small molecule drug conjugates, antibody immune stimulation conjugates, peptide drug conjugates, antibody fragment drug conjugates, antibody-cell conjugates, virus-like drug conjugates, antibody oligonucleotide conjugates, and antibody biopolymer conjugates. Although the efficacy of conjugated receptors has been validated in the treatment of tumors, such as lung and breast cancers,^11,12^ no clinical trial results are currently available for NB.^13^ The treatment of such patients has reached a bottleneck, necessitating urgent exploration of new therapeutic targets and more effective therapeutic approaches to further enhance the survival outcomes for these children.

 NB most commonly occurs in the adrenal glands and sympathetic ganglia and is believed to originate from neural crest-derived sympathoadrenal progenitor cells and developing adrenal chromaffin cells.^14,15^ Because NB arises from the sympathoadrenal lineage (including adrenal medulla), we pre-specified adrenal-related eQTL resources for instrument construction. We additionally evaluated blood/immune eQTLs to explore neuro-immune influences and leverage well-powered reference panels; these choices were defined *a priori*. Recently, increasing research has supported an association between NB and adrenal glands.^16-19^ However, the correlation between NB and the adrenal glands is well-established, and the causative relationship between adrenal-related gene expression and the occurrence of NB is yet to be clarified. As immunotherapy demonstrates significant efficacy in hematologic malignancies and solid tumors, including lung cancer and melanoma, it provides a new direction for the treatment of high-risk NB in children. Immunotherapy, particularly GD-2 antibodies in combination with other drugs, has become a crucial component in treating high-risk NB.^20,21^ However, NB possesses various immune evasion mechanisms, including the downregulation of major histocompatibility complex (MHC)-I molecules, affecting T cell-mediated cytotoxicity in high-risk NB and inhibiting NK cell function in patients with NB. The convergence of multiple mechanisms contributes to NB’s ability to evade immune surveillance and destruction, necessitating continuous exploration of immunotherapy applications in NB.

 Recently, Mendelian randomization (MR) analysis has been widely applied to discover potential drug targets.^22^ MR is a genetic instrumental variable analysis that typically utilizes single nucleotide polymorphisms (SNPs) from genome-wide association studies (GWAS) as genetic instruments to determine the causal impact of an exposure on an outcome. Unlike observational studies, MR can avoid the influence of confounding factors. Advances in high-throughput genomic and proteomic techniques in the plasma have facilitated the identification of potential therapeutic targets for various diseases, including Crohn’s disease and multiple sclerosis.^23,24^ Mendelian randomization (MR) uses germline variants as instruments for gene expression to obtain evidence consistent with causality, thereby reducing confounding and reverse causation. Our two-sample cis-eQTL MR relies on three assumptions: relevance (the instruments are associated with the exposure—gene expression), independence (instruments are not associated with confounders, supported by random allocation and ancestry control), and exclusion restriction (instruments affect NB risk only through the exposure). We addressed these by stringent cis-eQTL selection and LD clumping (relevance), cohort ancestry control (independence), and colocalization/HEIDI plus Steiger directionality and, where multi-SNP instruments exist, pleiotropy diagnostics (exclusion restriction). Findings are therefore presented as hypothesis-generating, consistent with MR’s evidential scope. However, a limited number of reported MR studies are currently integrating GWAS and expression quantitative trait loci (eQTL) data. Genome-wide association studies have identified germline susceptibility signals for NB, yet it remains unknown which genes’ cis-regulated expression causally contributes to risk and in which tissues. Prior genetics/MR analyses have largely relied on candidate-gene or annotation-based approaches, often without tissue-appropriate instruments, colocalization, or directionality tests, so causal mediation has not been clarified. We therefore conducted a two-sample cis-eQTL MR prioritising tissues relevant to NB biology (sympathoadrenal/adrenal) and leveraging blood/immune resources for complementary evidence, and we complemented MR with colocalization/HEIDI and Steiger. Results are presented as hypothesis-generating candidates for subsequent functional validation. Consequently, this study aimed to analyze the correlation between adrenal and blood genes, immune cells, and the occurrence of NB using MR methods to identify potential therapeutic targets and provide new insights for treating high-risk NB groups.

## Materials and Methods

###  Study Design and Data Resources

**Figure 1:**
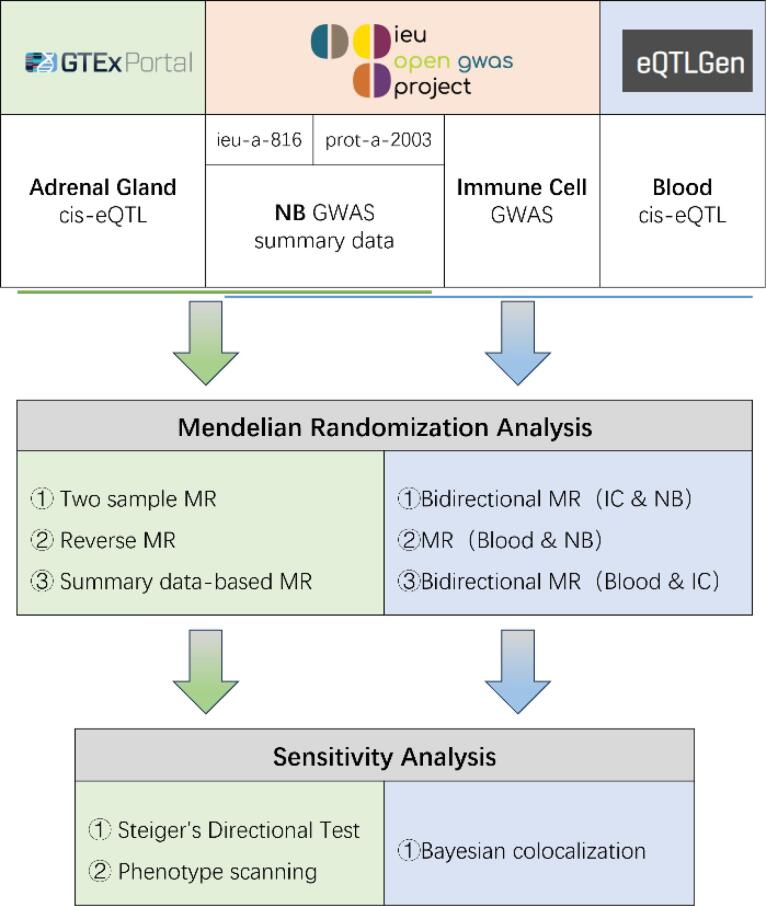
Study Design for Identification of Adrenal Gland Genes, Immune Cells, And Blood Genes causally associated with NB

 The study design is illustrated in Figure 1. NB GWAS data were obtained from the OpenGWAS website (https://gwas.mrcieu.ac.uk), including two datasets: ieu-a-816 (ncase: 1,627; ncontrol: 3,254)^25^ and prot-a-2003 (sample size: 3,301),^26^ both of European ancestry. The two GWAS summary datasets were merged using the Meta software. Exposure (cis-eQTL) and outcome (NB GWAS) datasets were sourced from distinct cohorts based on public metadata; no intentional overlap was present. The two NB GWAS (ieu-a-816; prot-a-2003) were treated as independent and combined by inverse-variance meta-analysis. While residual overlap cannot be entirely excluded, bias is expected to be minimal given strong instruments. The adrenal eQTL data were sourced from GTExPortal (version 8, sample size: 233, https://gtexportal.org).^27^ Bidirectional and summary-based MR analyses were conducted to infer the causal relationship between adrenal-related genes and NB. The results were validated using Steiger directionality testing and phenotype scanning. The blood eQTL dataset, downloaded from https://www.eqtlgen.org/, encompasses genetic data for blood gene expression from 31,684 individuals across 37 datasets.^28^ Immune cell GWAS data were obtained from OpenGWAS (https://gwas.mrcieu.ac.uk/), encompassing 731 types of immune cell data with IDs ranging from ebi-a-GCST90001391 to ebi-a-GCST90002121.^29^ Utilizing immune cells as intermediate variables, bidirectional MR analysis was employed to assess the causal relationship between blood-related genes and NB. Colocalization analysis was performed on the blood-related genes and immune cells. Unless otherwise specified, MR slopes are estimated by IVW (multiplicative random-effects) and plotted on the log(OR) scale; for single-SNP instruments, we used the Wald ratio. SMR panels plot SMR slopes on the log(OR) scale. Figure captions state axis scale and metrics (BH-FDR q, HEIDI P, PPH4). This study follows the STROBE-MR and STREGA reporting guidelines; we explicitly state MR assumptions, data sources with sample sizes/ancestry, instrument selection and strength, applicability of sensitivity analyses, multiple-testing control, and generalizability. Completed checklists are provided in Supplementary File, Table S1, S2.

## MR Analyses and Statistics

###  Methods Utilized for the Adrenal Section

####  MR Analysis of Adrenal eQTL and NB

 In this study, we utilized adrenal eQTL as the exposure, and the data selection criteria were as follows: 1) SNPs depicting genome-wide significant correlation (*P*< 5e‒8); 2) exclusion of SNPs located in the MHC region (chr6, 26–34 Mb; chr8, 7.2–12.5 Mb); 3) linkage disequilibrium (LD) with r^2^ < 0.001. After screening, 58,552 SNPs and 3,103 genes were obtained. Using all available NB GWAS summary data as the outcome, we conducted a forward MR analysis using the R package “TwoSampleMR (version 0.5.7)”. If two or more SNPs were available for a gene, the inverse variance-weighted MR (MR-IVW) method was applied. The ‘Wald ratio’ method was used to assess whether only one SNP was available. After calculating the *P*-values, a Bonferroni correction was employed to adjust for multiple testing.

####  Reverse MR Analysis of Adrenal eQTL and NB

 Applying the exposure criteria from the NB GWAS summary (screening threshold:* P* < 0.05), we conducted a reverse MR analysis with all adrenal gland eQTL data as the outcomes. The effect was estimated using the MR-IVW, MR-Egger, weighted median, simple mode, and weighted mode.

####  SMR

 SMR analysis was conducted using adrenal gland eQTL as the exposure and NB as the outcome, requiring a significance level of *P*< 5e‒8. The *P*-values of the SMR results were corrected using the Benjamini-Hochberg method.

###  Steiger’s Directional Test and Phenotype Scanning

 We performed Steiger filtering to ensure a directional association between adrenal eQTL and NB. The results were considered statistically significant at *P*< 0.05. Additionally, phenotype scanning was performed by searching previous GWAS data to uncover the associations of identified pQTLs with other traits. Phenotype scanning utilized both the ‘phenoscanner’ tools. A pleiotropic SNP was considered when meeting the following criteria: 1) the association reached genome-wide significance (*P* < 5 × 10^−8^); 2) the GWAS was conducted in a population of European ancestry; 3) the SNPs were associated with any known risk factors of NB, including metabolic traits, proteins, or clinical traits.

###  Methods Utilized for the Blood and IC Section

####  MR Analysis of Immune Cells and NB

 MR analysis was conducted with each immune cell as an exposure (following the same screening criteria mentioned earlier), separately assessing its association with NB as an outcome. The analysis employed MR-IVW for evaluation, and the resulting *P* values were corrected using Bonferroni’s method. This corresponds to Step 1 in the workflow diagram.

####  Reverse MR Analysis of Immune Cells and NB

 Using NB as the exposure, with screening criteria similar to those mentioned earlier, reverse MR analyses were conducted separately for each immune cell as the outcome. The assessment involved the MR-IVW, MR-Egger, weighted median, simple mode, and weighted mode methods. This corresponds to Step 2 of the workflow diagram.

####  MR Analysis of Blood eQTLs and NB

 Using blood eQTLs as exposure, we applied screening criteria similar to the previous section, and conducted MR analysis for each gene with NB as the outcome. The evaluation was performed using the MR-IVW, MR-Egger, weighted median, simple mode, and weighted mode methods. *P* values were not corrected. This corresponds to Step 3 in the workflow diagram.

####  MR Analysis of Blood eQTLs and Immune Cells

 Using blood eQTLs as exposure, we applied screening criteria similar to the previous section, and conducted MR analysis for each gene with each immune cell as the outcome. If two or more SNPs were available for a gene, evaluation was performed using the MR-IVW method. The Wald ratio method was used to assess whether only one SNP was available. The resulting *P* values were corrected using Bonferroni’s method. This corresponds to Step 4 in the workflow diagram.

####  Reverse MR Analysis of Blood eQTLs and Immune Cells

 Using each immune cell as exposure, we applied screening criteria similar to the previous section, and conducted reverse MR analysis for each immune cell with blood eQTLs as the outcome. The evaluation was performed using the MR-IVW, MR-Egger, weighted median, simple mode, and weighted mode methods. This corresponds to Step 5 in the workflow diagram.

####  Colocalization Analysis of Blood eQTLs and Immune Cells

 We conducted coloc analysis for all blood eQTL genes and 731 immune cells using Bayesian-Based colocalization (version 5.2.2). This method was based on five hypotheses: 1) no association with either trait; 2) association with trait 1 only; 3) association with trait 2 only; 4) association with both traits but different causal variants; 5) association with both traits, and both traits share the same causal variant. This corresponds to PPH0, PPH1, PPH2, PPH3, and PPH4. Generally, PPH4 > 0.5 was considered indicative of colocalization effects (i.e. the two traits share the same mutated region). This corresponds to Step 6 of the workflow diagram.

####  Sensitivity Analyses and Pleiotropy Assessment

 Because many cis instruments were single-SNP or very sparse, pleiotropy-robust estimators that require multiple variants (MR-Egger, weighted median/mode, MR-PRESSO) were not applied. Single-SNP exposures were estimated using the Wald ratio and evaluated via colocalization (PPH4), HEIDI in the SMR framework, and Steiger directionality. For the few loci with ≥ 2 variants, Cochran’s Q and Egger intercept were not reported where degrees of freedom were insufficient or results would be underpowered/misleading. This triangulation mitigates LD confounding and reverse causation while avoiding under-identified regressions in sparse settings. In line with the instrument counts, MR-Egger/weighted-median/mode (and MR-PRESSO) were not applicable to most genes and thus not reported. For single-SNP estimates, we relied on Wald ratio plus colocalization (PPH4), HEIDI (for SMR), and Steiger as robustness checks. Where only two variants were available, Egger intercept testing is not identifiable and Q is minimally informative; these diagnostics were therefore omitted to avoid over-interpretation.

## Results

###  Causal Effects of Adrenal Genes on NB

**Figure 2:**
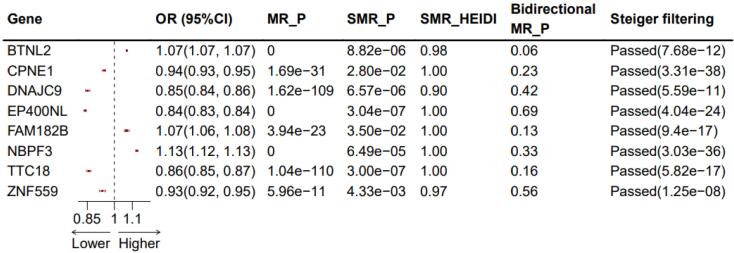
Results of the Significantly Associated Adrenal Gland Genes with NB after Correction. OR: odds ratios; MR_P, Corrected *P*-values of MR results; SMP_P, Corrected *P*-values of SMR results; SMR_HEIDI, SMR heterogeneity results; Bidirectional MR_P, *P* values of bidirectional MR results; Steiger filtering, Steiger directionality verification and associated *P* values

 After Bonferroni correction for multiple testing (*P* < 5.63 × 10^−5^) in the positive MR analysis, 257 genes were selected (Supplementary File, Table S3). The filtering criteria for the negative MR analysis were set at *P* > 0.05 (Supplementary File, Table S4), resulting in 43 genes. Subsequently, SMR analysis was performed with FDR < 0.05 and heterogeneity test p_HEIDI > 0.05 (Supplementary File, Table S5), yielding a final set of eight genes. These genes successfully passed the Steiger directionality verification (Supplementary File, Table S6) and phenotype scanning (Supplementary File, Table S7). The summarized results are depicted in Figure 2. Notably, only the final results are presented, with all intermediate results retained for reference.

**Figure 3:**
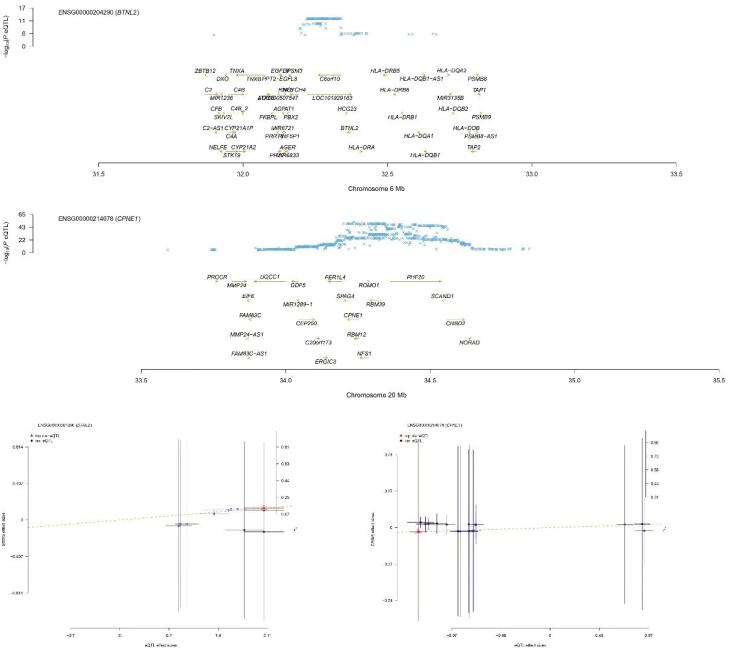
*BTNL2* and *CPNE1* SMR results plot, with the upper panel displaying the consistency of the genetic effect of the *BTNL2/CPNE1* gene with NB and the lower panel displaying a significant causal relationship between *BTNL2/CPNE1* and NB. It is also evident that the upregulation of *BTNL2/CPNE1* expression promotes the occurrence of NB (positive slope)

 Among the eight genes identified, five genes exhibited that upregulation inhibited the occurrence of NB (*CPNE1, ZNF559, TTC18, DNAJC9, *and* EP400NL*, with odds ratio (OR) values less than 1). However, the upregulation of the remaining three genes promoted the occurrence of NB. The results of MR may conflict with those of SMR, explaining the methodological differences between the two approaches, but both can demonstrate a causal effect with NB. The SMR results are depicted in Figure 3 (only the* BTNL2 and CPNE1* genes are listed; for more details, refer to Supplementary File, Figure S1).

###  Blood-associated Genes Affected NB Occurrence by Influencing Immune Cells

 We conducted a two-sample MR analysis with each immune cell type as the exposure and NB as the outcome (Step 1). The analysis was performed under the corrected *P* < 5e‒8 criterion, resulting in 352 immune cells with a causal effect on NB (Supplementary File, Table S8). After conducting reverse MR with *P* > 0.05, 291 immune cells were obtained (Supplementary File, Table S9), excluding 61 immune cells with a reverse causal effect on NB. Using blood eQTL as the exposure and NB as the outcome, we excluded genes with a direct causal effect on blood and NB, resulting in 3,175 genes (Supplementary File, Table S10). Performing MR analysis with these 3,175 blood genes as exposure and the previously identified 291 immune cells as the outcome, we identified 372 genes with a causal relationship under the criterion of corrected *P* < 5e‒8 (Supplementary File, Table S11). We further excluded immune cells with a reverse causal effect on these genes (Supplementary File, Table S12), ultimately identifying 43 genes associated with 54 immune cells affecting NB. Finally, among these genes and immune cells, we found eight genes and seven immune cells with colocalization effects. Notably, all intermediate results are retained in the final results.

**Figure 4:**
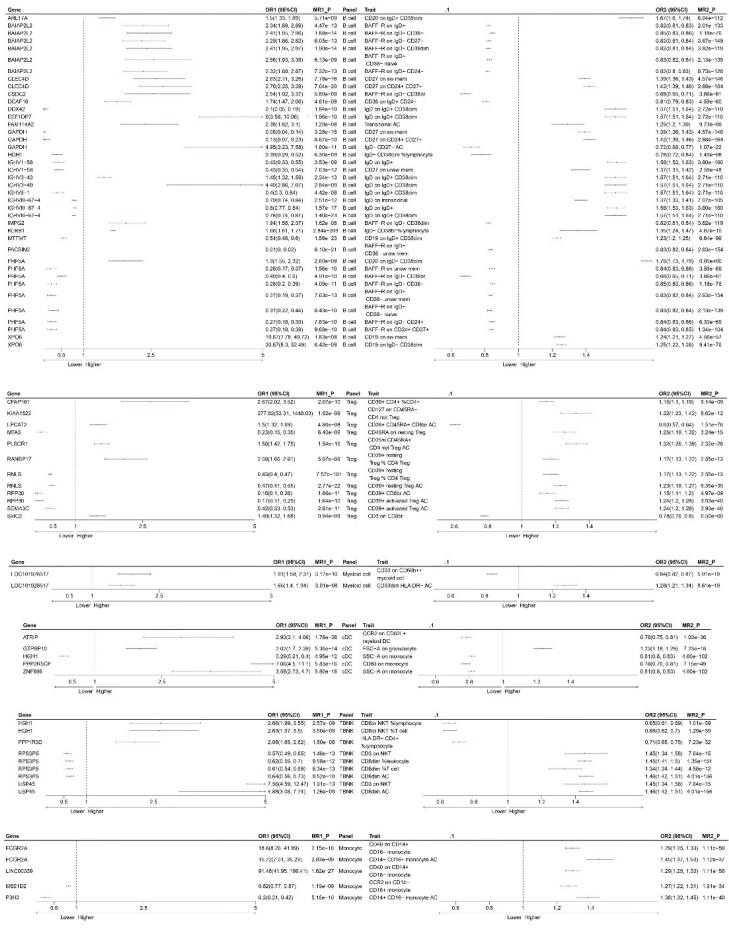
MR Results Mediated by Immune Cells. OR, odds ratios. The left forest plot represents the MR between the blood genes and immune cells, with MR1_P indicating the corrected *p*-value. The right forest plot represents the MR between immune cells and NB, with MR1_P indicating the corrected *P*-value

 We finally identified six major immune cell types (B cell, cDC, monocyte, myeloid cell, TBNK, and Treg) with significant causal effects on NB (Supplementary File, Table S13). The results for each immune cell type are displayed in Figure 3. To interpret these results, taking the first row as an example, CFAP161 gene upregulation leads to an increase in ‘CD39 + CD4 + %CD4 + ’ (OR1 > 1), subsequently increasing the odds of NB (OR2 > 1). We further conducted MR analyses mediated by immune cells, and Figure 4 summarizes the MR estimates between blood genes, immune cell subsets, and NB, with MR1_P indicating the corrected p-value.

**Figure 5:**
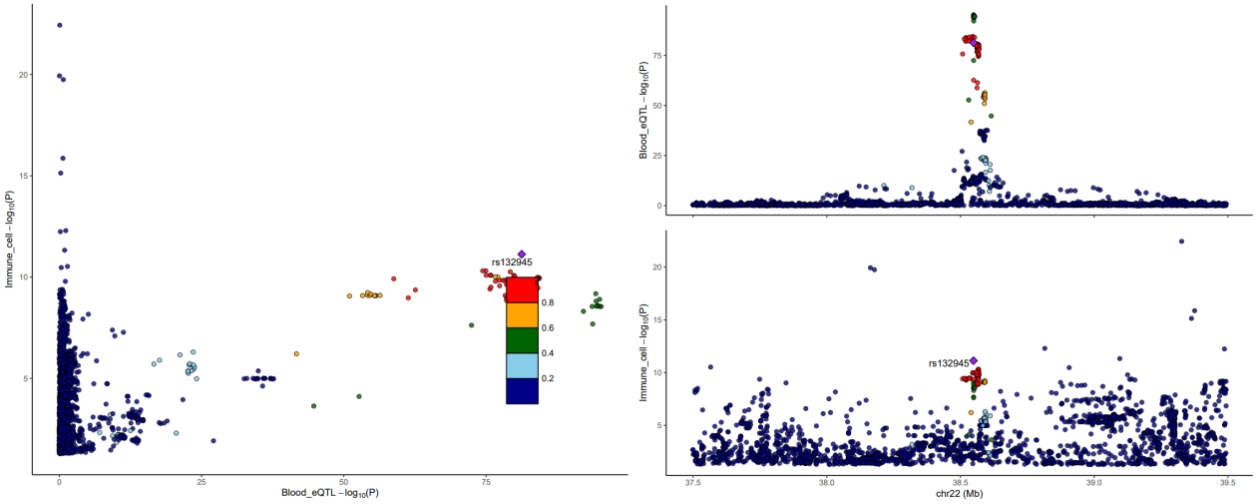
Colocalization Results between SEMA3C and CD39 + Activated Treg AC Cells. r2 indicates LD between the variants and top SNP. The left panel exhibits the colocalization results, the upper right panel displays the distribution of SNPs in blood genes, and the lower right panel displays the distribution of SNPs in immune cells

 The results of the colocalization analysis depict strong colocalization of *PLSCR1, FAM114A2,* semaphorin (*SEMA)3C, RPP30, CLEC4D, IGHV3-43, IGHV3-49, *and* BAIAP2L2* with Treg and B cells. These genes may serve as potential drug targets (Figure 5) (only listing SEMA3C and CD39 + activated Treg AC cells; for more details, refer to Supplementary Figure S2.

## Discussion

 This is the first study to combine adrenal cis-eQTL, blood cis-eQTL, and immune cell GWAS data to explore potential therapeutic targets for NB using two-sample MR and Bayesian colocalization techniques. Ultimately, we identified eight adrenal genes associated with NB, where the upregulation of five genes (*CPNE1, ZNF559, TTC18, DNAJC9, *and* EP400NL*) inhibited the occurrence of NB. In comparison, the upregulation of three genes (*BTNL2, FAM182B, *and* NBPF3*) was associated with promoting NB. The bidirectional MR results suggest that BTNL2 has the strongest causal effect on NB occurrence (bidirectional MR_P = 0.06) (Figure 2). Furthermore, we identified 43 blood-related genes that, through their influence on six major immune cell categories (B cell, cDC, Monocyte, Myeloid cell, TBNK, and Treg), had significant causal effects on NB. Colocalization analysis revealed strong colocalization of *PLSCR1, FAM114A2, SEMA3C, RPP30, CLEC4D, IGHV3-43, IGHV3-49, *and* BAIAP2L2* with Tregs and B cells, suggesting that these genes could be potential drug targets.

 In this study, several genes, including *BTNL2, SEMA3C,* and* RANBP17*, identified as significant candidates associated with NB, were experimentally validated to be associated with the occurrence and development of various tumors.^30-32^ However, there is a causal relationship between these genes and NB occurrence that has not yet been investigated. Our study serves as an essential supplement to existing research.

*SEMA3C* plays a crucial role in NB and regulates tumor cell adhesion, migration, and metastasis through its interaction with neuropilin and plexin receptor complexes. Studies have found an inverse correlation between the expression levels of *SEMA3C *in NB cells and the invasiveness and metastatic potential of the disease, suggesting its potential role as a tumor metastasis suppressor.^33^ In chicken embryo models, decreased expression of *SEMA3C *is associated with impaired collective migration of NB cells and their ability to localize towards sympathetic structures, further supporting its critical role in tumor metastasis.^34^ Moreover, *SEMA3C *expression is regulated by microRNA-34a, providing a new perspective for developing therapeutic strategies targeting *SEMA3C*.^32^ In the two-step MR analysis, we found a negative correlation between the expression of *SEMA3C *and CD39 + -activated Treg cells.

 Moreover, the expression of CD39 + -activated Treg cells is a risk factor for the occurrence of NB (Figure 4). Further colocalization analysis confirmed the association between *SEMA3C *and CD39 + -activated Treg cells (Figure 4). We hypothesized that *SEMA3C *may suppress NB occurrence by inhibiting the expression of CD39 + -activated Treg cells, which requires further experimental investigation. These findings indicate that *SEMA3C *is a promising biomarker in NB research and a potential target for developing new therapies, contributing to improving patient treatment and prognosis. Simultaneously, several gene-testing organizations have identified *SEMA3C *as among the sensitivity gene targets for NB chemotherapy.

 Butyrophilin-like protein 2 (*BTNL2)* is a protein that plays a role in the immune system and belongs to the butyrophilin family. Studies indicate that *BTNL2* can inhibit T-cell activation, a function that may be linked to its role in immune tolerance.^35^ BTNL2 expression is associated with immune evasion and tumor progression in the tumor microenvironment. Research has found that *BTNL2* can promote IL-17A production, thereby enhancing tumor immune evasion.^36^ In addition,* BTNL2 *is associated with tumor cell proliferation and resistance to apoptosis, possibly by activating the STAT3 signaling pathway to promote tumor growth.^37^ In a colorectal cancer model, *BTNL2* loss significantly reduced the tumor burden, suggesting that *BTNL2* may be a potential target for cancer treatment.^38^

 Regarding treatment, monoclonal antibody therapy targeting* BTNL2* has been depicted to reduce the burden of mouse colon tumors significantly. In this study,* BTNL2* was identified to be among the genes associated with NB, indicating its potential involvement in the immune evasion mechanisms of NB. These findings suggest that NB cells can evade immune system surveillance through various mechanisms, including altering the function of immune cells and expressing immune inhibitory molecules. As an immune checkpoint protein, *BTNL2* may inhibit T-cell activation to promote tumor growth and spread. In NB cells, *BTNL2* may regulate the immune response of tumors by influencing immune cells in the tumor microenvironment, such as Tregs. This immune regulatory effect may impact the therapeutic strategies for NB, specifically in developing immunotherapies targeting immune checkpoints. This study established the association between *BTNL2* and NB occurrence through SMR analysis (SMR *P*< 0.001; OR = 1.07, 95% confidence interval [1.07 ~ 1.07]) (Figure 2). Future research needs to explore the specific mechanisms of *BTNL2* in NB and how it influences the immune microenvironment of tumors. This may include investigating how *BTNL2* affects the interaction between NB and immune cells and whether it can serve as a new target for NB treatment.

 Ran GTP-binding protein 17 (*RANBP17*) is a protein that plays a crucial role in nucleocytoplasmic transport and belongs to the importin beta superfamily. It functions in various biological processes, including cell proliferation, apoptosis control, and nucleocytoplasmic transport.^39^ Its significance in head and neck squamous cell carcinoma (HNSCC) is particularly notable, as studies suggest that its expression level in HNSCC cell lines is closely associated with cell proliferation.^31^ The *RANBP17* expression level decreases after treatment with the chemotherapeutic drug cisplatin but increases in tumor cell lines, indicating that *RANBP17* may be involved in cell sensitivity to chemotherapy. Despite the confirmed association between *RANBP17* and cell proliferation, further research is needed to elucidate its specific roles and regulatory mechanisms during the cell cycle. Although *RANBP17* has been studied in other types of cancer, such as HNSCC, its specific roles and potential clinical significance in NB require further investigation. As a key regulatory factor in nucleocytoplasmic transport, *RANBP17* may play a role in the cell proliferation, differentiation, and apoptosis processes of NB. Given its importance in cell cycle regulation, *RANBP17* may influence the proliferation rate of NB cells and their responses to treatment. For example, *RANBP17* may affect the expression and activity of cell cycle proteins by regulating intracellular signaling pathways such as the Ran GTPase cycle, thereby affecting the growth and division of tumor cells.^31,39^ This study revealed that *RANBP17* expression was positively correlated with the expression of CD39 + resting Treg % CD4 Treg (Figure 4). Simultaneously, CD39 + resting Treg % CD4 Treg expression was positively associated with NB occurrence. We speculate that *RANBP17* may promote the occurrence of NB by enhancing the expression of CD39 + resting Treg % CD4 Treg. This study provides new insight into how *RANBP17* influences NB occurrence. Understanding the role of *RANBP17* in NB may contribute to developing novel therapeutic targets. Inhibitors targeting *RANBP17* could potentially slow tumor growth or enhance the effectiveness of chemotherapy. However, current research on the specific roles and clinical applications of *RANBP17* in NB is limited. Future studies should employ high-throughput sequencing, proteomic analysis, and functional validation to elucidate the specific mechanisms of *RANBP17* in developing NB and its potential as a therapeutic target.

 Our study has several limitations. First, only two were selected because of the limited sources of GWAS datasets for NB, with only six relevant datasets available in the OpenGWAS database and excluding datasets with poor data quality. Additionally, the sample size of the datasets was small, and all were of European descent, which may have introduced corresponding biases. Second, all genes had only one cis-acting SNP and lacked trans-eQTLs, limiting the application of analyses, including alternative MR algorithms, heterogeneity tests, and pleiotropy tests. Third, while MR provides evidence of causality, its effectiveness relies on strict assumptions such as the independence, relevance, and validity of instrumental variables.^40^ Our outcome GWAS and eQTL sources are European-ancestry, which constrains external validity. Ancestry differences in allele frequencies and LD patterns can alter instrument strength and harmonization; moreover, cis-eQTL effect sizes and even lead variants may differ across populations, affecting colocalization and causal inference. We therefore interpret our results as hypothesis-generating for European populations and explicitly prioritize cross-ancestry validation as a next step—ideally using ancestry-matched eQTL resources, trans-ethnic colocalization, and MR frameworks that model heterogeneity across ancestries. Until such evidence is available, therapeutic extrapolation beyond European populations should be made with caution. The cis-MR design yields sparse instruments (often single-SNP), which precludes standard pleiotropy-robust estimators and formal intercept tests in most loci. We therefore interpret the findings as hypothesis-generating, supported by triangulation (Wald ratio + colocalization/HEIDI + Steiger), and emphasize the need for functional validation.

## Conclusion

 In conclusion, our study is the first to utilize MR methods to investigate the relationship between adrenal genes, blood genes, immune cells, and NB while uncovering potential therapeutic targets. This pioneering study opens innovative avenues for treating high-risk patients with NB. Simultaneously, this study provides new research directions for studying the occurrence of NB. Future research may require validation and exploration of the precise mechanisms. These findings prioritize candidate genes for NB risk in European ancestry, and will require independent replication in non-European cohorts and ancestry-matched functional datasets before broader generalization.

## Supplementary Files

Supplementary File 1 contains Tables S1-S13 and Figures S1-S2.
