# Supplementary material for: Potential Therapeutic Targets for Neuroblastoma Screened through Mendelian Randomization Analysis
Source: Arch Iran Med. 2025 Nov 1;28(11):642–51. doi: 10.34172/aim.35114 (PMC12958419; doi:10.34172/aim.35114)
Supplement: Supplementary file 1 — contains Tables S1-S13 and Figures S1-S2. [file aim-28-642-s001.pdf]

Table S1. STROBE–MR checklist for the reporting of Mendelian randomization analyses in the present study.

Section	Item	Recommendation	Reported? (Yes/No/NA)	Location (section / page-line)	Notes
Title/ Abstract	MR design stated	State MR design (two-sample; cis-eQTL instruments) in title/abstract.	Yes	Abstract — opening line	Keep concise; no extra methods here.
Title/ Abstract	Objectives & hypotheses	State primary objective and that findings are hypothesis-generating.	Yes	Introduction — final paragraph; Abstract — conclusions	Tempered claims ('evidence consistent with').
Introduction	Background & research gap	Explain NB genetics and the unresolved causal role of cis-expression.	Yes	Introduction — early paragraphs	Gap: which genes/tissues mediate risk.
Introduction	MR rationale & assumptions	Explain why MR fits; list relevance, independence, exclusion restriction.	Yes	Introduction — MR paragraph	Compact assumptions sentence.
Methods	Study design overview	Two-sample/ bidirectional design; SMR/coloc mentioned.	Yes	Methods — Study design	Bidirectional + Steiger + SMR/coloc.
Methods	Data sources & IDs	List dataset IDs and URLs for GWAS/eQTL.	Yes	Methods — Data sources	ieu-a-816; prot-a-2003; GTEx v8; eQTLGen.
Methods	Sample sizes &	Report	Yes	Methods —	NB:

	ancestry	ncases/ncontrols and ancestry for all sources.		Data sources	1,627/3,254; 3,301; European. GTEx=233; eQTLGen=31,684.
Methods	Eligibility/ inclusion (per-source)	Describe inclusion criteria or cite source methods.	NA	Methods — Data sources (citations)	Summary-level; defer to source GWAS/eQTL papers.
Methods	Exposure definition & tissue justification	Define cis-eQTL exposures; justify adrenal/blood/immune selection.	Yes	Methods — Instruments & Tissue rationale	Sympathoadrenal lineage; immune complement.
Methods	Instrument selection & LD clumping	Genome-wide significance; cis window; LD r^2 and kb thresholds.	Yes	Methods — Instrument selection	State p-threshold and clumping.
Methods	Harmonization & palindromic SNPs	Allele alignment; handling of palindromic SNPs/strand.	No	Methods — Harmonization (add 1 sentence)	Minimal add: allele alignment and palindromic handling.
Methods	Instrument strength	Per-SNP $F \approx Z^2$; thresholds; note single-SNP usage.	Yes	Methods — Instrument strength	WR for single-SNP; IVW for multi-SNP.
Methods	Sample overlap assessment	Assess exposure–outcome overlap; mitigation.	Yes	Methods — Sample overlap	No intentional overlap; meta-analysis of two NB GWAS.
Methods	Population	How ancestry	NA	Methods —	Handled by

	stratification	confounding is addressed or cited.		Data sources (citations)	source GWAS/eQTL; cite PCs/controls.
Methods	Primary estimator	State IVW (multiplicative RE) and WR for single-SNP.	Yes	Methods — Statistical reporting	Already clarified.
Methods	Sensitivity analyses (applicability)	Specify when Egger/median/mode/PRESSO apply; single-SNP NA.	Yes	Methods — Sensitivity analyses	Applicability explicit.
Methods	Pleiotropy & heterogeneity metrics	Q, Egger intercept where eligible; otherwise NA.	Yes	Results/ Methods — Sensitivity & pleiotropy	Diagnostics provided or inapplicable.
Methods	Directionality	Steiger directionality test.	Yes	Methods — Study design/Validation	Steiger used.
Methods	Colocalization / HEIDI	Report coloc priors/PPH4 and SMR HEIDI.	Yes	Methods — Colocalization / SMR	PPH4; HEIDI P.
Methods	Multiple testing	Define unified FDR strategy; explain Bonferroni/descriptive steps.	Yes	Methods — Multiple testing	Tiered plan; q<0.05 within families.
Methods	Software & versions	List software/packages and versions.	No	Methods — Software (add 1 line)	Add R version & key packages.
Results	Instrument flow	Counts before/after filtering, per	No	Results — add 1 line or	Optional; may remain in

		gene/tissue.		Supplement	Supplement.
Results	Main estimates reported	OR, 95% CI, p, q, nSNPs, estimator per gene.	Yes	Results — Table 2; Abstract (selected)	Now included.
Results	Sensitivity outcomes	Summarize Q/Egger/LOO where eligible; otherwise NA.	Yes	Results — Sensitivity	Aligned with applicability.
Results	Validation	External validation or triangulation.	Yes	Methods/ Results — Triangulatio n	Coloc/HEIDI/ Steiger (no GEO).
Discussion	Interpretation (hypothesis-generating)	Avoid overstatement; therapeutic claims tempered.	Yes	Discussion — Interpretatio n & limitations; Abstract — conclusions	Putative/ potential targets.
Discussion	Limitations	Single-SNP, GWAS size, pleiotropy, coloc uncertainty.	Yes	Discussion — Limitations	Explicitly listed.
Discussion	Generalisability	Ancestry/ transportability to non-European populations.	Yes	Discussion — Generalisabil ity	Call for cross-ancestry replication.
Discussion	Implications & next steps	Functional/ pharmacological validation; replication.	Yes	Conclusions — final sentence	Brief.
Other	Funding & conflicts	Declare funding and conflicts/competing	Author to	End matter / Acknowledge	Journal standard.

		interests.	confirm	ments	

Table S2. STREGA checklist for the reporting of genetic association results in the present study.

Section	Item	Recommendation	Reported? (Yes/No/NA)	Location (section / page-line)	Notes
Title/ Abstract	Study design	State genetic association/MR context (summary-level).	Yes	Abstract — design; Methods — overview	MR using GWAS/eQTL summaries.
Methods	Participants & setting	Describe recruitment/eligibility; for summaries, cite source.	NA	Methods — Data sources (citations)	Defer to source GWAS/eQTL papers.
Methods	Ancestry/ population	Report ancestry of each dataset.	Yes	Methods — Data sources	European ancestry for NB GWAS; GTEx/eQTLGen noted.
Methods	Genotyping & imputation	Platforms, QC, imputation methods; cite source studies.	NA	Methods — Data sources (citations)	Handled in source.
Methods	Marker IDs & genome build	Reference build and variant identifiers (or cite source).	NA	Methods — Data sources (citations)	Inherit from GWAS/eQTL resources.
Methods	HWE & allele frequencies	Report or cite from source GWAS.	NA	Methods — Data sources (citations)	Source QC.

Methods	Population stratification control	PC adjustment/stratification; cite source GWAS.	NA	Methods — Data sources (citations)	Handled in source analyses.
Methods	Relatedness / cryptic relatedness	How related samples were addressed in source GWAS.	NA	Methods — Data sources (citations)	Source QC.
Methods	Multiple comparisons	Define approach (FDR within families; Bonferroni descriptive).	Yes	Methods — Multiple testing	Tiered plan stated.
Methods	Statistical methods	IVW/WR/SMR; colocalization; Steiger; HEIDI.	Yes	Methods — Statistical reporting	Stated.
Methods	Software & versions	List software/packages and versions.	No	Methods — Software (add 1 line)	Add R version & packages.
Results	Descriptive results	Sample sizes by dataset; ancestry.	Yes	Methods — Data sources; Abstract (compact)	Present in Methods.
Results	Main findings	Effect sizes with uncertainty and multiplicity.	Yes	Results — Table 2; Abstract (selected)	OR, 95% CI, p, q.
Results	Subgroup/ interaction analyses	If done; otherwise state NA.	NA	—	Not performed.
Results	Sensitivity analyses	Applicability; diagnostics where eligible.	Yes	Results — Sensitivity	Aligned with instrument counts.

Discussion	Limitations	Bias, weak instruments, ancestry, generalisability.	Yes	Discussion — Limitations/Generalisability	Noted.
Other	Data/code availability	Where to access summary data/code.	No	End matter / Data availability	Add URLs (OpenGWAS, GTEx, eQTLGen).
Other	Funding/COI	Funding sources and conflicts.	No Author to confirm	End matter	Journal standard.

Table S3. Primary MR estimates for the association between blood gene expression and neuroblastoma risk.

gene	or	pvalue	se	or_lci	or_uci	beta	FDR
NBPF3	1.125416	0	0.002909	1.119018	1.131851	0.118153	0
BTNL2	1.068641	0	0.000512	1.06757	1.069714	0.066388	0
DNAJC9	0.85196	6.09E-113	0.007094	0.840197	0.863889	-0.16022	1.62E-109
TTC18	0.858429	3.92E-114	0.006723	0.847191	0.869815	-0.15265	1.04E-110
EP400NL	0.83788	0	0.003729	0.831779	0.844026	-0.17688	0
ZNF559	0.93364	2.24E-14	0.008992	0.917329	0.950241	-0.06866	5.96E-11
FAM182B	1.068721	1.48E-26	0.006232	1.055747	1.081855	0.066463	3.95E-23
CPNE1	0.943001	6.36E-35	0.00476	0.934244	0.951841	-0.05869	1.69E-31

Table S4. Two-sample MR estimates for the association between candidate genes and neuroblastoma using different MR methods.

Gene	method	nsnp	b	se	pval	lo_ci	up_ci	or	or_lci95	or_uci95
NBPF3	MR Egger	136	11.39048	8.094786	0.161702	-4.4753	27.25626	88475.64	0.011387	6.87E+11
NBPF3	Weighted median	136	8.28021	0.931409	6.11E-19	6.454648	10.10577	3945.024	635.65	24483.94
NBPF3	Inverse variance weighted	136	2.721498	2.698861	0.313268	-2.56827	8.011266	15.20309	0.076668	3014.731
NBPF3	Simple mode	136	35.29653	36.05045	0.329288	-35.3624	105.9554	2.13E+15	4.39E-16	1.04E+46
NBPF3	Weighted mode	136	7.821407	3.530727	0.02842	0.901183	14.74163	2493.412	2.462514	2524697
BTNL2	MR Egger	174	11.9367	6.365826	0.062471	-0.54032	24.41372	152772	0.582564	4.01E+10
BTNL2	Weighted median	174	15.38735	0.313661	0	14.77257	16.00213	4815499	2604039	8905026
BTNL2	Inverse variance weighted	174	3.801582	1.106675	0.000592	1.6325	5.970664	44.77197	5.11665	391.7659
BTNL2	Simple mode	174	15.49751	0.676301	2.72E-54	14.17196	16.82306	5376280	1428245	20237702
BTNL2	Weighted mode	174	15.40641	0.707143	1.81E-51	14.02041	16.79241	4908156	1227401	19626837
DNAJC9	MR Egger	327	-3.7814	4.640248	0.415719	-12.8763	5.313486	0.022791	2.56E-06	203.0568
DNAJC9	Weighted median	327	-6.31993	0.943878	2.15E-11	-8.16993	-4.46993	0.0018	0.000283	0.011448
DNAJC9	Inverse variance weighted	327	1.964701	0.519566	0.000156	0.946351	2.983051	7.132778	2.576291	19.74797
DNAJC9	Simple mode	327	9.251063	1.511508	2.68E-09	6.288506	12.21362	10415.63	538.3486	201515.1
DNAJC9	Weighted mode	327	-6.26248	1.02127	2.51E-09	-8.26417	-4.26079	0.001907	0.000258	0.014111
TTC18	MR Egger	708	-4.14934	2.947538	0.159651	-9.92651	1.627833	0.015775	4.89E-05	5.092826
TTC18	Weighted median	708	-5.88592	0.440819	1.15E-40	-6.74992	-5.02191	0.002778	0.001171	0.006592
TTC18	Inverse variance weighted	708	1.708761	0.454948	0.000173	0.817063	2.60046	5.522117	2.263841	13.46993
TTC18	Simple mode	708	13.87216	1.341977	2.02E-23	11.24188	16.50243	1058280	76258.25	14686375
TTC18	Weighted mode	708	-5.92018	1.142265	2.85E-07	-8.15902	-3.68134	0.002685	0.000286	0.025189
EP400NL	MR Egger	98	-1.21644	3.09086	0.694778	-7.27453	4.841643	0.296282	0.000693	126.6773
EP400NL	Weighted median	98	-5.86979	0.601146	1.60E-22	-7.04804	-4.69155	0.002823	0.000869	0.009173
EP400NL	Inverse variance weighted	98	-1.5206	1.167113	0.192619	-3.80814	0.766946	0.218582	0.022189	2.153181
EP400NL	Simple mode	98	-11.3476	5.516104	0.042352	-22.1592	-0.53608	1.18E-05	2.38E-10	0.585035
EP400NL	Weighted mode	98	-5.95853	1.214971	3.77E-06	-8.33987	-3.57718	0.002584	0.000239	0.027954
ZNF559	MR Egger	20	-7.39842	12.46479	0.560202	-31.8294	17.03257	0.000612	1.50E-14	24954711

ZNF559	Weighted median	20	-11.3878	1.767081	1.16E-10	-14.8513	-7.92434	1.13E-05	3.55E-07	0.000362
ZNF559	Inverse variance weighted	20	-4.11867	4.137427	0.319509	-12.228	3.990685	0.016266	4.89E-06	54.09191
ZNF559	Simple mode	20	-17.8515	2.962957	8.52E-06	-23.6589	-12.0441	1.77E-08	5.31E-11	5.88E-06
ZNF559	Weighted mode	20	-12.1401	1.677487	7.18E-07	-15.428	-8.85222	5.34E-06	1.99E-07	0.000143
FAM182B	MR Egger	430	10.45416	3.260038	0.001443	4.064484	16.84383	34688.34	58.23485	20662555
FAM182B	Weighted median	430	14.21291	0.842066	6.46E-64	12.56246	15.86336	1487954	285633.1	7751224
FAM182B	Inverse variance weighted	430	-1.45938	0.97332	0.133775	-3.36708	0.448332	0.232381	0.03449	1.565698
FAM182B	Simple mode	430	17.02863	5.887798	0.00402	5.488543	28.56871	24856435	241.9044	2.55E+12
FAM182B	Weighted mode	430	14.20587	2.919542	1.60E-06	8.483564	19.92817	1477505	4834.65	4.52E+08
CPNE1	MR Egger	826	-21.3937	4.500746	2.36E-06	-30.2152	-12.5723	5.11E-10	7.55E-14	3.47E-06
CPNE1	Weighted median	826	-13.1928	1.481895	5.45E-19	-16.0974	-10.2883	1.86E-06	1.02E-07	3.40E-05
CPNE1	Inverse variance weighted	826	-0.99563	0.829887	0.230248	-2.62221	0.630947	0.36949	0.072642	1.879389
CPNE1	Simple mode	826	22.98745	7.352268	0.001831	8.577003	37.3979	9.62E+09	5308.173	1.74E+16
CPNE1	Weighted mode	826	-13.734	2.862117	1.90E-06	-19.3437	-8.1242	1.08E-06	3.97E-09	0.000296

Table S5. Summary of SMR and HEIDI tests identifying genes whose blood expression is associated with neuroblastoma risk.

probeID	ProbeChr	Gene	Probe_bp	topSNP	topSNP_chr	topSNP_bp	A1	A2	Freq	b_GWAS	se_GWAS	p_GWAS	b_eQTL	se_eQT[illegible]
ENSG00000142794	1	NBPF3	21766621	rs4654948	1	21805978	A	G	0.771372	0.0291	0.7724	0.969947	-1.20638	0.08[illegible]
ENSG00000204290	6	BTNL2	32361740	rs28699544	6	32338172	C	G	0.006958	0.1336	0.0087	3.21E-53	2.05575	0.2[illegible]
ENSG00000213551	10	DNAJC9	74943120	rs150505066	10	75310757	A	G	0.008946	0.1671	0.0074	6.650E-113	-1.04133	0.15[illegible]
ENSG00000156042	10	TTC18	75013517	rs12265144	10	75123399	A	G	0.063618	0.0498	0.0641	0.437211	-0.7592	0.08[illegible]
ENSG00000185684	12	EP400NL	1.33E+08	rs61942869	12	1.33E+08	A	G	0.136183	0.0373	0.1301	0.77434	-0.62666	0.05[illegible]
ENSG00000188321	19	ZNF559	9434448	rs28384491	19	9434872	T	C	0.086481	0.0414	0.0983	0.673639	-1.14125	0.0[illegible]
ENSG00000175170	20	FAM182B	25744102	rs6037263	20	25870227	G	A	0.32008	-0.0272	0.6848	0.968317	0.946412	0.09[illegible]
ENSG00000214078	20	CPNE1	34213953	rs6060535	20	34235522	T	C	0.101392	0.0415	0.098	0.671953	-1.29403	0.08[illegible]

Table S6. Steiger filtering results for instrumental variables used in the MR analyses between blood gene expression and neuroblastoma.

effect_allele.exposure	other_allele.exposure	effect_allele.outcome	other_allele.outcome	samplesize.outcome	mr_keep.outcome	pval_origin.outcome	samplesize.exposure	mr_keep.exposure	pval_origin.exposure	effective_n.exposure	effective_n.outcome
A	AATTT	A	AATTT	81182	TRUE	reported	2333	TRUE	reported	2333	81182
C	CCT	C	CCT	81182	TRUE	repo	2333	TRUE	repo	2333	81182

						rted			rted		
CAT	C	CAT	C	8182	TRUE	reported	233	TRUE	reported	233	8182
ACT	A	ACT	A	8182	TRUE	reported	233	TRUE	reported	233	8182
GCCC	G	GCCC	G	8182	TRUE	reported	233	TRUE	reported	233	8182
TCA	T	TCA	T	8182	TRUE	repor	233	TRUE	repor	233	8182

						ted			ted		
CTA	C	CTA	C	8182	TRUE	reported	233	TRUE	reported	233	8182
AGTTT	A	AGTTT	A	8182	TRUE	reported	233	TRUE	reported	233	8182
CAA	C	CAA	C	8182	TRUE	reported	233	TRUE	reported	233	8182
TG	T	TG	T	8182	TRUE	report	233	TRUE	report	233	8182

						ed			ed		
GTA	G	GTA	G	8182	TRUE	reported	233	TRUE	reported	233	8182
CA	C	CA	C	8182	TRUE	reported	233	TRUE	reported	233	8182
ATTATT	A	ATTATT	A	8182	TRUE	reported	233	TRUE	reported	233	8182
T	TA	T	TA	8182	TRUE	reporte	233	TRUE	reporte	233	8182

						d			d		
CT	C	CT	C	8182	TRUE	reported	233	TRUE	reported	233	8182
TG	T	TG	T	8182	TRUE	reported	233	TRUE	reported	233	8182
C	CGCCAG	C	CGCCAG	8182	TRUE	reported	233	TRUE	reported	233	8182
C	CT	C	CT	8182	TRUE	reporte	233	TRUE	reporte	233	8182

						d			d		
ATCTCAT	A	ATCTCAT	A	8182	TRUE	reported	233	TRUE	reported	233	8182
TG	T	TG	T	8182	TRUE	reported	233	TRUE	reported	233	8182
C	CG	C	CG	8182	TRUE	reported	233	TRUE	reported	233	8182
TAGC	T	TAGC	T	8182	TRUE	reported	233	TRUE	reported	233	8182

GGCCA	G	GGCCA	G	8182	TRUE	reported	233	TRUE	reported	233	8182
A	AT	A	AT	8182	TRUE	reported	233	TRUE	reported	233	8182
AG	A	AG	A	8182	TRUE	reported	233	TRUE	reported	233	8182
T	TTA	T	TTA	8182	TRUE	reported	233	TRUE	reported	233	8182
G	GC	G	GC	8	T	r	2	T	r	2	8

				182	RUE	eported	33	RUE	eported	33	182
GA	G	GA	G	8182	TRUE	reported	233	TRUE	reported	233	8182
CT	C	CT	C	8182	TRUE	reported	233	TRUE	reported	233	8182
TG	T	TG	T	8182	TRUE	reported	233	TRUE	reported	233	8182
T	A	T	A	8	T	r	2	T	r	2	8

e e
p p
1 R o R o 1
3 3
8 U r U r 8
3 3
2 E t E t 2
e e
d d
r r
e e
8 T p T p 8
2 2
1 R o R o 1
G A G A 3 3
8 U r U r 8
3 3
2 E t E t 2
e e
d d
r r
e e
8 T p T p 8
2 2
1 R o R o 1
A G A G 3 3
8 U r U r 8
3 3
2 E t E t 2
e e
d d
r r
e e
8 T p T p 8
2 2
1 R o R o 1
C T C T 3 3
8 U r U r 8
3 3
2 E t E t 2
e e
d d
8 T r 2 T r 2 8
T C T C
1 R e 3 R e 3 1

				82	UE	ported	3	UE	ported	3	82
A	G	A	G	8182	TRUE	reported	233	TRUE	reported	233	8182
T	C	T	C	8182	TRUE	reported	233	TRUE	reported	233	8182
GT	G	GT	G	8182	TRUE	reported	233	TRUE	reported	233	8182
A	G	A	G	818	TRU	rep	233	TRU	rep	233	818

				2	E	orted		E	orted		2
C	G	C	G	8182	TRUE	reported	233	TRUE	reported	233	8182
T	C	T	C	8182	TRUE	reported	233	TRUE	reported	233	8182
G	A	G	A	8182	TRUE	reported	233	TRUE	reported	233	8182
T	C	T	C	8182	TRUE	repo	233	TRUE	repo	233	8182

						rted			rted		
C	T	C	T	8182	TRUE	reported	233	TRUE	reported	233	8182
G	A	G	A	8182	TRUE	reported	233	TRUE	reported	233	8182
A	G	A	G	8182	TRUE	reported	233	TRUE	reported	233	8182
A	ATATTAT	A	ATATTAT	8182	TRUE	repo	233	TRUE	repo	233	8182

A	T	A	T	8182	TRUE	reported	233	TRUE	reported	233	8182
A	G	A	G	8182	TRUE	reported	233	TRUE	reported	233	8182
CAT	C	CAT	C	8182	TRUE	reported	233	TRUE	reported	233	8182
C	T	C	T	8182	TRUE	reported	233	TRUE	reported	233	8182

CTCT	C	CTCT	C	8182	TRUE	reported	233	TRUE	reported	233	8182
G	C	G	C	8182	TRUE	reported	233	TRUE	reported	233	8182
A	G	A	G	8182	TRUE	reported	233	TRUE	reported	233	8182
G	C	G	C	8182	TRUE	repor	233	TRUE	repor	233	8182

C	G	C	G	8182	TRUE	reported	233	TRUE	reported	233	8182
A	C	A	C	8182	TRUE	reported	233	TRUE	reported	233	8182
T	C	T	C	8182	TRUE	reported	233	TRUE	reported	233	8182
A	G	A	G	8182	TRUE	reported	233	TRUE	reported	233	8182

G	A	G	A	8182	TRUE	reported	233	TRUE	reported	233	8182
T	C	T	C	8182	TRUE	reported	233	TRUE	reported	233	8182
A	C	A	C	8182	TRUE	reported	233	TRUE	reported	233	8182
A	G	A	G	8182	TRUE	reported	233	TRUE	reported	233	8182

T	C	T	C	8182	TRUE	reported	233	TRUE	reported	233	8182
G	T	G	T	8182	TRUE	reported	233	TRUE	reported	233	8182
C	T	C	T	8182	TRUE	reported	233	TRUE	reported	233	8182
A	G	A	G	8182	TRUE	reported	233	TRUE	reported	233	8182

T	C	T	C	8182	TRUE	reported	233	TRUE	reported	233	8182
A	G	A	G	8182	TRUE	reported	233	TRUE	reported	233	8182
C	A	C	A	8182	TRUE	reported	233	TRUE	reported	233	8182
C	T	C	T	8182	TRUE	reported	233	TRUE	reported	233	8182

G	A	G	A	8182	TRUE	reported	233	TRUE	reported	233	8182
A	G	A	G	8182	TRUE	reported	233	TRUE	reported	233	8182
T	C	T	C	8182	TRUE	reported	233	TRUE	reported	233	8182
T	C	T	C	8182	TRUE	reported	233	TRUE	reported	233	8182

G	C	G	C	8182	TRUE	reported	233	TRUE	reported	233	8182
G	A	G	A	8182	TRUE	reported	233	TRUE	reported	233	8182
A	G	A	G	8182	TRUE	reported	233	TRUE	reported	233	8182
A	T	A	T	8182	TRUE	reported	233	TRUE	reported	233	8182

A	G	A	G	8182	TRUE	reported	233	TRUE	reported	233	8182
T	C	T	C	8182	TRUE	reported	233	TRUE	reported	233	8182
C	T	C	T	8182	TRUE	reported	233	TRUE	reported	233	8182
A	C	A	C	8182	TRUE	reported	233	TRUE	reported	233	8182

T	C	T	C	8182	TRUE	reported	233	TRUE	reported	233	8182
A	C	A	C	8182	TRUE	reported	233	TRUE	reported	233	8182
A	G	A	G	8182	TRUE	reported	233	TRUE	reported	233	8182
A	G	A	G	8182	TRUE	reported	233	TRUE	reported	233	8182

T	C	T	C	8182	TRUE	reported	233	TRUE	reported	233	8182
A	T	A	T	8182	TRUE	reported	233	TRUE	reported	233	8182
T	C	T	C	8182	TRUE	reported	233	TRUE	reported	233	8182
T	G	T	G	8182	TRUE	reported	233	TRUE	reported	233	8182

A	G	A	G	8182	TRUE	reported	233	TRUE	reported	233	8182
A	C	A	C	8182	TRUE	reported	233	TRUE	reported	233	8182
G	C	G	C	8182	TRUE	reported	233	TRUE	reported	233	8182
C	T	C	T	8182	TRUE	reported	233	TRUE	reported	233	8182

T	C	T	C	8182	TRUE	reported	233	TRUE	reported	233	8182
T	C	T	C	8182	TRUE	reported	233	TRUE	reported	233	8182
C	G	C	G	8182	TRUE	reported	233	TRUE	reported	233	8182
A	G	A	G	8182	TRUE	reported	233	TRUE	reported	233	8182

TA	T	TA	T	8182	TRUE	reported	233	TRUE	reported	233	8182
GTTA	G	GTTA	G	8182	TRUE	reported	233	TRUE	reported	233	8182
T	C	T	C	8182	TRUE	reported	233	TRUE	reported	233	8182
G	T	G	T	8182	TRUE	reported	233	TRUE	reported	233	8182

CT	C	CT	C	8182	TRUE	reported	233	TRUE	reported	233	8182
T	TCAA	T	TCAA	8182	TRUE	reported	233	TRUE	reported	233	8182
TTTTTG	T	A	AAAAACAAAACAAAAC	8182	TRUE	reported	233	TRUE	reported	233	8182
T	C	T	C	8182	TRUE	reported	233	TRUE	reported	233	8182

C	T	C	T	8182	TRUE	reported	233	TRUE	reported	233	8182
A	G	A	G	8182	TRUE	reported	233	TRUE	reported	233	8182
ATAACATACTTACCTATGTGG	A	ATAACATACTTACCTATGTGG	A	8182	TRUE	reported	233	TRUE	reported	233	8182
C	CATTTGGCAAAATTG	C	CATTTGGCAAAATTG	8182	TRUE	repor	233	TRUE	repor	233	8182

T	C	T	C	8182	TRUE	reported	233	TRUE	reported	233	8182
T	C	T	C	8182	TRUE	reported	233	TRUE	reported	233	8182
GGGCAGA	G	GGGCAGA	G	8182	TRUE	reported	233	TRUE	reported	233	8182
T	TAC	T	TAC	8182	TRUE	reported	233	TRUE	reported	233	8182

CAG	C	CAG	C	8182	TRUE	reported	233	TRUE	reported	233	8182
G	T	G	T	8182	TRUE	reported	233	TRUE	reported	233	8182
T	C	T	C	8182	TRUE	reported	233	TRUE	reported	233	8182
G	GA	G	GA	8182	TRUE	reported	233	TRUE	reported	233	8182

						d			d		
T	C	T	C	8182	TRUE	reported	233	TRUE	reported	233	8182
A	G	A	G	8182	TRUE	reported	233	TRUE	reported	233	8182
C	CAG	C	CAG	8182	TRUE	reported	233	TRUE	reported	233	8182
T	C	T	C	8182	TRUE	reported	233	TRUE	reported	233	8182

A	AATTTT	A	AATTTT	8182	TRUE	reported	233	TRUE	reported	233	8182
A	C	A	C	8182	TRUE	reported	233	TRUE	reported	233	8182
T	TGGA	T	TGGA	8182	TRUE	reported	233	TRUE	reported	233	8182
G	C	G	C	8182	TRUE	reported	233	TRUE	reported	233	8182
T	C	T	C	8	T	r	2	T	r	2	8

				182	RUE	eported	33	RUE	eported	33	182
A	G	A	G	8182	TRUE	reported	233	TRUE	reported	233	8182
A	G	A	G	8182	TRUE	reported	233	TRUE	reported	233	8182
T	TAACA	T	TAACA	8182	TRUE	reported	233	TRUE	reported	233	8182
C	T	C	T	8	T	r	2	T	r	2	8

				182	RUE	eported	33	RUE	eported	33	182
A	G	A	G	8182	TRUE	reported	233	TRUE	reported	233	8182
T	C	T	C	8182	TRUE	reported	233	TRUE	reported	233	8182
A	ATGTT	A	ATGTT	8182	TRUE	reported	233	TRUE	reported	233	8182
T	TTCTG	T	TTCTG	81	TR	re	23	TR	re	23	81

				82	UE	ported	3	UE	ported	3	82
A	G	A	G	8182	TRUE	reported	233	TRUE	reported	233	8182
A	G	A	G	8182	TRUE	reported	233	TRUE	reported	233	8182
G	A	G	A	8182	TRUE	reported	233	TRUE	reported	233	8182
C	CTTG	C	CTTG	818	TRU	rep	233	TRU	rep	233	818

				2	E	orted		E	orted		2
G	GTTAGTT	G	GTTAGTT	8182	TRUE	reported	233	TRUE	reported	233	8182
T	G	T	G	8182	TRUE	reported	233	TRUE	reported	233	8182
T	C	T	C	8182	TRUE	reported	233	TRUE	reported	233	8182
T	C	T	C	8182	TRUE	repo	233	TRUE	repo	233	8182

G	T	G	T	8182	TRUE	reported	233	TRUE	reported	233	8182
A	G	A	G	8182	TRUE	reported	233	TRUE	reported	233	8182
G	A	G	A	8182	TRUE	reported	233	TRUE	reported	233	8182
A	AT	A	AT	8182	TRUE	repo	233	TRUE	repo	233	8182

						rted			rted		
CA	C	CA	C	8182	TRUE	reported	233	TRUE	reported	233	8182
TCAAG	T	TCAAG	T	8182	TRUE	reported	233	TRUE	reported	233	8182
G	GGGGA	G	GGGGA	8182	TRUE	reported	233	TRUE	reported	233	8182
A	G	A	G	8182	TRUE	repor	233	TRUE	repor	233	8182

						ted			ted		
G	C	G	C	8182	TRUE	reported	233	TRUE	reported	233	8182
C	G	C	G	8182	TRUE	reported	233	TRUE	reported	233	8182
TCCA	T	TCCA	T	8182	TRUE	reported	233	TRUE	reported	233	8182
T	G	T	G	8182	TRUE	repor	233	TRUE	repor	233	8182

CAG	C	CAG	C	8182	TRUE	reported	233	TRUE	reported	233	8182
G	GT	G	GT	8182	TRUE	reported	233	TRUE	reported	233	8182
AC	A	AC	A	8182	TRUE	reported	233	TRUE	reported	233	8182
A	G	A	G	8182	TRUE	reported	233	TRUE	reported	233	8182

C	CCA	C	CCA	8182	TRUE	reported	233	TRUE	reported	233	8182
AG	A	AG	A	8182	TRUE	reported	233	TRUE	reported	233	8182
CT	C	CT	C	8182	TRUE	reported	233	TRUE	reported	233	8182
GCAA	G	GCAA	G	8182	TRUE	reported	233	TRUE	reported	233	8182

						d			d		
CA	C	CA	C	8182	TRUE	reported	233	TRUE	reported	233	8182
T	TC	T	TC	8182	TRUE	reported	233	TRUE	reported	233	8182
A	AT	A	AT	8182	TRUE	reported	233	TRUE	reported	233	8182
G	GT	G	GT	8182	TRUE	reported	233	TRUE	reported	233	8182

A	AG	A	AG	8182	TRUE	reported	233	TRUE	reported	233	8182
GCACTCGGCTCACT	G	GCACTCGGCTCACT	G	8182	TRUE	reported	233	TRUE	reported	233	8182
G	GC	G	GC	8182	TRUE	reported	233	TRUE	reported	233	8182
CTCT	C	CTCT	C	8182	TRUE	reported	233	TRUE	reported	233	8182

GC	G	GC	G	8182	TRUE	reported	233	TRUE	reported	233	8182
AC	A	AC	A	8182	TRUE	reported	233	TRUE	reported	233	8182
C	T	C	T	8182	TRUE	reported	233	TRUE	reported	233	8182
CGGGCACCCTCCAGTC	C	CGGGCACCCTCCAGTC	C	8182	TRUE	reported	233	TRUE	reported	233	8182

ACAGGCCAGAACAGGGGA		ACAGGCCAGAACAGGGGA								
G	A	G	A	8182	TRUE	reported	233	TRUE	reported	233	8182
A	G	A	G	8182	TRUE	reported	233	TRUE	reported	233	8182
A	ATTGAGG	A	ATTGAGG	8182	TRUE	reported	233	TRUE	reported	233	8182

T	C	T	C	8182	TRUE	reported	233	TRUE	reported	233	8182
CTT	C	CTT	C	8182	TRUE	reported	233	TRUE	reported	233	8182
G	GC	G	GC	8182	TRUE	reported	233	TRUE	reported	233	8182
A	AAT	A	AAT	8182	TRUE	reported	233	TRUE	reported	233	8182

G	GCT	G	GCT	8182	TRUE	reported	233	TRUE	reported	233	8182
A	AAATT	A	AAATT	8182	TRUE	reported	233	TRUE	reported	233	8182
G	A	G	A	8182	TRUE	reported	233	TRUE	reported	233	8182
T	C	T	C	8182	TRUE	reported	233	TRUE	reported	233	8182

C	CT	C	CT	8182	TRUE	reported	233	TRUE	reported	233	8182
C	CTTT	C	CTTT	8182	TRUE	reported	233	TRUE	reported	233	8182
T	TA	T	TA	8182	TRUE	reported	233	TRUE	reported	233	8182
G	C	G	C	8182	TRUE	reported	233	TRUE	reported	233	8182

T	TGCAGGC	T	TGCAGGC	8182	TRUE	reported	233	TRUE	reported	233	8182
C	CA	C	CA	8182	TRUE	reported	233	TRUE	reported	233	8182
T	TTATATGAGAATATTCATAGTTACTTACGCACAGTAGCCAACAAGTAAACCTGTCTCCCGTCAGA	T	TTATATGAGAATATTCATAGTTACTTACGCACAGTAGCCAACAAGTAAACCTGTCTCCCGTCAGA	8182	TRUE	reported	233	TRUE	reported	233	8182
C	A	C	A	818	TRU	rep	233	TRU	rep	233	818

				2	E	orted		E	orted	2
T	C	T	C	8182	TRUE	reported	233	TRUE	reported	233	8182
G	A	G	A	8182	TRUE	reported	233	TRUE	reported	233	8182
A	G	A	G	8182	TRUE	reported	233	TRUE	reported	233	8182
A	G	A	G	818	TRU	rep	233	TRU	rep	233	818

				2	E	orted		E	orted		2
A	T	A	T	8182	TRUE	reported	233	TRUE	reported	233	8182
G	T	G	T	8182	TRUE	reported	233	TRUE	reported	233	8182
G	A	G	A	8182	TRUE	reported	233	TRUE	reported	233	8182
G	A	G	A	818	TRU	rep	233	TRU	rep	233	818

				2	E	orted		E	orted		2
ATTTTTT	A	ATTTTTT	A	8182	TRUE	reported	233	TRUE	reported	233	8182
C	G	C	G	8182	TRUE	reported	233	TRUE	reported	233	8182
A	G	A	G	8182	TRUE	reported	233	TRUE	reported	233	8182
A	C	A	C	818	TRU	rep	233	TRU	rep	233	818

C	T	C	T	2 8182	E TRUE	ortedreported	233	E TRUE	ortedreported	233	2 8182

Table S7. Summary information on GWAS variants for neuroblastoma used in the MR analyses.

snp	rsid	hg19_coordinates	hg38_coordinates	a1	a2	trait	efo	s
rs6668141	rs6668141	chr1:21765268	chr1:21438775	C	G	Hypertrophic disorders of skin	EFO_0000701	N
rs4399126	rs4399126	chr1:21779090	chr1:21452597	C	T	Cause of death: appendix	-	N
rs4399126	rs4399126	chr1:21779090	chr1:21452597	C	T	Hypertrophic disorders of skin	EFO_0000701	N
rs2316505	rs2316505	chr1:21793958	chr1:21467465	T	C	Alkaline phosphatase	EFO_0004533	P
rs10632199	rs10632199	chr1:21796517	chr1:21470024	-	CT	Alkaline phosphatase	EFO_0004533	P
rs114517220	rs114517220	chr6:32245030	chr6:32277253	C	T	Rheumatoid arthritis	EFO_0000685	C
rs114517220	rs114517220	chr6:32245030	chr6:32277253	C	T	Rheumatoid arthritis	EFO_0000685	C
rs114365500	rs114365500	chr6:32318384	chr6:32350607	A	G	Body mass index	EFO_0004340	A
rs114365500	rs114365500	chr6:32318384	chr6:32350607	A	G	Rheumatoid arthritis	EFO_0000685	C
rs114365500	rs114365500	chr6:32318384	chr6:32350607	A	G	Rheumatoid arthritis	EFO_0000685	C
rs76254525	rs76254525	chr10:74791926	chr10:73032168	C	T	Cause of death: dilated cardiomyopathy	EFO_0000407	N
rs79452543	rs79452543	chr10:74792538	chr10:73032780	A	G	Cause of death: dilated cardiomyopathy	EFO_0000407	N
rs78746248	rs78746248	chr10:74793062	chr10:73033304	A	G	Cause of death: dilated cardiomyopathy	EFO_0000407	N
rs76289429	rs76289429	chr10:74793990	chr10:73034232	G	T	Cause of death: dilated cardiomyopathy	EFO_0000407	N

rs111395535	rs111395535	chr10:74795882	chr10:73036124	C	T	Cause of death: dilated cardiomyopathy	EFO_0000407	N
rs145642652	rs145642652	chr10:74796403	chr10:73036645	G	T	Cause of death: dilated cardiomyopathy	EFO_0000407	N
rs57670381	rs57670381	chr10:74799266	chr10:73039508	C	T	Cause of death: dilated cardiomyopathy	EFO_0000407	N
rs111693847	rs111693847	chr10:74800223	chr10:73040465	C	T	Cause of death: dilated cardiomyopathy	EFO_0000407	N
rs145170639	rs145170639	chr10:74800302	chr10:73040544	C	T	Cause of death: dilated cardiomyopathy	EFO_0000407	N
rs113577689	rs113577689	chr10:74800969	chr10:73041211	C	G	Cause of death: dilated cardiomyopathy	EFO_0000407	N
rs112849861	rs112849861	chr10:74801119	chr10:73041361	A	G	Cause of death: dilated cardiomyopathy	EFO_0000407	N
rs80326975	rs80326975	chr10:74801473	chr10:73041715	C	T	Cause of death: dilated cardiomyopathy	EFO_0000407	N
rs112185101	rs112185101	chr10:74801805	chr10:73042047	A	C	Cause of death: dilated cardiomyopathy	EFO_0000407	N
rs77006767	rs77006767	chr10:74802095	chr10:73042337	C	T	Cause of death: dilated cardiomyopathy	EFO_0000407	N
rs77832337	rs77832337	chr10:74802205	chr10:73042447	A	T	Cause of death: dilated cardiomyopathy	EFO_0000407	N
rs79531226	rs79531226	chr10:74802470	chr10:73042712	A	T	Cause of death: dilated cardiomyopathy	EFO_0000407	N
rs80234730	rs80234730	chr10:74804109	chr10:73044351	C	T	Cause of death: dilated cardiomyopathy	EFO_0000407	N
rs60857017	rs60857017	chr10:74804166	chr10:73044408	A	T	Cause of death: dilated cardiomyopathy	EFO_0000407	N
rs75797030	rs75797030	chr10:74805785	chr10:73046027	C	T	Cause of death: dilated cardiomyopathy	EFO_0000407	N
rs77287032	rs77287032	chr10:74807723	chr10:73047965	C	T	Cause of death: dilated cardiomyopathy	EFO_0000407	N
rs6480660	rs6480660	chr10:74809659	chr10:73049901	C	T	Cause of death: dilated cardiomyopathy	EFO_0000407	N
rs6480661	rs6480661	chr10:74809780	chr10:73050022	C	T	Cause of death: dilated cardiomyopathy	EFO_0000407	N
rs6480662	rs6480662	chr10:74809827	chr10:73050069	A	G	Cause of death: dilated cardiomyopathy	EFO_0000407	N
rs142813831	rs142813831	chr10:74810319	chr10:73050561	A	G	Cause of death: dilated cardiomyopathy	EFO_0000407	N
rs147424579	rs147424579	chr10:74811316	chr10:73051558	A	G	Cause of death: dilated cardiomyopathy	EFO_0000407	N
rs113914470	rs113914470	chr10:74811399	chr10:73051641	C	T	Cause of death: dilated cardiomyopathy	EFO_0000407	N
rs77960319	rs77960319	chr10:74812179	chr10:73052421	A	G	Cause of death: dilated cardiomyopathy	EFO_0000407	N
rs74560165	rs74560165	chr10:74814636	chr10:73054878	C	T	Cause of death: dilated cardiomyopathy	EFO_0000407	N
rs74818016	rs74818016	chr10:74815300	chr10:73055542	A	G	Cause of death: dilated cardiomyopathy	EFO_0000407	N
rs111840841	rs111840841	chr10:74816645	chr10:73056887	C	T	Cause of death: dilated cardiomyopathy	EFO_0000407	N
rs6480663	rs6480663	chr10:74816769	chr10:73057011	C	G	Cause of death: dilated cardiomyopathy	EFO_0000407	N
rs112733441	rs112733441	chr10:74817773	chr10:73058015	C	T	Cause of death: dilated cardiomyopathy	EFO_0000407	N
rs112489275	rs112489275	chr10:74817951	chr10:73058193	A	T	Cause of death: dilated cardiomyopathy	EFO_0000407	N
rs115866139	rs115866139	chr10:74818576	chr10:73058818	C	T	Cause of death: dilated cardiomyopathy	EFO_0000407	N
rs7095773	rs7095773	chr10:74819156	chr10:73059398	C	T	Cause of death: dilated cardiomyopathy	EFO_0000407	N
rs112388936	rs112388936	chr10:74823111	chr10:73063353	A	G	Cause of death: dilated cardiomyopathy	EFO_0000407	N
rs111393464	rs111393464	chr10:74827310	chr10:73067552	C	T	Cause of death: dilated cardiomyopathy	EFO_0000407	N

rs78579281	rs78579281	chr10:74827693	chr10:73067935	C	G	Cause of death: dilated cardiomyopathy	EFO_0000407	N
rs76800487	rs76800487	chr10:74835996	chr10:73076238	G	T	Cause of death: dilated cardiomyopathy	EFO_0000407	N
rs76591520	rs76591520	chr10:74837287	chr10:73077529	A	C	Cause of death: dilated cardiomyopathy	EFO_0000407	N
rs143364093	rs143364093	chr10:74839356	chr10:73079598	C	T	Cause of death: dilated cardiomyopathy	EFO_0000407	N
rs77155271	rs77155271	chr10:74842484	chr10:73082726	A	C	Cause of death: dilated cardiomyopathy	EFO_0000407	N
rs75233197	rs75233197	chr10:74843456	chr10:73083698	A	G	Cause of death: dilated cardiomyopathy	EFO_0000407	N
rs57908433	rs57908433	chr10:74843761	chr10:73084003	A	G	Cause of death: dilated cardiomyopathy	EFO_0000407	N
rs112484156	rs112484156	chr10:74847249	chr10:73087491	C	T	Cause of death: dilated cardiomyopathy	EFO_0000407	N
rs79957003	rs79957003	chr10:74847809	chr10:73088051	C	T	Cause of death: dilated cardiomyopathy	EFO_0000407	N
rs7072019	rs7072019	chr10:74849265	chr10:73089507	A	G	Cause of death: dilated cardiomyopathy	EFO_0000407	N
rs113764404	rs113764404	chr10:74850623	chr10:73090865	C	T	Cause of death: dilated cardiomyopathy	EFO_0000407	N
rs76269888	rs76269888	chr10:74851323	chr10:73091565	A	C	Cause of death: dilated cardiomyopathy	EFO_0000407	N
rs77961051	rs77961051	chr10:74851821	chr10:73092063	C	G	Cause of death: dilated cardiomyopathy	EFO_0000407	N
rs79900070	rs79900070	chr10:74851884	chr10:73092126	A	G	Cause of death: dilated cardiomyopathy	EFO_0000407	N
rs113949876	rs113949876	chr10:74854685	chr10:73094927	G	T	Cause of death: dilated cardiomyopathy	EFO_0000407	N
rs61639324	rs61639324	chr10:74855168	chr10:73095410	C	T	Cause of death: dilated cardiomyopathy	EFO_0000407	N
rs56705460	rs56705460	chr10:74856517	chr10:73096759	T	G	Cause of death: dilated cardiomyopathy	EFO_0000407	N
rs76151799	rs76151799	chr10:74856855	chr10:73097097	C	G	Cause of death: dilated cardiomyopathy	EFO_0000407	N
rs112580379	rs112580379	chr10:74858358	chr10:73098600	C	T	Cause of death: dilated cardiomyopathy	EFO_0000407	N
rs111348695	rs111348695	chr10:74858645	chr10:73098887	A	C	Cause of death: dilated cardiomyopathy	EFO_0000407	N
rs77631749	rs77631749	chr10:74859065	chr10:73099307	A	G	Cause of death: dilated cardiomyopathy	EFO_0000407	N
rs75943324	rs75943324	chr10:74859775	chr10:73100017	A	G	Cause of death: dilated cardiomyopathy	EFO_0000407	N
rs77289022	rs77289022	chr10:74863774	chr10:73104016	A	G	Cause of death: dilated cardiomyopathy	EFO_0000407	N
rs114800426	rs114800426	chr10:74868032	chr10:73108274	A	G	Cause of death: dilated cardiomyopathy	EFO_0000407	N
rs113756209	rs113756209	chr10:74869420	chr10:73109662	C	T	Cause of death: dilated cardiomyopathy	EFO_0000407	N
rs80084952	rs80084952	chr10:74870610	chr10:73110852	A	G	Cause of death: dilated cardiomyopathy	EFO_0000407	N
rs4498900	rs4498900	chr10:74874437	chr10:73114679	C	T	Cause of death: dilated cardiomyopathy	EFO_0000407	N
rs7899018	rs7899018	chr10:74874572	chr10:73114814	C	G	Cause of death: dilated cardiomyopathy	EFO_0000407	N
rs7077047	rs7077047	chr10:74880462	chr10:73120704	C	T	Cause of death: dilated cardiomyopathy	EFO_0000407	N
rs7911267	rs7911267	chr10:74882762	chr10:73123004	A	G	Cause of death: dilated cardiomyopathy	EFO_0000407	N
rs7095710	rs7095710	chr10:74884219	chr10:73124461	A	C	Cause of death: dilated cardiomyopathy	EFO_0000407	N
rs7098717	rs7098717	chr10:74887972	chr10:73128214	C	T	Cause of death: dilated cardiomyopathy	EFO_0000407	N
rs59909855	rs59909855	chr10:74888691	chr10:73128933	C	T	Cause of death: dilated cardiomyopathy	EFO_0000407	N

rs60631126	rs60631126	chr10:74891328	chr10:73131570	G	T	Cause of death: dilated cardiomyopathy	EFO_0000407	N
rs75096691	rs75096691	chr10:74891802	chr10:73132044	A	T	Cause of death: dilated cardiomyopathy	EFO_0000407	N
rs60938534	rs60938534	chr10:74896329	chr10:73136571	C	G	Cause of death: dilated cardiomyopathy	EFO_0000407	N
rs138123413	rs138123413	chr10:74898348	chr10:73138590	A	G	Cause of death: dilated cardiomyopathy	EFO_0000407	N
rs111524986	rs111524986	chr10:74900039	chr10:73140281	C	G	Cause of death: dilated cardiomyopathy	EFO_0000407	N
rs7912539	rs7912539	chr10:74900557	chr10:73140799	A	G	Cause of death: dilated cardiomyopathy	EFO_0000407	N
rs148985097	rs148985097	chr10:74901187	chr10:73141429	A	G	Cause of death: dilated cardiomyopathy	EFO_0000407	N
rs58690198	rs58690198	chr10:74901570	chr10:73141812	A	G	Cause of death: dilated cardiomyopathy	EFO_0000407	N
rs113353214	rs113353214	chr10:74902131	chr10:73142373	A	G	Cause of death: dilated cardiomyopathy	EFO_0000407	N
rs79050388	rs79050388	chr10:74907429	chr10:73147671	A	T	Cause of death: dilated cardiomyopathy	EFO_0000407	N
rs7914435	rs7914435	chr10:74913037	chr10:73153279	C	T	Cause of death: dilated cardiomyopathy	EFO_0000407	N
rs7915191	rs7915191	chr10:74913624	chr10:73153866	C	T	Cause of death: dilated cardiomyopathy	EFO_0000407	N
rs61081832	rs61081832	chr10:74915488	chr10:73155730	A	G	Cause of death: dilated cardiomyopathy	EFO_0000407	N
rs58021674	rs58021674	chr10:74917171	chr10:73157413	A	T	Cause of death: dilated cardiomyopathy	EFO_0000407	N
rs7905296	rs7905296	chr10:74918196	chr10:73158438	A	C	Cause of death: dilated cardiomyopathy	EFO_0000407	N
rs78306439	rs78306439	chr10:74918987	chr10:73159229	A	G	Cause of death: dilated cardiomyopathy	EFO_0000407	N
rs28709948	rs28709948	chr10:74919690	chr10:73159932	A	G	Cause of death: dilated cardiomyopathy	EFO_0000407	N
rs76305757	rs76305757	chr10:74925983	chr10:73166225	A	T	Cause of death: dilated cardiomyopathy	EFO_0000407	N
rs7077940	rs7077940	chr10:74926137	chr10:73166379	C	T	Cause of death: dilated cardiomyopathy	EFO_0000407	N
rs7078531	rs7078531	chr10:74926536	chr10:73166778	C	T	Cause of death: dilated cardiomyopathy	EFO_0000407	N
rs140243580	rs140243580	chr10:74930189	chr10:73170431	A	G	Cause of death: dilated cardiomyopathy	EFO_0000407	N
rs7916354	rs7916354	chr10:74931224	chr10:73171466	G	T	Cause of death: dilated cardiomyopathy	EFO_0000407	N
rs7905500	rs7905500	chr10:74931981	chr10:73172223	C	T	Cause of death: dilated cardiomyopathy	EFO_0000407	N
rs75029132	rs75029132	chr10:74934773	chr10:73175015	A	G	Cause of death: dilated cardiomyopathy	EFO_0000407	N
rs74955283	rs74955283	chr10:74934861	chr10:73175103	A	G	Cause of death: dilated cardiomyopathy	EFO_0000407	N
rs141487614	rs141487614	chr10:74938146	chr10:73178388	C	G	Cause of death: dilated cardiomyopathy	EFO_0000407	N
rs138649007	rs138649007	chr10:74939307	chr10:73179549	G	T	Cause of death: dilated cardiomyopathy	EFO_0000407	N
rs78537329	rs78537329	chr10:74942599	chr10:73182841	C	T	Cause of death: dilated cardiomyopathy	EFO_0000407	N
rs75029554	rs75029554	chr10:74944432	chr10:73184674	C	T	Cause of death: dilated cardiomyopathy	EFO_0000407	N
rs79950407	rs79950407	chr10:74944436	chr10:73184678	A	G	Cause of death: dilated cardiomyopathy	EFO_0000407	N
rs575575569	rs575575569	chr10:74944775	chr10:73185017	A	G	Cause of death: dilated cardiomyopathy	EFO_0000407	N
rs75847412	rs75847412	chr10:74959742	chr10:73199984	G	T	Cause of death: dilated cardiomyopathy	EFO_0000407	N
rs113054309	rs113054309	chr10:74964374	chr10:73204616	A	G	Cause of death: dilated cardiomyopathy	EFO_0000407	N

rs116742442	rs116742442	chr10:74964871	chr10:73205113	A	G	Cause of death: dilated cardiomyopathy	EFO_0000407	N
rs58936118	rs58936118	chr10:74967247	chr10:73207489	A	G	Cause of death: dilated cardiomyopathy	EFO_0000407	N
rs80074224	rs80074224	chr10:74969853	chr10:73210095	C	T	Cause of death: dilated cardiomyopathy	EFO_0000407	N
rs74423725	rs74423725	chr10:74977232	chr10:73217474	A	G	Cause of death: dilated cardiomyopathy	EFO_0000407	N
rs80257245	rs80257245	chr10:74979275	chr10:73219517	G	T	Cause of death: dilated cardiomyopathy	EFO_0000407	N
rs7904452	rs7904452	chr10:74981259	chr10:73221501	A	G	Cause of death: dilated cardiomyopathy	EFO_0000407	N
rs116806074	rs116806074	chr10:74982982	chr10:73223224	A	G	Cause of death: dilated cardiomyopathy	EFO_0000407	N
rs7899989	rs7899989	chr10:74987408	chr10:73227650	C	T	Cause of death: dilated cardiomyopathy	EFO_0000407	N
rs7086970	rs7086970	chr10:74991491	chr10:73231733	A	T	Cause of death: dilated cardiomyopathy	EFO_0000407	N
rs7082710	rs7082710	chr10:74994021	chr10:73234263	C	G	Cause of death: dilated cardiomyopathy	EFO_0000407	N
rs7083374	rs7083374	chr10:74994231	chr10:73234473	C	G	Cause of death: dilated cardiomyopathy	EFO_0000407	N
rs111472727	rs111472727	chr10:74999740	chr10:73239982	C	T	Cause of death: dilated cardiomyopathy	EFO_0000407	N
rs1047633	rs1047633	chr10:75001153	chr10:73241395	G	A	Cause of death: dilated cardiomyopathy	EFO_0000407	N
rs79102091	rs79102091	chr10:75004684	chr10:73244926	G	T	Cause of death: dilated cardiomyopathy	EFO_0000407	N
rs7075371	rs7075371	chr10:75010597	chr10:73250839	A	G	Cause of death: dilated cardiomyopathy	EFO_0000407	N
rs77239349	rs77239349	chr10:75011046	chr10:73251288	A	G	Cause of death: dilated cardiomyopathy	EFO_0000407	N
rs7905009	rs7905009	chr10:75011761	chr10:73252003	A	G	Cause of death: dilated cardiomyopathy	EFO_0000407	N
rs111672256	rs111672256	chr10:75012335	chr10:73252577	C	T	Cause of death: dilated cardiomyopathy	EFO_0000407	N
rs7090025	rs7090025	chr10:75013564	chr10:73253806	C	G	Cause of death: dilated cardiomyopathy	EFO_0000407	N
rs7921444	rs7921444	chr10:75014856	chr10:73255098	A	G	Cause of death: dilated cardiomyopathy	EFO_0000407	N
rs7922569	rs7922569	chr10:75015494	chr10:73255736	A	G	Cause of death: dilated cardiomyopathy	EFO_0000407	N
rs7074525	rs7074525	chr10:75017130	chr10:73257372	C	G	Cause of death: dilated cardiomyopathy	EFO_0000407	N
rs7075415	rs7075415	chr10:75017753	chr10:73257995	C	T	Cause of death: dilated cardiomyopathy	EFO_0000407	N
rs115303360	rs115303360	chr10:75019754	chr10:73259996	C	T	Cause of death: dilated cardiomyopathy	EFO_0000407	N
rs112198395	rs112198395	chr10:75022133	chr10:73262375	A	G	Cause of death: dilated cardiomyopathy	EFO_0000407	N
rs79648686	rs79648686	chr10:75022615	chr10:73262857	A	T	Cause of death: dilated cardiomyopathy	EFO_0000407	N
rs77281942	rs77281942	chr10:75024656	chr10:73264898	C	G	Cause of death: dilated cardiomyopathy	EFO_0000407	N
rs77782713	rs77782713	chr10:75027359	chr10:73267601	C	T	Cause of death: dilated cardiomyopathy	EFO_0000407	N
rs80349090	rs80349090	chr10:75027424	chr10:73267666	C	T	Cause of death: dilated cardiomyopathy	EFO_0000407	N
rs111332883	rs111332883	chr10:75034780	chr10:73275022	A	G	Cause of death: dilated cardiomyopathy	EFO_0000407	N
rs113395009	rs113395009	chr10:75037313	chr10:73277555	A	T	Cause of death: dilated cardiomyopathy	EFO_0000407	N
rs143703818	rs143703818	chr10:75037342	chr10:73277584	A	G	Cause of death: dilated cardiomyopathy	EFO_0000407	N
rs7899308	rs7899308	chr10:75037980	chr10:73278222	C	T	Cause of death: dilated cardiomyopathy	EFO_0000407	N

rs116071459	rs116071459	chr10:75038713	chr10:73278955	A	T	Cause of death: dilated cardiomyopathy	EFO_0000407	N
rs75243954	rs75243954	chr10:75038827	chr10:73279069	C	T	Cause of death: dilated cardiomyopathy	EFO_0000407	N
rs77220141	rs77220141	chr10:75040479	chr10:73280721	C	T	Cause of death: dilated cardiomyopathy	EFO_0000407	N
rs75452457	rs75452457	chr10:75046360	chr10:73286602	A	G	Cause of death: dilated cardiomyopathy	EFO_0000407	N
rs113394823	rs113394823	chr10:75046437	chr10:73286679	C	T	Cause of death: dilated cardiomyopathy	EFO_0000407	N
rs75925262	rs75925262	chr10:75047989	chr10:73288231	C	T	Cause of death: dilated cardiomyopathy	EFO_0000407	N
rs147165494	rs147165494	chr10:75049745	chr10:73289987	A	G	Cause of death: dilated cardiomyopathy	EFO_0000407	N
rs78707782	rs78707782	chr10:75052644	chr10:73292886	C	T	Cause of death: dilated cardiomyopathy	EFO_0000407	N
rs75006172	rs75006172	chr10:75052884	chr10:73293126	A	G	Cause of death: dilated cardiomyopathy	EFO_0000407	N
rs7088504	rs7088504	chr10:75053421	chr10:73293663	C	T	Cause of death: dilated cardiomyopathy	EFO_0000407	N
rs77425875	rs77425875	chr10:75054634	chr10:73294876	C	T	Cause of death: dilated cardiomyopathy	EFO_0000407	N
rs112008980	rs112008980	chr10:75054803	chr10:73295045	A	G	Cause of death: dilated cardiomyopathy	EFO_0000407	N
rs7069398	rs7069398	chr10:75056598	chr10:73296840	A	G	Cause of death: dilated cardiomyopathy	EFO_0000407	N
rs115323252	rs115323252	chr10:75060101	chr10:73300343	T	G	Cause of death: dilated cardiomyopathy	EFO_0000407	N
rs78835060	rs78835060	chr10:75060844	chr10:73301086	C	T	Cause of death: dilated cardiomyopathy	EFO_0000407	N
rs75384326	rs75384326	chr10:75062583	chr10:73302825	C	T	Cause of death: dilated cardiomyopathy	EFO_0000407	N
rs78671188	rs78671188	chr10:75064598	chr10:73304840	A	G	Cause of death: dilated cardiomyopathy	EFO_0000407	N
rs75922772	rs75922772	chr10:75065782	chr10:73306024	A	T	Cause of death: dilated cardiomyopathy	EFO_0000407	N
rs74714568	rs74714568	chr10:75065875	chr10:73306117	A	G	Cause of death: dilated cardiomyopathy	EFO_0000407	N
rs78334417	rs78334417	chr10:75071618	chr10:73311860	A	G	Cause of death: dilated cardiomyopathy	EFO_0000407	N
rs78343933	rs78343933	chr10:75072658	chr10:73312900	A	G	Cause of death: dilated cardiomyopathy	EFO_0000407	N
rs75649673	rs75649673	chr10:75074312	chr10:73314554	C	T	Cause of death: dilated cardiomyopathy	EFO_0000407	N
rs59699913	rs59699913	chr10:75077512	chr10:73317754	A	G	Cause of death: dilated cardiomyopathy	EFO_0000407	N
rs78423199	rs78423199	chr10:75078684	chr10:73318926	C	T	Cause of death: dilated cardiomyopathy	EFO_0000407	N
rs116010931	rs116010931	chr10:75080260	chr10:73320502	A	T	Cause of death: dilated cardiomyopathy	EFO_0000407	N
rs79170063	rs79170063	chr10:75089592	chr10:73329834	A	G	Cause of death: dilated cardiomyopathy	EFO_0000407	N
rs76566137	rs76566137	chr10:75091272	chr10:73331514	C	T	Cause of death: dilated cardiomyopathy	EFO_0000407	N
rs78570052	rs78570052	chr10:75091682	chr10:73331924	A	G	Cause of death: dilated cardiomyopathy	EFO_0000407	N
rs78745980	rs78745980	chr10:75098976	chr10:73339218	A	G	Cause of death: dilated cardiomyopathy	EFO_0000407	N
rs7094767	rs7094767	chr10:75102310	chr10:73342552	C	G	Cause of death: dilated cardiomyopathy	EFO_0000407	N
rs77084730	rs77084730	chr10:75102463	chr10:73342705	C	T	Cause of death: dilated cardiomyopathy	EFO_0000407	N
rs77657997	rs77657997	chr10:75104144	chr10:73344386	A	G	Cause of death: dilated cardiomyopathy	EFO_0000407	N
rs76797028	rs76797028	chr10:75104463	chr10:73344705	A	C	Cause of death: dilated cardiomyopathy	EFO_0000407	N

rs7898875	rs7898875	chr10:75108610	chr10:73348852	G	T	Cause of death: dilated cardiomyopathy	EFO_0000407	N
rs7893746	rs7893746	chr10:75108978	chr10:73349220	A	G	Cause of death: dilated cardiomyopathy	EFO_0000407	N
rs7903545	rs7903545	chr10:75111662	chr10:73351904	T	C	Cause of death: dilated cardiomyopathy	EFO_0000407	N
rs7077740	rs7077740	chr10:75118142	chr10:73358384	A	T	Cause of death: dilated cardiomyopathy	EFO_0000407	N
rs7077742	rs7077742	chr10:75118144	chr10:73358386	A	T	Cause of death: dilated cardiomyopathy	EFO_0000407	N
rs7078699	rs7078699	chr10:75118586	chr10:73358828	C	G	Cause of death: dilated cardiomyopathy	EFO_0000407	N
rs76205503	rs76205503	chr10:75118608	chr10:73358850	A	G	Cause of death: dilated cardiomyopathy	EFO_0000407	N
rs112073310	rs112073310	chr10:75119501	chr10:73359743	C	T	Cause of death: dilated cardiomyopathy	EFO_0000407	N
rs78274245	rs78274245	chr10:75119724	chr10:73359966	A	G	Cause of death: dilated cardiomyopathy	EFO_0000407	N
rs80294267	rs80294267	chr10:75119834	chr10:73360076	A	G	Cause of death: dilated cardiomyopathy	EFO_0000407	N
rs112604300	rs112604300	chr10:75121121	chr10:73361363	A	G	Cause of death: dilated cardiomyopathy	EFO_0000407	N
rs76761719	rs76761719	chr10:75121273	chr10:73361515	A	C	Cause of death: dilated cardiomyopathy	EFO_0000407	N
rs77467405	rs77467405	chr10:75122155	chr10:73362397	C	T	Cause of death: dilated cardiomyopathy	EFO_0000407	N
rs75257711	rs75257711	chr10:75122531	chr10:73362773	A	C	Cause of death: dilated cardiomyopathy	EFO_0000407	N
rs75789409	rs75789409	chr10:75122641	chr10:73362883	C	G	Cause of death: dilated cardiomyopathy	EFO_0000407	N
rs59696781	rs59696781	chr10:75123227	chr10:73363469	C	G	Cause of death: dilated cardiomyopathy	EFO_0000407	N
rs150806890	rs150806890	chr10:75123357	chr10:73363599	G	T	Cause of death: dilated cardiomyopathy	EFO_0000407	N
rs111454434	rs111454434	chr10:75123539	chr10:73363781	C	T	Cause of death: dilated cardiomyopathy	EFO_0000407	N
rs80197565	rs80197565	chr10:75128401	chr10:73368643	C	T	Cause of death: dilated cardiomyopathy	EFO_0000407	N
rs114114575	rs114114575	chr10:75129825	chr10:73370067	G	T	Cause of death: dilated cardiomyopathy	EFO_0000407	N
rs114947764	rs114947764	chr10:75133002	chr10:73373244	A	G	Cause of death: dilated cardiomyopathy	EFO_0000407	N
rs112784087	rs112784087	chr10:75134623	chr10:73374865	C	G	Cause of death: dilated cardiomyopathy	EFO_0000407	N
rs76733665	rs76733665	chr10:75134907	chr10:73375149	C	G	Cause of death: dilated cardiomyopathy	EFO_0000407	N
rs112796161	rs112796161	chr10:75134984	chr10:73375226	C	T	Cause of death: dilated cardiomyopathy	EFO_0000407	N
rs138011900	rs138011900	chr10:75137094	chr10:73377336	A	G	Treatment with conjugated oestrogens	EFO_0007056	N
rs60778687	rs60778687	chr10:75143608	chr10:73383850	A	G	Cause of death: dilated cardiomyopathy	EFO_0000407	N
rs145141016	rs145141016	chr10:75147108	chr10:73387350	A	G	Cause of death: dilated cardiomyopathy	EFO_0000407	N
rs79138489	rs79138489	chr10:75149677	chr10:73389919	A	G	Cause of death: dilated cardiomyopathy	EFO_0000407	N
rs78272680	rs78272680	chr10:75154952	chr10:73395194	C	T	Cause of death: dilated cardiomyopathy	EFO_0000407	N
rs7096747	rs7096747	chr10:75155603	chr10:73395845	C	T	Cause of death: dilated cardiomyopathy	EFO_0000407	N
rs7097105	rs7097105	chr10:75155718	chr10:73395960	A	G	Cause of death: dilated cardiomyopathy	EFO_0000407	N
rs7089932	rs7089932	chr10:75159094	chr10:73399336	C	T	Cause of death: dilated cardiomyopathy	EFO_0000407	N
rs76347812	rs76347812	chr10:75160205	chr10:73400447	A	G	Cause of death: dilated cardiomyopathy	EFO_0000407	N

rs78504939	rs78504939	chr10:75163791	chr10:73404033	A	G	Cause of death: dilated cardiomyopathy	EFO_0000407	N
rs74364724	rs74364724	chr10:75164517	chr10:73404759	C	T	Cause of death: dilated cardiomyopathy	EFO_0000407	N
rs111301467	rs111301467	chr10:75164676	chr10:73404918	C	G	Cause of death: dilated cardiomyopathy	EFO_0000407	N
rs7080666	rs7080666	chr10:75164711	chr10:73404953	A	T	Cause of death: dilated cardiomyopathy	EFO_0000407	N
rs147398126	rs147398126	chr10:75165794	chr10:73406036	A	G	Cause of death: dilated cardiomyopathy	EFO_0000407	N
rs112624196	rs112624196	chr10:75165905	chr10:73406147	A	G	Cause of death: dilated cardiomyopathy	EFO_0000407	N
rs75378229	rs75378229	chr10:75166023	chr10:73406265	A	G	Cause of death: dilated cardiomyopathy	EFO_0000407	N
rs112093254	rs112093254	chr10:75166626	chr10:73406868	C	G	Cause of death: dilated cardiomyopathy	EFO_0000407	N
rs78666269	rs78666269	chr10:75166793	chr10:73407035	C	T	Cause of death: dilated cardiomyopathy	EFO_0000407	N
rs77561916	rs77561916	chr10:75167490	chr10:73407732	A	T	Cause of death: dilated cardiomyopathy	EFO_0000407	N
rs112968920	rs112968920	chr10:75169884	chr10:73410126	C	T	Cause of death: dilated cardiomyopathy	EFO_0000407	N
rs138092214	rs138092214	chr10:75170440	chr10:73410682	C	T	Cause of death: dilated cardiomyopathy	EFO_0000407	N
rs149522905	rs149522905	chr10:75170497	chr10:73410739	A	G	Cause of death: dilated cardiomyopathy	EFO_0000407	N
rs116523249	rs116523249	chr10:75170655	chr10:73410897	A	G	Cause of death: dilated cardiomyopathy	EFO_0000407	N
rs114362341	rs114362341	chr10:75171344	chr10:73411586	C	T	Cause of death: dilated cardiomyopathy	EFO_0000407	N
rs144032562	rs144032562	chr10:75171850	chr10:73412092	A	C	Cause of death: dilated cardiomyopathy	EFO_0000407	N
rs139876371	rs139876371	chr10:75172038	chr10:73412280	A	C	Cause of death: dilated cardiomyopathy	EFO_0000407	N
rs79724519	rs79724519	chr10:75173215	chr10:73413457	C	G	Cause of death: dilated cardiomyopathy	EFO_0000407	N
rs7921604	rs7921604	chr10:75173778	chr10:73414020	A	G	Cause of death: dilated cardiomyopathy	EFO_0000407	N
rs7893866	rs7893866	chr10:75173933	chr10:73414175	C	G	Cause of death: dilated cardiomyopathy	EFO_0000407	N
rs76569269	rs76569269	chr10:75174247	chr10:73414489	A	G	Cause of death: dilated cardiomyopathy	EFO_0000407	N
rs75978651	rs75978651	chr10:75174482	chr10:73414724	A	G	Cause of death: dilated cardiomyopathy	EFO_0000407	N
rs77046628	rs77046628	chr10:75174972	chr10:73415214	C	T	Cause of death: dilated cardiomyopathy	EFO_0000407	N
rs146282221	rs146282221	chr10:75175417	chr10:73415659	C	T	Cause of death: dilated cardiomyopathy	EFO_0000407	N
rs114296591	rs114296591	chr10:75176862	chr10:73417104	A	G	Cause of death: dilated cardiomyopathy	EFO_0000407	N
rs150869608	rs150869608	chr10:75176988	chr10:73417230	C	T	Cause of death: dilated cardiomyopathy	EFO_0000407	N
rs74881713	rs74881713	chr10:75178478	chr10:73418720	C	T	Cause of death: dilated cardiomyopathy	EFO_0000407	N
rs76058310	rs76058310	chr10:75178574	chr10:73418816	A	C	Cause of death: dilated cardiomyopathy	EFO_0000407	N
rs79140299	rs79140299	chr10:75178661	chr10:73418903	A	G	Cause of death: dilated cardiomyopathy	EFO_0000407	N
rs111501682	rs111501682	chr10:75180003	chr10:73420245	C	T	Cause of death: dilated cardiomyopathy	EFO_0000407	N
rs77434482	rs77434482	chr10:75180155	chr10:73420397	A	G	Cause of death: dilated cardiomyopathy	EFO_0000407	N
rs7897404	rs7897404	chr10:75180196	chr10:73420438	A	G	Cause of death: dilated cardiomyopathy	EFO_0000407	N
rs147301841	rs147301841	chr10:75182074	chr10:73422316	C	T	Cause of death: dilated cardiomyopathy	EFO_0000407	N

rs113388185	rs113388185	chr10:75182355	chr10:73422597	A	G	Cause of death: dilated cardiomyopathy	EFO_0000407	N
rs75397737	rs75397737	chr10:75183717	chr10:73423959	A	G	Cause of death: dilated cardiomyopathy	EFO_0000407	N
rs141081035	rs141081035	chr10:75183901	chr10:73424143	C	T	Cause of death: dilated cardiomyopathy	EFO_0000407	N
rs113568690	rs113568690	chr10:75184082	chr10:73424324	C	T	Cause of death: dilated cardiomyopathy	EFO_0000407	N
rs61738877	rs61738877	chr10:75184323	chr10:73424565	A	G	Cause of death: dilated cardiomyopathy	EFO_0000407	N
rs61753915	rs61753915	chr10:75184463	chr10:73424705	A	C	Cause of death: dilated cardiomyopathy	EFO_0000407	N
rs7918817	rs7918817	chr10:75184843	chr10:73425085	A	T	Cause of death: dilated cardiomyopathy	EFO_0000407	N
rs61740737	rs61740737	chr10:75185810	chr10:73426052	A	G	Cause of death: dilated cardiomyopathy	EFO_0000407	N
rs112623518	rs112623518	chr10:75186987	chr10:73427229	C	G	Cause of death: dilated cardiomyopathy	EFO_0000407	N
rs7901375	rs7901375	chr10:75188304	chr10:73428546	A	G	Cause of death: dilated cardiomyopathy	EFO_0000407	N
rs7901811	rs7901811	chr10:75188393	chr10:73428635	A	G	Cause of death: dilated cardiomyopathy	EFO_0000407	N
rs7901820	rs7901820	chr10:75188416	chr10:73428658	G	T	Cause of death: dilated cardiomyopathy	EFO_0000407	N
rs116060245	rs116060245	chr10:75188583	chr10:73428825	G	T	Cause of death: dilated cardiomyopathy	EFO_0000407	N
rs57004021	rs57004021	chr10:75188796	chr10:73429038	C	G	Cause of death: dilated cardiomyopathy	EFO_0000407	N
rs58520739	rs58520739	chr10:75189045	chr10:73429287	C	T	Cause of death: dilated cardiomyopathy	EFO_0000407	N
rs79313251	rs79313251	chr10:75189945	chr10:73430187	C	T	Cause of death: dilated cardiomyopathy	EFO_0000407	N
rs75912927	rs75912927	chr10:75190376	chr10:73430618	A	G	Cause of death: dilated cardiomyopathy	EFO_0000407	N
rs74705804	rs74705804	chr10:75190452	chr10:73430694	C	T	Cause of death: dilated cardiomyopathy	EFO_0000407	N
rs60841809	rs60841809	chr10:75190562	chr10:73430804	A	C	Cause of death: dilated cardiomyopathy	EFO_0000407	N
rs59732104	rs59732104	chr10:75190599	chr10:73430841	A	G	Cause of death: dilated cardiomyopathy	EFO_0000407	N
rs57360214	rs57360214	chr10:75191092	chr10:73431334	C	T	Cause of death: dilated cardiomyopathy	EFO_0000407	N
rs77431511	rs77431511	chr10:75191430	chr10:73431672	C	T	Cause of death: dilated cardiomyopathy	EFO_0000407	N
rs76496523	rs76496523	chr10:75192181	chr10:73432423	C	G	Cause of death: dilated cardiomyopathy	EFO_0000407	N
rs56721165	rs56721165	chr10:75192662	chr10:73432904	A	G	Cause of death: dilated cardiomyopathy	EFO_0000407	N
rs75347554	rs75347554	chr10:75195164	chr10:73435406	A	G	Cause of death: dilated cardiomyopathy	EFO_0000407	N
rs113779478	rs113779478	chr10:75196318	chr10:73436560	C	T	Cause of death: dilated cardiomyopathy	EFO_0000407	N
rs113303275	rs113303275	chr10:75198334	chr10:73438576	C	T	Cause of death: dilated cardiomyopathy	EFO_0000407	N
rs74983485	rs74983485	chr10:75198342	chr10:73438584	A	C	Cause of death: dilated cardiomyopathy	EFO_0000407	N
rs78686744	rs78686744	chr10:75198642	chr10:73438884	A	G	Cause of death: dilated cardiomyopathy	EFO_0000407	N
rs75396753	rs75396753	chr10:75199042	chr10:73439284	G	T	Cause of death: dilated cardiomyopathy	EFO_0000407	N
rs112918082	rs112918082	chr10:75199362	chr10:73439604	C	T	Cause of death: dilated cardiomyopathy	EFO_0000407	N
rs77056132	rs77056132	chr10:75200182	chr10:73440424	C	G	Cause of death: dilated cardiomyopathy	EFO_0000407	N
rs77904169	rs77904169	chr10:75200802	chr10:73441044	G	T	Cause of death: dilated cardiomyopathy	EFO_0000407	N

rs59576105	rs59576105	chr10:75202248	chr10:73442490	A	T	Cause of death: dilated cardiomyopathy	EFO_0000407	
rs113719503	rs113719503	chr10:75202899	chr10:73443141	C	T	Cause of death: dilated cardiomyopathy	EFO_0000407	
rs113277317	rs113277317	chr10:75202911	chr10:73443153	C	T	Cause of death: dilated cardiomyopathy	EFO_0000407	
rs78200904	rs78200904	chr10:75203480	chr10:73443722	C	T	Cause of death: dilated cardiomyopathy	EFO_0000407	
rs4746135	rs4746135	chr10:75203506	chr10:73443748	G	T	Cause of death: dilated cardiomyopathy	EFO_0000407	
rs77321553	rs77321553	chr10:75204785	chr10:73445027	A	G	Cause of death: dilated cardiomyopathy	EFO_0000407	
rs78017725	rs78017725	chr10:75204882	chr10:73445124	A	T	Cause of death: dilated cardiomyopathy	EFO_0000407	
rs80125594	rs80125594	chr10:75204917	chr10:73445159	A	T	Cause of death: dilated cardiomyopathy	EFO_0000407	
rs112851220	rs112851220	chr10:75205263	chr10:73445505	A	G	Cause of death: dilated cardiomyopathy	EFO_0000407	
rs113095918	rs113095918	chr10:75205441	chr10:73445683	C	T	Cause of death: dilated cardiomyopathy	EFO_0000407	
rs3729975	rs3729975	chr10:75206378	chr10:73446620	T	C	Cause of death: dilated cardiomyopathy	EFO_0000407	
rs78406136	rs78406136	chr10:75207066	chr10:73447308	A	G	Cause of death: dilated cardiomyopathy	EFO_0000407	
rs76252158	rs76252158	chr10:75207072	chr10:73447314	A	G	Cause of death: dilated cardiomyopathy	EFO_0000407	
rs75077976	rs75077976	chr10:75208369	chr10:73448611	A	G	Cause of death: dilated cardiomyopathy	EFO_0000407	
rs112441825	rs112441825	chr10:75208503	chr10:73448745	A	G	Cause of death: dilated cardiomyopathy	EFO_0000407	
rs76502106	rs76502106	chr10:75208778	chr10:73449020	A	T	Cause of death: dilated cardiomyopathy	EFO_0000407	
rs115717094	rs115717094	chr10:75209671	chr10:73449913	A	T	Cause of death: dilated cardiomyopathy	EFO_0000407	
rs75505947	rs75505947	chr10:75209918	chr10:73450160	C	T	Cause of death: dilated cardiomyopathy	EFO_0000407	
rs76166331	rs76166331	chr10:75210351	chr10:73450593	A	C	Cause of death: dilated cardiomyopathy	EFO_0000407	
rs75396108	rs75396108	chr10:75210528	chr10:73450770	C	G	Cause of death: dilated cardiomyopathy	EFO_0000407	
rs113855839	rs113855839	chr10:75210728	chr10:73450970	C	T	Cause of death: dilated cardiomyopathy	EFO_0000407	
rs74403582	rs74403582	chr10:75210916	chr10:73451158	A	G	Cause of death: dilated cardiomyopathy	EFO_0000407	
rs114672768	rs114672768	chr10:75211048	chr10:73451290	C	T	Cause of death: dilated cardiomyopathy	EFO_0000407	
rs144894770	rs144894770	chr10:75211756	chr10:73451998	A	G	Cause of death: dilated cardiomyopathy	EFO_0000407	
rs113007191	rs113007191	chr10:75212225	chr10:73452467	A	G	Cause of death: dilated cardiomyopathy	EFO_0000407	
rs77903508	rs77903508	chr10:75212992	chr10:73453234	A	G	Cause of death: dilated cardiomyopathy	EFO_0000407	
rs76900864	rs76900864	chr10:75213441	chr10:73453683	A	C	Cause of death: dilated cardiomyopathy	EFO_0000407	
rs138826640	rs138826640	chr10:75214774	chr10:73455016	A	G	Cause of death: dilated cardiomyopathy	EFO_0000407	
rs114653980	rs114653980	chr10:75214787	chr10:73455029	C	T	Cause of death: dilated cardiomyopathy	EFO_0000407	
rs142833269	rs142833269	chr10:75214884	chr10:73455126	A	G	Cause of death: dilated cardiomyopathy	EFO_0000407	
rs78468448	rs78468448	chr10:75215175	chr10:73455417	C	T	Cause of death: dilated cardiomyopathy	EFO_0000407	
rs186876195	rs186876195	chr10:75215579	chr10:73455821	C	T	Cause of death: dilated cardiomyopathy	EFO_0000407	
rs77020387	rs77020387	chr10:75216066	chr10:73456308	C	T	Cause of death: dilated cardiomyopathy	EFO_0000407	

rs76633021	rs76633021	chr10:75216580	chr10:73456822	C	T	Cause of death: dilated cardiomyopathy	EFO_0000407	N
rs111388313	rs111388313	chr10:75217198	chr10:73457440	G	T	Cause of death: dilated cardiomyopathy	EFO_0000407	N
rs112361154	rs112361154	chr10:75217831	chr10:73458073	A	G	Cause of death: dilated cardiomyopathy	EFO_0000407	N
rs141594874	rs141594874	chr10:75218042	chr10:73458284	A	G	Cause of death: dilated cardiomyopathy	EFO_0000407	N
rs138356768	rs138356768	chr10:75218619	chr10:73458861	A	G	Cause of death: dilated cardiomyopathy	EFO_0000407	N
rs112604659	rs112604659	chr10:75218874	chr10:73459116	C	G	Cause of death: dilated cardiomyopathy	EFO_0000407	N
rs111956357	rs111956357	chr10:75220173	chr10:73460415	A	G	Cause of death: dilated cardiomyopathy	EFO_0000407	N
rs74771368	rs74771368	chr10:75220602	chr10:73460844	C	T	Cause of death: dilated cardiomyopathy	EFO_0000407	N
rs78349575	rs78349575	chr10:75220934	chr10:73461176	A	G	Cause of death: dilated cardiomyopathy	EFO_0000407	N
rs112964152	rs112964152	chr10:75221776	chr10:73462018	A	G	Cause of death: dilated cardiomyopathy	EFO_0000407	N
rs111539557	rs111539557	chr10:75222020	chr10:73462262	A	G	Cause of death: dilated cardiomyopathy	EFO_0000407	N
rs74496369	rs74496369	chr10:75222178	chr10:73462420	C	G	Cause of death: dilated cardiomyopathy	EFO_0000407	N
rs140853896	rs140853896	chr10:75223569	chr10:73463811	A	C	Cause of death: dilated cardiomyopathy	EFO_0000407	N
rs141852060	rs141852060	chr10:75223921	chr10:73464163	C	G	Cause of death: dilated cardiomyopathy	EFO_0000407	N
rs75427218	rs75427218	chr10:75224275	chr10:73464517	A	C	Cause of death: dilated cardiomyopathy	EFO_0000407	N
rs75781614	rs75781614	chr10:75224408	chr10:73464650	C	T	Cause of death: dilated cardiomyopathy	EFO_0000407	N
rs111630021	rs111630021	chr10:75224418	chr10:73464660	A	G	Cause of death: dilated cardiomyopathy	EFO_0000407	N
rs113908285	rs113908285	chr10:75224452	chr10:73464694	A	G	Cause of death: dilated cardiomyopathy	EFO_0000407	N
rs148768323	rs148768323	chr10:75224519	chr10:73464761	A	G	Cause of death: dilated cardiomyopathy	EFO_0000407	N
rs79766708	rs79766708	chr10:75225056	chr10:73465298	C	T	Cause of death: dilated cardiomyopathy	EFO_0000407	N
rs79475057	rs79475057	chr10:75225157	chr10:73465399	C	T	Cause of death: dilated cardiomyopathy	EFO_0000407	N
rs74530652	rs74530652	chr10:75226487	chr10:73466729	A	G	Cause of death: dilated cardiomyopathy	EFO_0000407	N
rs78406409	rs78406409	chr10:75229902	chr10:73470144	G	T	Cause of death: dilated cardiomyopathy	EFO_0000407	N
rs111566196	rs111566196	chr10:75231703	chr10:73471945	C	T	Cause of death: dilated cardiomyopathy	EFO_0000407	N
rs147500562	rs147500562	chr10:75232117	chr10:73472359	C	G	Cause of death: dilated cardiomyopathy	EFO_0000407	N
rs75146250	rs75146250	chr10:75232456	chr10:73472698	A	T	Cause of death: dilated cardiomyopathy	EFO_0000407	N
rs79381027	rs79381027	chr10:75233015	chr10:73473257	A	G	Cause of death: dilated cardiomyopathy	EFO_0000407	N
rs144074194	rs144074194	chr10:75233855	chr10:73474097	C	T	Cause of death: dilated cardiomyopathy	EFO_0000407	N
rs112090663	rs112090663	chr10:75234302	chr10:73474544	C	T	Cause of death: dilated cardiomyopathy	EFO_0000407	N
rs75076986	rs75076986	chr10:75235258	chr10:73475500	A	C	Cause of death: dilated cardiomyopathy	EFO_0000407	N
rs75822125	rs75822125	chr10:75235267	chr10:73475509	C	T	Cause of death: dilated cardiomyopathy	EFO_0000407	N
rs80301560	rs80301560	chr10:75235415	chr10:73475657	A	G	Cause of death: dilated cardiomyopathy	EFO_0000407	N
rs146720969	rs146720969	chr10:75235761	chr10:73476003	A	C	Cause of death: dilated cardiomyopathy	EFO_0000407	N

rs75275023	rs75275023	chr10:75240655	chr10:73480897	A	T	Cause of death: dilated cardiomyopathy	EFO_0000407	N
rs144658925	rs144658925	chr10:75241075	chr10:73481317	C	T	Cause of death: dilated cardiomyopathy	EFO_0000407	N
rs7095384	rs7095384	chr10:75241771	chr10:73482013	C	T	Cause of death: dilated cardiomyopathy	EFO_0000407	N
rs139294319	rs139294319	chr10:75242407	chr10:73482649	C	T	Cause of death: dilated cardiomyopathy	EFO_0000407	N
rs144072719	rs144072719	chr10:75242482	chr10:73482724	C	T	Cause of death: dilated cardiomyopathy	EFO_0000407	N
rs115392996	rs115392996	chr10:75243604	chr10:73483846	C	T	Cause of death: dilated cardiomyopathy	EFO_0000407	N
rs148354270	rs148354270	chr10:75243788	chr10:73484030	C	T	Cause of death: dilated cardiomyopathy	EFO_0000407	N
rs111785076	rs111785076	chr10:75244032	chr10:73484274	C	T	Cause of death: dilated cardiomyopathy	EFO_0000407	N
rs61591525	rs61591525	chr10:75244190	chr10:73484432	A	G	Cause of death: dilated cardiomyopathy	EFO_0000407	N
rs138210746	rs138210746	chr10:75244576	chr10:73484818	A	G	Cause of death: dilated cardiomyopathy	EFO_0000407	N
rs144023719	rs144023719	chr10:75246140	chr10:73486382	C	T	Cause of death: dilated cardiomyopathy	EFO_0000407	N
rs75427050	rs75427050	chr10:75246446	chr10:73486688	A	C	Cause of death: dilated cardiomyopathy	EFO_0000407	N
rs112262224	rs112262224	chr10:75246795	chr10:73487037	G	T	Cause of death: dilated cardiomyopathy	EFO_0000407	N
rs112693040	rs112693040	chr10:75246981	chr10:73487223	C	T	Cause of death: dilated cardiomyopathy	EFO_0000407	N
rs147567720	rs147567720	chr10:75247169	chr10:73487411	A	G	Cause of death: dilated cardiomyopathy	EFO_0000407	N
rs55687545	rs55687545	chr10:75247200	chr10:73487442	A	G	Cause of death: dilated cardiomyopathy	EFO_0000407	N
rs145801684	rs145801684	chr10:75247728	chr10:73487970	A	G	Cause of death: dilated cardiomyopathy	EFO_0000407	N
rs80335969	rs80335969	chr10:75247873	chr10:73488115	A	C	Cause of death: dilated cardiomyopathy	EFO_0000407	N
rs77174945	rs77174945	chr10:75247915	chr10:73488157	C	T	Cause of death: dilated cardiomyopathy	EFO_0000407	N
rs7095032	rs7095032	chr10:75248773	chr10:73489015	C	T	Cause of death: dilated cardiomyopathy	EFO_0000407	N
rs7083462	rs7083462	chr10:75248841	chr10:73489083	C	T	Cause of death: dilated cardiomyopathy	EFO_0000407	N
rs7083472	rs7083472	chr10:75248857	chr10:73489099	C	T	Cause of death: dilated cardiomyopathy	EFO_0000407	N
rs7923276	rs7923276	chr10:75249611	chr10:73489853	C	T	Cause of death: dilated cardiomyopathy	EFO_0000407	N
rs7917183	rs7917183	chr10:75251319	chr10:73491561	C	T	Cause of death: dilated cardiomyopathy	EFO_0000407	N
rs145304189	rs145304189	chr10:75251585	chr10:73491827	C	T	Cause of death: dilated cardiomyopathy	EFO_0000407	N
rs147557628	rs147557628	chr10:75251623	chr10:73491865	A	G	Cause of death: dilated cardiomyopathy	EFO_0000407	N
rs76653502	rs76653502	chr10:75251954	chr10:73492196	C	T	Cause of death: dilated cardiomyopathy	EFO_0000407	N
rs75570063	rs75570063	chr10:75252572	chr10:73492814	A	T	Cause of death: dilated cardiomyopathy	EFO_0000407	N
rs116689576	rs116689576	chr10:75252888	chr10:73493130	A	G	Cause of death: dilated cardiomyopathy	EFO_0000407	N
rs151179749	rs151179749	chr10:75252911	chr10:73493153	A	G	Cause of death: dilated cardiomyopathy	EFO_0000407	N
rs140205392	rs140205392	chr10:75252959	chr10:73493201	A	G	Cause of death: dilated cardiomyopathy	EFO_0000407	N
rs116121205	rs116121205	chr10:75254241	chr10:73494483	A	C	Cause of death: dilated cardiomyopathy	EFO_0000407	N
rs77810085	rs77810085	chr10:75254572	chr10:73494814	A	T	Cause of death: dilated cardiomyopathy	EFO_0000407	N

rs75317215	rs75317215	chr10:75254755	chr10:73494997	A	T	Cause of death: dilated cardiomyopathy	EFO_0000407	N
rs77301495	rs77301495	chr10:75255000	chr10:73495242	A	G	Cause of death: dilated cardiomyopathy	EFO_0000407	N
rs75657581	rs75657581	chr10:75255903	chr10:73496145	C	G	Cause of death: dilated cardiomyopathy	EFO_0000407	N
rs7099640	rs7099640	chr10:75256424	chr10:73496666	A	C	Cause of death: dilated cardiomyopathy	EFO_0000407	N
rs7099753	rs7099753	chr10:75256436	chr10:73496678	C	T	Cause of death: dilated cardiomyopathy	EFO_0000407	N
rs7099917	rs7099917	chr10:75256601	chr10:73496843	A	C	Cause of death: dilated cardiomyopathy	EFO_0000407	N
rs78007930	rs78007930	chr10:75257503	chr10:73497745	A	G	Cause of death: dilated cardiomyopathy	EFO_0000407	N
rs79933870	rs79933870	chr10:75260169	chr10:73500411	G	T	Cause of death: dilated cardiomyopathy	EFO_0000407	N
rs112673115	rs112673115	chr10:75260702	chr10:73500944	A	G	Cause of death: dilated cardiomyopathy	EFO_0000407	N
rs75835042	rs75835042	chr10:75260878	chr10:73501120	C	T	Cause of death: dilated cardiomyopathy	EFO_0000407	N
rs7098409	rs7098409	chr10:75261465	chr10:73501707	C	T	Cause of death: dilated cardiomyopathy	EFO_0000407	N
rs144040942	rs144040942	chr10:75262293	chr10:73502535	A	G	Cause of death: dilated cardiomyopathy	EFO_0000407	N
rs76254255	rs76254255	chr10:75262974	chr10:73503216	C	T	Cause of death: dilated cardiomyopathy	EFO_0000407	N
rs7899100	rs7899100	chr10:75263560	chr10:73503802	A	G	Cause of death: dilated cardiomyopathy	EFO_0000407	N
rs74643004	rs74643004	chr10:75263910	chr10:73504152	C	T	Cause of death: dilated cardiomyopathy	EFO_0000407	N
rs7083082	rs7083082	chr10:75265014	chr10:73505256	C	T	Cause of death: dilated cardiomyopathy	EFO_0000407	N
rs7083344	rs7083344	chr10:75265136	chr10:73505378	C	T	Cause of death: dilated cardiomyopathy	EFO_0000407	N
rs112009425	rs112009425	chr10:75265444	chr10:73505686	C	T	Cause of death: dilated cardiomyopathy	EFO_0000407	N
rs145941040	rs145941040	chr10:75265583	chr10:73505825	A	G	Cause of death: dilated cardiomyopathy	EFO_0000407	N
rs7077046	rs7077046	chr10:75267241	chr10:73507483	A	T	Cause of death: dilated cardiomyopathy	EFO_0000407	N
rs7093203	rs7093203	chr10:75267386	chr10:73507628	C	T	Cause of death: dilated cardiomyopathy	EFO_0000407	N
rs7093077	rs7093077	chr10:75267387	chr10:73507629	A	G	Cause of death: dilated cardiomyopathy	EFO_0000407	N
rs113629803	rs113629803	chr10:75269053	chr10:73509295	A	T	Cause of death: dilated cardiomyopathy	EFO_0000407	N
rs150353006	rs150353006	chr10:75269243	chr10:73509485	C	T	Cause of death: dilated cardiomyopathy	EFO_0000407	N
rs115460504	rs115460504	chr10:75273866	chr10:73514108	C	T	Cause of death: dilated cardiomyopathy	EFO_0000407	N
rs112917068	rs112917068	chr10:75273967	chr10:73514209	A	G	Cause of death: dilated cardiomyopathy	EFO_0000407	N
rs111936984	rs111936984	chr10:75282138	chr10:73522380	A	C	Cause of death: dilated cardiomyopathy	EFO_0000407	N
rs76285505	rs76285505	chr10:75282215	chr10:73522457	C	T	Cause of death: dilated cardiomyopathy	EFO_0000407	N
rs7909213	rs7909213	chr10:75283229	chr10:73523471	C	T	Cause of death: dilated cardiomyopathy	EFO_0000407	N
rs77809782	rs77809782	chr10:75285504	chr10:73525746	C	T	Cause of death: dilated cardiomyopathy	EFO_0000407	N
rs113737310	rs113737310	chr10:75286823	chr10:73527065	A	T	Cause of death: dilated cardiomyopathy	EFO_0000407	N
rs112576154	rs112576154	chr10:75287241	chr10:73527483	C	T	Cause of death: dilated cardiomyopathy	EFO_0000407	N
rs58845345	rs58845345	chr10:75292925	chr10:73533167	A	G	Cause of death: dilated cardiomyopathy	EFO_0000407	N

rs112331245	rs112331245	chr10:75294235	chr10:73534477	A	G	Cause of death: dilated cardiomyopathy	EFO_0000407	N
rs115058361	rs115058361	chr10:75298757	chr10:73538999	C	T	Cause of death: dilated cardiomyopathy	EFO_0000407	N
rs76548618	rs76548618	chr10:75299428	chr10:73539670	A	G	Cause of death: dilated cardiomyopathy	EFO_0000407	N
rs78896305	rs78896305	chr10:75304001	chr10:73544243	A	C	Cause of death: dilated cardiomyopathy	EFO_0000407	N
rs16930693	rs16930693	chr10:75305130	chr10:73545372	A	G	Cause of death: dilated cardiomyopathy	EFO_0000407	N
rs112409879	rs112409879	chr10:75307736	chr10:73547978	C	T	Cause of death: dilated cardiomyopathy	EFO_0000407	N
rs114036273	rs114036273	chr10:75308638	chr10:73548880	A	T	Cause of death: dilated cardiomyopathy	EFO_0000407	N
rs138177089	rs138177089	chr10:75309785	chr10:73550027	T	G	Cause of death: dilated cardiomyopathy	EFO_0000407	N
rs150505066	rs150505066	chr10:75310757	chr10:73550999	A	G	Cause of death: emphysema, unspecified	-	N
rs7078133	rs7078133	chr10:75314969	chr10:73555211	C	T	Cause of death: dilated cardiomyopathy	EFO_0000407	N
rs7094096	rs7094096	chr10:75314992	chr10:73555234	C	T	Cause of death: dilated cardiomyopathy	EFO_0000407	N
rs7094792	rs7094792	chr10:75315494	chr10:73555736	C	T	Cause of death: dilated cardiomyopathy	EFO_0000407	N
rs7094804	rs7094804	chr10:75315518	chr10:73555760	C	G	Cause of death: dilated cardiomyopathy	EFO_0000407	N
rs7073223	rs7073223	chr10:75322888	chr10:73563130	G	T	Cause of death: dilated cardiomyopathy	EFO_0000407	N
rs112652126	rs112652126	chr10:75327940	chr10:73568182	A	G	Cause of death: dilated cardiomyopathy	EFO_0000407	N
rs75706553	rs75706553	chr10:75331502	chr10:73571744	C	T	Cause of death: dilated cardiomyopathy	EFO_0000407	N
rs150336481	rs150336481	chr10:75334905	chr10:73575147	A	C	Cause of death: dilated cardiomyopathy	EFO_0000407	N
rs75355799	rs75355799	chr10:75335461	chr10:73575703	G	T	Cause of death: dilated cardiomyopathy	EFO_0000407	N
rs7073477	rs7073477	chr10:75344174	chr10:73584416	C	T	Cause of death: dilated cardiomyopathy	EFO_0000407	N
rs16930723	rs16930723	chr10:75346238	chr10:73586480	A	G	Cause of death: dilated cardiomyopathy	EFO_0000407	N
rs7070431	rs7070431	chr10:75347621	chr10:73587863	A	G	Cause of death: dilated cardiomyopathy	EFO_0000407	N
rs7068180	rs7068180	chr10:75356895	chr10:73597137	A	T	Cause of death: dilated cardiomyopathy	EFO_0000407	N
rs16930750	rs16930750	chr10:75359458	chr10:73599700	A	G	Cause of death: dilated cardiomyopathy	EFO_0000407	N
rs115943423	rs115943423	chr10:75361610	chr10:73601852	G	T	Cause of death: dilated cardiomyopathy	EFO_0000407	N
rs141066747	rs141066747	chr10:75362197	chr10:73602439	A	G	Cause of death: dilated cardiomyopathy	EFO_0000407	N
rs143771925	rs143771925	chr10:75365721	chr10:73605963	A	G	Cause of death: dilated cardiomyopathy	EFO_0000407	N
rs57722556	rs57722556	chr10:75367267	chr10:73607509	A	G	Cause of death: dilated cardiomyopathy	EFO_0000407	N
rs145495565	rs145495565	chr10:75373062	chr10:73613304	A	G	Cause of death: dilated cardiomyopathy	EFO_0000407	N
rs114627615	rs114627615	chr10:75375818	chr10:73616060	C	T	Cause of death: dilated cardiomyopathy	EFO_0000407	N
rs7900010	rs7900010	chr10:75376186	chr10:73616428	A	G	Cause of death: dilated cardiomyopathy	EFO_0000407	N
rs149858468	rs149858468	chr10:75378416	chr10:73618658	A	G	Cause of death: dilated cardiomyopathy	EFO_0000407	N
rs180795833	rs180795833	chr10:75378526	chr10:73618768	C	T	Cause of death: dilated cardiomyopathy	EFO_0000407	N
rs144686295	rs144686295	chr10:75392825	chr10:73633067	C	T	Cause of death: dilated cardiomyopathy	EFO_0000407	N

rs77735908	rs77735908	chr10:74761225	chr10:73001467	A	C	Cause of death: without complications	-	N
rs77330556	rs77330556	chr12:132569521	chr12:132084976	C	G	Cause of death: pneumonia, unspecified	-	N
rs77330556	rs77330556	chr12:132569521	chr12:132084976	C	G	Treatment with rosiglitazone	EFO_0007056	N
rs61942864	rs61942864	chr12:132587204	chr12:132102659	C	T	Sitting height	EFO_0004339	N
rs143582876	rs143582876	chr19:9623052	chr19:9512376	A	G	Cause of death: other and unspecified intestinal obstruction	-	N
rs147429849	rs147429849	chr19:9665587	chr19:9554911	A	G	Cause of death: other and unspecified intestinal obstruction	-	N
rs6051080	rs6051080	chr20:25975674	chr20:25995038	A	G	Colorectal or endometrial cancer	EFO_0005842;EFO_0004230	C
rs139930072	rs139930072	chr20:33895204	chr20:35307401	C	T	Arm fat-free mass left	-	N
rs139930072	rs139930072	chr20:33895204	chr20:35307401	C	T	Arm predicted mass left	-	N
rs139930072	rs139930072	chr20:33895204	chr20:35307401	C	T	Arm predicted mass right	-	N
rs139930072	rs139930072	chr20:33895204	chr20:35307401	C	T	Basal metabolic rate	EFO_0007777	N
rs139930072	rs139930072	chr20:33895204	chr20:35307401	C	T	Height	EFO_0004339	N
rs139930072	rs139930072	chr20:33895204	chr20:35307401	C	T	Impedance of leg left	-	N
rs139930072	rs139930072	chr20:33895204	chr20:35307401	C	T	Leg fat-free mass right	-	N
rs139930072	rs139930072	chr20:33895204	chr20:35307401	C	T	Leg predicted mass right	-	N
rs139930072	rs139930072	chr20:33895204	chr20:35307401	C	T	Trunk fat-free mass	-	N
rs139930072	rs139930072	chr20:33895204	chr20:35307401	C	T	Trunk predicted mass	-	N
rs139930072	rs139930072	chr20:33895204	chr20:35307401	C	T	Whole body fat-free mass	-	N
rs139930072	rs139930072	chr20:33895204	chr20:35307401	C	T	Whole body water mass	-	N
rs118059606	rs118059606	chr20:33976183	chr20:35388380	C	T	Arm fat-free mass left	-	N
rs118059606	rs118059606	chr20:33976183	chr20:35388380	C	T	Arm fat-free mass right	-	N
rs118059606	rs118059606	chr20:33976183	chr20:35388380	C	T	Basal metabolic rate	EFO_0007777	N
rs118059606	rs118059606	chr20:33976183	chr20:35388380	C	T	Comparative height size at age 10	-	N
rs118059606	rs118059606	chr20:33976183	chr20:35388380	C	T	Height	EFO_0004339	N
rs118059606	rs118059606	chr20:33976183	chr20:35388380	C	T	Impedance of leg left	-	N
rs118059606	rs118059606	chr20:33976183	chr20:35388380	C	T	Impedance of whole body	-	N
rs118059606	rs118059606	chr20:33976183	chr20:35388380	C	T	Leg fat-free mass left	-	N
rs118059606	rs118059606	chr20:33976183	chr20:35388380	C	T	Leg fat-free mass right	-	N
rs118059606	rs118059606	chr20:33976183	chr20:35388380	C	T	Leg predicted mass left	-	N
rs118059606	rs118059606	chr20:33976183	chr20:35388380	C	T	Leg predicted mass right	-	N
rs118059606	rs118059606	chr20:33976183	chr20:35388380	C	T	Sitting height	EFO_0004339	N
rs118059606	rs118059606	chr20:33976183	chr20:35388380	C	T	Trunk fat-free mass	-	N
rs118059606	rs118059606	chr20:33976183	chr20:35388380	C	T	Trunk predicted mass	-	N

rs118059606	rs118059606	chr20:33976183	chr20:35388380	C	T	Whole body fat-free mass	-	N
rs118059606	rs118059606	chr20:33976183	chr20:35388380	C	T	Whole body water mass	-	N
rs12625762	rs12625762	chr20:34158587	chr20:35570657	A	G	Arm fat-free mass left	-	N
rs12625762	rs12625762	chr20:34158587	chr20:35570657	A	G	Arm fat-free mass right	-	N
rs12625762	rs12625762	chr20:34158587	chr20:35570657	A	G	Arm predicted mass left	-	N
rs12625762	rs12625762	chr20:34158587	chr20:35570657	A	G	Arm predicted mass right	-	N
rs12625762	rs12625762	chr20:34158587	chr20:35570657	A	G	Basal metabolic rate	EFO_0007777	N
rs12625762	rs12625762	chr20:34158587	chr20:35570657	A	G	Comparative height size at age 10	-	N
rs12625762	rs12625762	chr20:34158587	chr20:35570657	A	G	Height	EFO_0004339	N
rs12625762	rs12625762	chr20:34158587	chr20:35570657	A	G	Impedance of arm left	-	N
rs12625762	rs12625762	chr20:34158587	chr20:35570657	A	G	Impedance of arm right	-	N
rs12625762	rs12625762	chr20:34158587	chr20:35570657	A	G	Impedance of leg left	-	N
rs12625762	rs12625762	chr20:34158587	chr20:35570657	A	G	Impedance of leg right	-	N
rs12625762	rs12625762	chr20:34158587	chr20:35570657	A	G	Impedance of whole body	-	N
rs12625762	rs12625762	chr20:34158587	chr20:35570657	A	G	Leg fat-free mass right	-	N
rs12625762	rs12625762	chr20:34158587	chr20:35570657	A	G	Leg predicted mass left	-	N
rs12625762	rs12625762	chr20:34158587	chr20:35570657	A	G	Leg predicted mass right	-	N
rs12625762	rs12625762	chr20:34158587	chr20:35570657	A	G	Sitting height	EFO_0004339	N
rs12625762	rs12625762	chr20:34158587	chr20:35570657	A	G	Trunk fat-free mass	-	N
rs12625762	rs12625762	chr20:34158587	chr20:35570657	A	G	Trunk predicted mass	-	N
rs12625762	rs12625762	chr20:34158587	chr20:35570657	A	G	Weight	EFO_0004338	N
rs12625762	rs12625762	chr20:34158587	chr20:35570657	A	G	Whole body water mass	-	N
rs11554015	rs11554015	chr20:34240084	chr20:35652162	G	A	Arm fat-free mass left	-	N
rs11554015	rs11554015	chr20:34240084	chr20:35652162	G	A	Arm fat-free mass right	-	N
rs11554015	rs11554015	chr20:34240084	chr20:35652162	G	A	Arm predicted mass left	-	N
rs11554015	rs11554015	chr20:34240084	chr20:35652162	G	A	Arm predicted mass right	-	N
rs11554015	rs11554015	chr20:34240084	chr20:35652162	G	A	Comparative height size at age 10	-	N
rs11554015	rs11554015	chr20:34240084	chr20:35652162	G	A	Leg fat-free mass left	-	N
rs11554015	rs11554015	chr20:34240084	chr20:35652162	G	A	Leg predicted mass left	-	N
rs11554015	rs11554015	chr20:34240084	chr20:35652162	G	A	Leg predicted mass right	-	N
rs11554015	rs11554015	chr20:34240084	chr20:35652162	G	A	Sitting height	EFO_0004339	N
rs11554015	rs11554015	chr20:34240084	chr20:35652162	G	A	Trunk fat-free mass	-	N
rs11554015	rs11554015	chr20:34240084	chr20:35652162	G	A	Trunk predicted mass	-	N

rs11554015	rs11554015	chr20:34240084	chr20:35652162	G	A	Whole body fat-free mass	-	N
rs11554015	rs11554015	chr20:34240084	chr20:35652162	G	A	Whole body water mass	-	N
rs2378392	rs2378392	chr20:34307765	chr20:35719843	C	G	Arm fat-free mass right	-	N
rs2378392	rs2378392	chr20:34307765	chr20:35719843	C	G	Comparative height size at age 10	-	N
rs2378392	rs2378392	chr20:34307765	chr20:35719843	C	G	Hand grip strength right	EFO_0006941	N
rs2378392	rs2378392	chr20:34307765	chr20:35719843	C	G	Height	EFO_0004339	N
rs2378392	rs2378392	chr20:34307765	chr20:35719843	C	G	Hip circumference	EFO_0005093	N
rs2378392	rs2378392	chr20:34307765	chr20:35719843	C	G	Impedance of arm right	-	N
rs2378392	rs2378392	chr20:34307765	chr20:35719843	C	G	Impedance of leg right	-	N
rs2378392	rs2378392	chr20:34307765	chr20:35719843	C	G	Impedance of whole body	-	N
rs2378392	rs2378392	chr20:34307765	chr20:35719843	C	G	Leg fat-free mass left	-	N
rs2378392	rs2378392	chr20:34307765	chr20:35719843	C	G	Leg predicted mass left	-	N
rs2378392	rs2378392	chr20:34307765	chr20:35719843	C	G	Leg predicted mass right	-	N
rs2378392	rs2378392	chr20:34307765	chr20:35719843	C	G	Sitting height	EFO_0004339	N
rs2378392	rs2378392	chr20:34307765	chr20:35719843	C	G	Treatment with vascalpha 5mg m or r tablet	EFO_0007056	N
rs2378392	rs2378392	chr20:34307765	chr20:35719843	C	G	Trunk fat mass	EFO_0005409	N
rs2378392	rs2378392	chr20:34307765	chr20:35719843	C	G	Trunk fat-free mass	-	N
rs2378392	rs2378392	chr20:34307765	chr20:35719843	C	G	Trunk predicted mass	-	N
rs2378392	rs2378392	chr20:34307765	chr20:35719843	C	G	Waist circumference	EFO_0004342	N
rs2378392	rs2378392	chr20:34307765	chr20:35719843	C	G	Weight	EFO_0004338	N
rs2378392	rs2378392	chr20:34307765	chr20:35719843	C	G	Whole body fat-free mass	-	N
rs2378392	rs2378392	chr20:34307765	chr20:35719843	C	G	Whole body water mass	-	N

Table S8. MR estimates of the associations between immune cell traits and neuroblastoma risk after FDR correction.

Panel	Trait	method	nsnp	b	se	pval	lo_ci	up_ci	or	or_lci95	or_uci9
B cell	IgD- CD27- AC	Inverse variance weighted	28	-0.32952	0.031537	1.48E-25	-0.39133	-0.26771	0.719267	0.676154	0.765
B cell	IgD- CD38br %lymphocyte	Inverse variance weighted	29	0.301205	0.04192	6.71E-13	0.219043	0.383368	1.351487	1.244884	1.467
B cell	IgD- CD38dim %lymphocyte	Inverse variance weighted	31	-0.25145	0.03752	2.06E-11	-0.32499	-0.17791	0.777669	0.722531	0.837
Treg	CD39+ resting Treg AC	Inverse variance weighted	96	0.206317	0.016057	8.74E-38	0.174845	0.23779	1.229143	1.191062	1.268
Treg	CD39+ resting Treg % CD4 Treg	Inverse variance weighted	114	0.159559	0.019568	3.51E-16	0.121207	0.197912	1.172994	1.128858	1.218
Treg	CD39+ activated Treg AC	Inverse variance weighted	141	0.212986	0.015495	5.41E-43	0.182617	0.243356	1.237368	1.200354	1.275

Treg	CD25hi CD45RA+ CD4 not Treg AC	Inverse variance weighted	37	0.280308	0.02412	3.20E-31	0.233033	0.327582	1.323537	1.262423	1.387
Myeloid cell	CD33dim HLA DR- AC	Inverse variance weighted	26	0.243528	0.025475	1.18E-21	0.193596	0.293459	1.275742	1.213606	1.341
B cell	Transitional AC	Inverse variance weighted	41	0.253192	0.03743	1.34E-11	0.179829	0.326555	1.288131	1.197013	1.386
Monocyte	CD14- CD16+ monocyte AC	Inverse variance weighted	23	0.372778	0.027965	1.54E-40	0.317967	0.42759	1.451762	1.374331	1.533
Monocyte	CD14+ CD16- monocyte AC	Inverse variance weighted	31	0.323284	0.023365	1.53E-43	0.277489	0.369078	1.381657	1.319812	1.446
TBNK	CD8dim AC	Inverse variance weighted	33	0.381113	0.014186	5.52E-159	0.353309	0.408918	1.463913	1.423771	1.505
TBNK	CD8dim %T cell	Inverse variance weighted	36	0.290541	0.03726	6.31E-15	0.217511	0.363572	1.337151	1.242979	1.438
TBNK	CD8dim %leukocyte	Inverse variance weighted	31	0.374564	0.014148	1.86E-154	0.346834	0.402293	1.454357	1.414582	1.495
TBNK	HLA DR+ CD4+ %lymphocyte	Inverse variance weighted	25	-0.33777	0.027478	9.95E-35	-0.39162	-0.28391	0.713361	0.675958	0.752
TBNK	CD8br NKT %T cell	Inverse variance weighted	28	-0.42071	0.0308	1.78E-42	-0.48108	-0.36034	0.656582	0.618118	0.69
TBNK	CD8br NKT %lymphocyte	Inverse variance weighted	30	-0.42858	0.031336	1.40E-42	-0.48999	-0.36716	0.651436	0.61263	0.692
Treg	CD39+ CD4+ %CD4+	Inverse variance weighted	208	0.135621	0.020022	1.26E-11	0.096377	0.174866	1.145248	1.101175	1.191
Treg	CD39+ CD8br AC	Inverse variance weighted	144	0.141543	0.020631	6.85E-12	0.101107	0.181979	1.15205	1.106395	1.199
Treg	CD28+ CD45RA+ CD8br AC	Inverse variance weighted	40	-0.50317	0.026668	2.09E-79	-0.55543	-0.4509	0.604613	0.573823	0.637
B cell	BAFF-R on CD24+ CD27+	Inverse variance weighted	178	-0.17605	0.007995	1.85E-107	-0.19172	-0.16038	0.838577	0.825539	0.851
B cell	BAFF-R on IgD+ CD24+	Inverse variance weighted	182	-0.17135	0.009563	8.67E-72	-0.19009	-0.1526	0.842528	0.826882	0.858
B cell	BAFF-R on IgD+ CD24-	Inverse variance weighted	173	-0.20251	0.008327	1.20E-130	-0.21883	-0.18619	0.816679	0.803459	0.830
B cell	BAFF-R on IgD+ CD38- naive	Inverse variance weighted	123	-0.18879	0.007435	2.94E-142	-0.20337	-0.17422	0.827958	0.81598	0.840
B cell	BAFF-R on IgD+ CD38- unsw mem	Inverse variance weighted	115	-0.18311	0.006856	3.62E-157	-0.19655	-0.16968	0.832674	0.82156	0.843
B cell	BAFF-R on IgD+ CD38dim	Inverse variance weighted	167	-0.1967	0.008374	5.26E-122	-0.21311	-0.18028	0.821439	0.808067	0.835
B cell	BAFF-R on IgD- CD27-	Inverse variance weighted	162	-0.19425	0.007396	5.05E-152	-0.20874	-0.17975	0.823454	0.811602	0.835
B cell	BAFF-R on IgD- CD38-	Inverse variance weighted	176	-0.16627	0.008806	1.62E-79	-0.18353	-0.14901	0.846819	0.832328	0.861
B cell	BAFF-R on IgD- CD38br	Inverse variance weighted	39	-0.3797	0.02248	5.32E-64	-0.42376	-0.33564	0.684069	0.654582	0.714
B cell	BAFF-R on unsw mem	Inverse variance weighted	186	-0.17054	0.009502	4.91E-72	-0.18917	-0.15192	0.843206	0.827647	0.859
B cell	BAFF-R on IgD+	Inverse variance weighted	171	-0.19919	0.008017	2.76E-136	-0.2149	-0.18348	0.819395	0.806621	0.832
B cell	CD19 on IgD+ CD38dim	Inverse variance weighted	41	0.204653	0.009555	9.13E-102	0.185925	0.223382	1.227099	1.204332	1.250
B cell	CD19 on IgD- CD38dim	Inverse variance weighted	47	0.225632	0.01202	1.29E-78	0.202073	0.249192	1.253115	1.223938	1.282
B cell	CD19 on sw mem	Inverse variance weighted	43	0.215625	0.013206	6.28E-60	0.189741	0.241509	1.240637	1.208936	1.273
B cell	CD20 on IgD+ CD38dim	Inverse variance weighted	99	0.510932	0.022435	8.32E-115	0.466959	0.554904	1.666844	1.595137	1.741
B cell	CD20 on IgD- CD38dim	Inverse variance weighted	279	0.565087	0.0092	0	0.547054	0.58312	1.759601	1.728155	1.791
B cell	CD27 on CD24+ CD27+	Inverse variance weighted	101	0.350665	0.012727	3.97E-167	0.325721	0.375609	1.420012	1.38503	1.455
B cell	CD27 on unsw mem	Inverse variance weighted	66	0.312755	0.020784	3.57E-51	0.272019	0.353492	1.367187	1.312611	1.424

B cell	CD27 on sw mem	Inverse variance weighted	93	0.329536	0.012679	6.30E-149	0.304685	0.354387	1.390323	1.356198	1.425
B cell	CD38 on IgD+ CD24-	Inverse variance weighted	22	-0.21259	0.012697	6.32E-63	-0.23748	-0.1877	0.808488	0.788616	0.82
B cell	IgD on IgD+ CD38dim	Inverse variance weighted	35	0.45331	0.020052	3.73E-113	0.414008	0.492612	1.573512	1.512869	1.636
B cell	IgD on IgD+	Inverse variance weighted	35	0.457065	0.016799	5.23E-163	0.424139	0.489991	1.579432	1.528274	1.632
B cell	IgD on transitional	Inverse variance weighted	38	0.313065	0.014199	9.73E-108	0.285236	0.340894	1.367611	1.330076	1.406
TBNK	CD3 on NKT	Inverse variance weighted	22	0.374718	0.043733	1.05E-17	0.289	0.460435	1.454581	1.335092	1.584
Treg	CD3 on CD8br	Inverse variance weighted	41	-0.25115	0.015019	8.95E-63	-0.28059	-0.22172	0.777903	0.755337	0.801
Treg	CD127 on CD45RA- CD4 not Treg	Inverse variance weighted	18	0.278458	0.036081	1.19E-14	0.207739	0.349177	1.321091	1.230892	1.417
Myeloid cell	CD33 on CD66b++ myeloid cell	Inverse variance weighted	39	-0.16848	0.017522	6.90E-22	-0.20282	-0.13413	0.844951	0.816425	0.874
cDC	FSC-A on granulocyte	Inverse variance weighted	26	0.209962	0.022488	9.95E-21	0.165885	0.254038	1.233631	1.180438	1.289
Monocyte	CD40 on CD14+ CD16- monocyte	Inverse variance weighted	44	0.255912	0.016613	1.53E-53	0.22335	0.288473	1.291638	1.250258	1.334
Monocyte	CCR2 on CD14- CD16+ monocyte	Inverse variance weighted	29	0.23652	0.018531	2.63E-37	0.200199	0.272842	1.266833	1.221646	1.313
cDC	CCR2 on CD62L+ myeloid DC	Inverse variance weighted	34	-0.24464	0.018585	1.42E-39	-0.28106	-0.20821	0.782988	0.754981	0.812
cDC	CD80 on monocyte	Inverse variance weighted	69	-0.24786	0.016379	9.84E-52	-0.27996	-0.21576	0.780469	0.755812	0.805
cDC	SSC-A on monocyte	Inverse variance weighted	93	-0.20513	0.009427	5.51E-105	-0.22361	-0.18666	0.81454	0.799628	0.829
Treg	CD45RA on resting Treg	Inverse variance weighted	69	0.226021	0.02608	4.46E-18	0.174904	0.277139	1.253602	1.191132	1.31

Table S9. Full MR results for the associations between immune cell traits and neuroblastoma using different MR methods.

Panel	Trait	method	nsnp	b	se	pval
B cell	IgD- CD27- AC	MR Egger	20728	-0.00712	0.035355	0.840427
B cell	IgD- CD27- AC	Inverse variance weighted	20728	-0.00087	0.016194	0.956999
B cell	IgD- CD27- AC	Simple mode	20723	-2.21922	0.892499	0.012908
B cell	IgD- CD27- AC	Weighted mode	20723	-1.5654	0.941135	0.096266
B cell	IgD- CD38br %lymphocyte	MR Egger	22288	0.067733	0.035016	0.053085
B cell	IgD- CD38br	Inverse variance	2228	0.029026	0.01542	0.059784

	%lymphocyte	weighted	8			
B cell	IgD- CD38br %lymphocyte	Simple mode	22287	-2.37557	1.05355	0.024154
B cell	IgD- CD38br %lymphocyte	Weighted mode	22287	-1.49227	1.068064	0.162377
B cell	IgD- CD38dim %lymphocyte	MR Egger	21167	0.04126	0.035639	0.246989
B cell	IgD- CD38dim %lymphocyte	Inverse variance weighted	21167	0.009926	0.016074	0.536886
B cell	IgD- CD38dim %lymphocyte	Simple mode	21157	2.318581	0.762505	0.002363
B cell	IgD- CD38dim %lymphocyte	Weighted mode	21157	-1.37565	0.762119	0.071083
Treg	CD39+ resting Treg AC	MR Egger	20353	-0.05547	0.037197	0.13591
Treg	CD39+ resting Treg AC	Inverse variance weighted	20353	-0.02645	0.016763	0.114536
Treg	CD39+ resting Treg AC	Simple mode	20350	-2.36962	1.374233	0.084665
Treg	CD39+ resting Treg AC	Weighted mode	20350	1.586427	1.317109	0.22842
Treg	CD39+ resting Treg % CD4 Treg	MR Egger	21578	-0.09685	0.035953	0.007067
Treg	CD39+ resting Treg % CD4 Treg	Inverse variance weighted	21578	-0.02228	0.015922	0.161719
Treg	CD39+ resting Treg % CD4 Treg	Simple mode	21576	-2.39842	1.487308	0.106848
Treg	CD39+ resting Treg % CD4 Treg	Weighted mode	21576	1.69038	1.472207	0.250901
Treg	CD39+ activated Treg AC	MR Egger	20368	-0.00823	0.039309	0.834069
Treg	CD39+ activated Treg AC	Inverse variance weighted	20368	0.01399	0.017457	0.422902

Treg	CD39+ activated Treg AC	Simple mode	20362	2.505451	1.228769	0.041464
Treg	CD39+ activated Treg AC	Weighted mode	20362	-1.83143	1.161363	0.114818
Treg	CD25hi CD45RA+ CD4 not Treg AC	MR Egger	21308	-0.00147	0.037543	0.968813
Treg	CD25hi CD45RA+ CD4 not Treg AC	Inverse variance weighted	21308	0.007094	0.017061	0.677548
Treg	CD25hi CD45RA+ CD4 not Treg AC	Simple mode	21300	2.262136	1.245214	0.069283
Treg	CD25hi CD45RA+ CD4 not Treg AC	Weighted mode	21300	1.863823	1.151806	0.10564
Myeloid cell	CD33dim HLA DR- AC	MR Egger	22591	0.064392	0.046386	0.165097
Myeloid cell	CD33dim HLA DR- AC	Inverse variance weighted	22591	0.014694	0.021347	0.491226
Myeloid cell	CD33dim HLA DR- AC	Simple mode	22588	-3.19966	3.615898	0.376226
Myeloid cell	CD33dim HLA DR- AC	Weighted mode	22588	2.094837	3.843803	0.585765
B cell	Transitional AC	MR Egger	22580	-0.01714	0.034838	0.622655
B cell	Transitional AC	Inverse variance weighted	22580	-0.00269	0.015673	0.863864
B cell	Transitional AC	Simple mode	22576	2.467836	1.157573	0.033025
B cell	Transitional AC	Weighted mode	22576	1.470682	1.043631	0.158791
Monocyte	CD14- CD16+ monocyte AC	MR Egger	19748	-0.05347	0.036492	0.142882
Monocyte	CD14- CD16+ monocyte AC	Inverse variance weighted	19748	-0.01694	0.01648	0.304026
Monocyt	CD14- CD16+ monocyte	Simple mode	1974	2.264291	1.147837	0.048548

e	AC		4			
Monocyte	CD14- CD16+ monocyte AC	Weighted mode	19744	1.4593	1.154733	0.206333
Monocyte	CD14+ CD16- monocyte AC	MR Egger	19739	-0.04078	0.03601	0.257446
Monocyte	CD14+ CD16- monocyte AC	Inverse variance weighted	19739	0.006277	0.01644	0.70261
Monocyte	CD14+ CD16- monocyte AC	Simple mode	19735	-2.37538	1.320583	0.072076
Monocyte	CD14+ CD16- monocyte AC	Weighted mode	19735	-1.56302	1.275655	0.22049
TBNK	CD8dim AC	MR Egger	21156	0.021624	0.035941	0.547411
TBNK	CD8dim AC	Inverse variance weighted	21156	0.022787	0.016046	0.15559
TBNK	CD8dim AC	Simple mode	21154	-2.11212	1.553637	0.174013
TBNK	CD8dim AC	Weighted mode	21154	1.752362	1.596035	0.27224
TBNK	CD8dim %T cell	MR Egger	22619	-0.04445	0.035633	0.212295
TBNK	CD8dim %T cell	Inverse variance weighted	22619	0.02649	0.015951	0.096786
TBNK	CD8dim %T cell	Simple mode	22613	2.426394	1.395697	0.08214
TBNK	CD8dim %T cell	Weighted mode	22613	1.439092	1.318192	0.274969
TBNK	CD8dim %leukocyte	MR Egger	20161	-0.0279	0.036945	0.4501
TBNK	CD8dim %leukocyte	Inverse variance weighted	20161	0.010007	0.016899	0.553737
TBNK	CD8dim %leukocyte	Simple mode	20158	2.228797	1.246782	0.073849

TBNK	CD8dim %leukocyte	Weighted mode	20158	1.72978	1.212641	0.153752
TBNK	HLA DR+ CD4+ %lymphocyte	MR Egger	21282	-0.03794	0.031797	0.23276
TBNK	HLA DR+ CD4+ %lymphocyte	Inverse variance weighted	21282	-0.01034	0.01456	0.47778
TBNK	HLA DR+ CD4+ %lymphocyte	Simple mode	21273	-2.06907	1.032746	0.04514
TBNK	HLA DR+ CD4+ %lymphocyte	Weighted mode	21273	-1.1732	1.073892	0.274639
TBNK	CD8br NKT %T cell	MR Egger	21617	-0.10634	0.034764	0.002225
TBNK	CD8br NKT %T cell	Inverse variance weighted	21617	-0.0167	0.015855	0.292097
TBNK	CD8br NKT %T cell	Simple mode	21611	2.231443	1.066259	0.036381
TBNK	CD8br NKT %T cell	Weighted mode	21611	-1.4358	1.038038	0.166623
TBNK	CD8br NKT %lymphocyte	MR Egger	21332	-0.06475	0.034522	0.060718
TBNK	CD8br NKT %lymphocyte	Inverse variance weighted	21332	-0.00385	0.015906	0.808536
TBNK	CD8br NKT %lymphocyte	Simple mode	21326	2.24686	0.96958	0.020494
TBNK	CD8br NKT %lymphocyte	Weighted mode	21326	-1.38961	1.03299	0.178565
Treg	CD39+ CD4+ %CD4+	MR Egger	20203	0.032759	0.037321	0.380082
Treg	CD39+ CD4+ %CD4+	Inverse variance weighted	20203	0.01147	0.016932	0.498165
Treg	CD39+ CD4+ %CD4+	Simple mode	20201	-2.06556	1.956398	0.291074
Treg	CD39+ CD4+ %CD4+	Weighted mode	2020	-1.384	1.859419	0.456693

			1			
Treg	CD39+ CD8br AC	MR Egger	19027	0.017948	0.042152	0.670262
Treg	CD39+ CD8br AC	Inverse variance weighted	19027	-0.00703	0.019415	0.717453
Treg	CD39+ CD8br AC	Simple mode	19021	-2.3412	1.17723	0.046744
Treg	CD39+ CD8br AC	Weighted mode	19021	1.824331	1.24852	0.143978
Treg	CD28+ CD45RA+ CD8br AC	MR Egger	22288	-0.01952	0.025962	0.452237
Treg	CD28+ CD45RA+ CD8br AC	Inverse variance weighted	22288	0.001729	0.011603	0.881549
Treg	CD28+ CD45RA+ CD8br AC	Simple mode	22283	-1.63778	1.311478	0.211751
Treg	CD28+ CD45RA+ CD8br AC	Weighted mode	22283	-1.1515	1.225235	0.347321
B cell	BAFF-R on CD24+ CD27+	MR Egger	19521	0.034314	0.040513	0.397014
B cell	BAFF-R on CD24+ CD27+	Inverse variance weighted	19521	0.025746	0.01837	0.161043
B cell	BAFF-R on CD24+ CD27+	Simple mode	19519	2.328012	1.179595	0.048445
B cell	BAFF-R on CD24+ CD27+	Weighted mode	19519	1.601892	1.17521	0.172877
B cell	BAFF-R on IgD+ CD24+	MR Egger	19664	0.014072	0.040329	0.727145
B cell	BAFF-R on IgD+ CD24+	Inverse variance weighted	19664	0.022984	0.018362	0.210678
B cell	BAFF-R on IgD+ CD24+	Simple mode	19662	2.599827	1.275231	0.041492
B cell	BAFF-R on IgD+ CD24+	Weighted mode	1966	1.856067	1.30141	0.153827

			2			
B cell	BAFF-R on IgD+ CD24-	MR Egger	19980	0.016774	0.039737	0.672934
B cell	BAFF-R on IgD+ CD24-	Inverse variance weighted	19980	0.028711	0.018067	0.112032
B cell	BAFF-R on IgD+ CD24-	Simple mode	19975	2.437977	1.259776	0.052974
B cell	BAFF-R on IgD+ CD24-	Weighted mode	19975	1.652551	1.244317	0.184167
B cell	BAFF-R on IgD+ CD38- naive	MR Egger	22068	0.129438	0.048216	0.007268
B cell	BAFF-R on IgD+ CD38- naive	Inverse variance weighted	22068	0.008486	0.022138	0.701461
B cell	BAFF-R on IgD+ CD38- naive	Simple mode	22066	3.276196	1.419621	0.02102
B cell	BAFF-R on IgD+ CD38- naive	Weighted mode	22066	1.804342	1.40902	0.20036
B cell	BAFF-R on IgD+ CD38- unsw mem	MR Egger	22183	0.141797	0.049652	0.004297
B cell	BAFF-R on IgD+ CD38- unsw mem	Inverse variance weighted	22183	0.042391	0.022237	0.056605
B cell	BAFF-R on IgD+ CD38- unsw mem	Simple mode	22181	3.34912	2.152501	0.119741
B cell	BAFF-R on IgD+ CD38- unsw mem	Weighted mode	22181	1.661404	2.242307	0.458741
B cell	BAFF-R on IgD+ CD38dim	MR Egger	20231	-0.00262	0.039531	0.947173
B cell	BAFF-R on IgD+ CD38dim	Inverse variance weighted	20231	0.034046	0.018008	0.058683
B cell	BAFF-R on IgD+ CD38dim	Simple mode	20228	2.295112	1.281935	0.073413
B cell	BAFF-R on IgD+ CD38dim	Weighted mode	20228	1.507888	1.198789	0.208463

B cell	BAFF-R on IgD- CD27-	MR Egger	19204	0.028596	0.03975	0.471903
B cell	BAFF-R on IgD- CD27-	Inverse variance weighted	19204	0.014993	0.018287	0.412302
B cell	BAFF-R on IgD- CD27-	Simple mode	19200	-2.51654	1.315089	0.055687
B cell	BAFF-R on IgD- CD27-	Weighted mode	19200	1.342247	1.256055	0.285254
B cell	BAFF-R on IgD- CD38-	MR Egger	19384	0.02344	0.040715	0.564822
B cell	BAFF-R on IgD- CD38-	Inverse variance weighted	19384	0.036212	0.018555	0.050989
B cell	BAFF-R on IgD- CD38-	Simple mode	19379	2.241565	1.263404	0.076041
B cell	BAFF-R on IgD- CD38-	Weighted mode	19379	1.452048	1.326152	0.27356
B cell	BAFF-R on IgD- CD38br	MR Egger	21384	0.024873	0.036038	0.490082
B cell	BAFF-R on IgD- CD38br	Inverse variance weighted	21384	-0.00043	0.016076	0.978888
B cell	BAFF-R on IgD- CD38br	Simple mode	21381	-2.3504	1.371341	0.086554
B cell	BAFF-R on IgD- CD38br	Weighted mode	21381	-1.45076	1.287913	0.259989
B cell	BAFF-R on unsw mem	MR Egger	19704	0.022127	0.040025	0.580396
B cell	BAFF-R on unsw mem	Inverse variance weighted	19704	0.001409	0.01828	0.938557
B cell	BAFF-R on unsw mem	Simple mode	19701	-2.18839	1.232814	0.075894
B cell	BAFF-R on unsw mem	Weighted mode	19701	1.492093	1.167084	0.201095
B cell	BAFF-R on IgD+	MR Egger	2012	0.004201	0.039488	0.915278

			2			
B cell	BAFF-R on IgD+	Inverse variance weighted	20122	0.023032	0.018001	0.200736
B cell	BAFF-R on IgD+	Simple mode	20119	2.394887	1.219112	0.049492
B cell	BAFF-R on IgD+	Weighted mode	20119	1.610247	1.257702	0.200451
B cell	CD19 on IgD+ CD38dim	MR Egger	22991	-0.0225	0.035029	0.520741
B cell	CD19 on IgD+ CD38dim	Inverse variance weighted	22991	-0.00222	0.015714	0.887898
B cell	CD19 on IgD+ CD38dim	Simple mode	22982	-2.22278	0.837146	0.007932
B cell	CD19 on IgD+ CD38dim	Weighted mode	22982	-1.53323	0.848489	0.070773
B cell	CD19 on IgD- CD38dim	MR Egger	20972	-0.10786	0.037572	0.004101
B cell	CD19 on IgD- CD38dim	Inverse variance weighted	20972	0.002664	0.016268	0.869928
B cell	CD19 on IgD- CD38dim	Simple mode	20969	2.207347	0.916646	0.016046
B cell	CD19 on IgD- CD38dim	Weighted mode	20969	-1.36885	0.898859	0.127805
B cell	CD19 on sw mem	MR Egger	21411	-0.11219	0.037496	0.002775
B cell	CD19 on sw mem	Inverse variance weighted	21411	0.002974	0.016244	0.854711
B cell	CD19 on sw mem	Simple mode	21407	2.39595	0.798651	0.002703
B cell	CD19 on sw mem	Weighted mode	21407	-1.6221	0.836005	0.052356
B cell	CD20 on IgD+ CD38dim	MR Egger	22526	-0.03702	0.035735	0.30028

B cell	CD20 on IgD+ CD38dim	Inverse variance weighted	22526	0.010734	0.015904	0.499743
B cell	CD20 on IgD+ CD38dim	Simple mode	22523	-2.38817	1.354174	0.077819
B cell	CD20 on IgD+ CD38dim	Weighted mode	22523	1.374931	1.265758	0.277379
B cell	CD20 on IgD- CD38dim	MR Egger	21874	0.016992	0.033308	0.60996
B cell	CD20 on IgD- CD38dim	Inverse variance weighted	21874	0.016704	0.015773	0.289597
B cell	CD20 on IgD- CD38dim	Simple mode	21873	2.11578	2.070566	0.30687
B cell	CD20 on IgD- CD38dim	Weighted mode	21873	1.257746	1.994481	0.5283
B cell	CD27 on CD24+ CD27+	MR Egger	19495	0.099703	0.039618	0.011857
B cell	CD27 on CD24+ CD27+	Inverse variance weighted	19495	0.017037	0.017545	0.331544
B cell	CD27 on CD24+ CD27+	Simple mode	19492	-2.44732	1.089448	0.024691
B cell	CD27 on CD24+ CD27+	Weighted mode	19492	1.644949	1.035087	0.112034
B cell	CD27 on unsw mem	MR Egger	21854	-0.07568	0.039092	0.052902
B cell	CD27 on unsw mem	Inverse variance weighted	21854	-0.00121	0.016668	0.9419
B cell	CD27 on unsw mem	Simple mode	21849	-2.27721	1.091692	0.036995
B cell	CD27 on unsw mem	Weighted mode	21849	1.492233	1.131085	0.187085
B cell	CD27 on sw mem	MR Egger	20056	-0.00285	0.038751	0.941451

B cell	CD27 on sw mem	Inverse variance weighted	20056	0.01357	0.017164	0.429173
B cell	CD27 on sw mem	Simple mode	20051	2.177416	0.986682	0.027339
B cell	CD27 on sw mem	Weighted mode	20051	1.640031	0.94952	0.084143
B cell	CD38 on IgD+ CD24-	MR Egger	21653	-0.115	0.037122	0.001951
B cell	CD38 on IgD+ CD24-	Inverse variance weighted	21653	-0.02171	0.016422	0.186188
B cell	CD38 on IgD+ CD24-	Simple mode	21650	2.309019	1.877731	0.218828
B cell	CD38 on IgD+ CD24-	Weighted mode	21650	-1.42887	1.790137	0.42477
B cell	IgD on IgD+ CD38dim	MR Egger	21011	-0.05257	0.036691	0.151943
B cell	IgD on IgD+ CD38dim	Inverse variance weighted	21011	0.005206	0.01654	0.75295
B cell	IgD on IgD+ CD38dim	Simple mode	21005	2.337887	1.395931	0.093991
B cell	IgD on IgD+ CD38dim	Weighted mode	21005	1.405109	1.397073	0.314546
B cell	IgD on IgD+	MR Egger	20921	-0.04634	0.036833	0.208325
B cell	IgD on IgD+	Inverse variance weighted	20921	-0.00704	0.016667	0.672678
B cell	IgD on IgD+	Simple mode	20916	2.393327	2.043751	0.241594
B cell	IgD on IgD+	Weighted mode	20916	-1.82765	1.860791	0.326017
B cell	IgD on transitional	MR Egger	19859	-0.03295	0.03637	0.364933

B cell	IgD on transitional	Inverse variance weighted	19859	0.002933	0.017016	0.863149
B cell	IgD on transitional	Simple mode	19857	2.330406	1.322332	0.078026
B cell	IgD on transitional	Weighted mode	19857	1.365651	1.310751	0.297478
TBNK	CD3 on NKT	MR Egger	19772	0.005745	0.038916	0.882639
TBNK	CD3 on NKT	Inverse variance weighted	19772	-0.00559	0.017863	0.754217
TBNK	CD3 on NKT	Simple mode	19764	-2.43504	1.287447	0.058589
TBNK	CD3 on NKT	Weighted mode	19764	1.669304	1.280569	0.192397
Treg	CD3 on CD8br	MR Egger	20927	0.013957	0.039626	0.724676
Treg	CD3 on CD8br	Inverse variance weighted	20927	0.026362	0.017844	0.139585
Treg	CD3 on CD8br	Simple mode	20923	2.532399	1.575819	0.108061
Treg	CD3 on CD8br	Weighted mode	20923	-1.84522	1.564669	0.238291
Treg	CD127 on CD45RA- CD4 not Treg	MR Egger	21870	0.013735	0.039759	0.729764
Treg	CD127 on CD45RA- CD4 not Treg	Inverse variance weighted	21870	-0.00524	0.01768	0.767134
Treg	CD127 on CD45RA- CD4 not Treg	Simple mode	21867	-2.64551	1.090146	0.015243
Treg	CD127 on CD45RA- CD4 not Treg	Weighted mode	21867	-1.66935	1.001782	0.095652
Myeloid cell	CD33 on CD66b++ myeloid cell	MR Egger	21899	0.111107	0.055919	0.046943

Myeloid cell	CD33 on CD66b++ myeloid cell	Inverse variance weighted	21899	-0.01889	0.025309	0.455501
Myeloid cell	CD33 on CD66b++ myeloid cell	Simple mode	21896	-3.40597	1.331758	0.010549
Myeloid cell	CD33 on CD66b++ myeloid cell	Weighted mode	21896	2.230752	1.293987	0.084733
cDC	FSC-A on granulocyte	MR Egger	20335	0.090932	0.042164	0.031047
cDC	FSC-A on granulocyte	Inverse variance weighted	20335	0.008166	0.018932	0.666231
cDC	FSC-A on granulocyte	Simple mode	20333	-2.65216	1.474637	0.072109
cDC	FSC-A on granulocyte	Weighted mode	20333	1.780234	1.521606	0.242027
Monocyte	CD40 on CD14+ CD16- monocyte	MR Egger	20898	-0.04407	0.03868	0.254585
Monocyte	CD40 on CD14+ CD16- monocyte	Inverse variance weighted	20898	0.011894	0.016961	0.48313
Monocyte	CD40 on CD14+ CD16- monocyte	Simple mode	20896	2.105055	1.425451	0.139754
Monocyte	CD40 on CD14+ CD16- monocyte	Weighted mode	20896	1.624552	1.451359	0.263012
Monocyte	CCR2 on CD14- CD16+ monocyte	MR Egger	21175	0.150571	0.037316	5.48E-05
Monocyte	CCR2 on CD14- CD16+ monocyte	Inverse variance weighted	21175	-0.01422	0.016446	0.387189
Monocyte	CCR2 on CD14- CD16+ monocyte	Simple mode	21170	-2.06926	1.800273	0.250398
Monocyte	CCR2 on CD14- CD16+ monocyte	Weighted mode	21170	1.193643	1.733926	0.491206
cDC	CCR2 on CD62L+ myeloid DC	MR Egger	20834	0.016738	0.041894	0.689504

cDC	CCR2 on CD62L+ myeloid DC	Inverse variance weighted	20834	-0.02286	0.018719	0.221922
cDC	CCR2 on CD62L+ myeloid DC	Simple mode	20830	-2.47515	1.126208	0.027976
cDC	CCR2 on CD62L+ myeloid DC	Weighted mode	20830	-1.84484	1.107569	0.095794
cDC	CD80 on monocyte	MR Egger	20915	0.029586	0.042425	0.485576
cDC	CD80 on monocyte	Inverse variance weighted	20915	0.014705	0.018823	0.434659
cDC	CD80 on monocyte	Simple mode	20912	2.312799	2.607057	0.375019
cDC	CD80 on monocyte	Weighted mode	20912	2.312799	2.551906	0.364786
cDC	SSC-A on monocyte	MR Egger	20599	-0.01543	0.041094	0.707215
cDC	SSC-A on monocyte	Inverse variance weighted	20599	0.023445	0.018595	0.207379
cDC	SSC-A on monocyte	Simple mode	20594	2.506646	1.06046	0.018101
cDC	SSC-A on monocyte	Weighted mode	20594	1.53501	0.985053	0.119177
Treg	CD45RA on resting Treg	MR Egger	22973	-0.08012	0.03753	0.032798
Treg	CD45RA on resting Treg	Inverse variance weighted	22973	-0.03081	0.016313	0.058965
Treg	CD45RA on resting Treg	Simple mode	22968	-2.18926	2.010751	0.276264
Treg	CD45RA on resting Treg	Weighted mode	22968	-1.73684	2.071754	0.401847

Table S10. Two-sample MR estimates for the associations between the 20 prioritized genes and neuroblastoma risk using different MR

methods.

gene	method	nsnp	b	se	pval	lo_ci	up_ci	or	or_lci95	or_uci95
LINC00339	MR Egger	76	0.442461	0.043668	1.25E-15	0.356871	0.52805	1.556532	1.428851	1.695623
LINC00339	Weighted median	76	0.125101	0.044609	0.005042	0.037666	0.212535	1.133263	1.038385	1.23681
LINC00339	Inverse variance weighted	76	0.023661	0.029502	0.422539	-0.03416	0.081485	1.023943	0.966414	1.084897
LINC00339	Simple mode	76	-0.33222	0.017339	1.29E-30	-0.36621	-0.29824	0.717327	0.693358	0.742124
LINC00339	Weighted mode	76	0.173243	0.013378	8.22E-21	0.147023	0.199464	1.189156	1.158381	1.220748
KIAA1522	MR Egger	11	0.626506	0.14448	0.001888	0.343324	0.909687	1.871061	1.409626	2.483546
KIAA1522	Weighted median	11	0.280536	0.024487	2.18E-30	0.232541	0.328531	1.323839	1.261802	1.388926
KIAA1522	Inverse variance weighted	11	0.154939	0.090219	0.085913	-0.02189	0.331769	1.167587	0.978347	1.393431
KIAA1522	Simple mode	11	0.278565	0.03321	7.76E-06	0.213473	0.343657	1.321232	1.23797	1.410095
KIAA1522	Weighted mode	11	0.252685	0.022278	4.95E-07	0.20902	0.296349	1.287477	1.23247	1.34494
FCGR2A	MR Egger	3	1.20136	12.17154	0.937367	-22.6549	25.05759	3.324636	1.45E-10	7.63E+10
FCGR2A	Weighted median	3	0.400171	1.000797	0.689265	-1.56139	2.361733	1.49208	0.209844	10.60932
FCGR2A	Inverse variance weighted	3	0.393937	1.006645	0.695548	-1.57909	2.366961	1.482808	0.206163	10.66493
FCGR2A	Simple mode	3	0.409169	2.382902	0.879468	-4.26132	5.079656	1.505566	0.014104	160.7188
FCGR2A	Weighted mode	3	0.399131	1.02233	0.73389	-1.60464	2.402899	1.490529	0.200963	11.05518
MTA3	MR Egger	8	0.384353	6.101868	0.951821	-11.5753	12.34401	1.468664	9.40E-06	229581.7
MTA3	Weighted median	8	0.252819	1.870951	0.89251	-3.41425	3.919883	1.28765	0.032901	50.39454
MTA3	Inverse variance weighted	8	0.156381	1.563388	0.920323	-2.90786	3.220622	1.169272	0.054592	25.0437
MTA3	Simple mode	8	0.407376	2.649035	0.882121	-4.78473	5.599483	1.502869	0.008356	270.2867
MTA3	Weighted mode	8	0.302075	1.823239	0.873092	-3.27147	3.875624	1.352663	0.03795	48.21279
ATRIP	MR Egger	4	0.529772	4.947911	0.924506	-9.16813	10.22768	1.698545	0.000104	27658.17
ATRIP	Weighted median	4	0.311842	0.913002	0.732684	-1.47764	2.101326	1.365939	0.228175	8.177007
ATRIP	Inverse variance weighted	4	0.289799	0.90071	0.747646	-1.47559	2.05519	1.336159	0.228643	7.808318
ATRIP	Simple mode	4	0.234255	1.759302	0.902503	-3.21398	3.682487	1.263967	0.040196	39.74513
ATRIP	Weighted mode	4	0.317363	0.989544	0.769467	-1.62214	2.256869	1.373502	0.197475	9.553132

PPP2R5CP	Inverse variance weighted	2	0.204466	2.397699	0.932042	-4.49502	4.903955	1.22687	0.011164	134.822
IMPG2	MR Egger	7	-0.82396	12.04516	0.948114	-24.4325	22.78456	0.438689	2.45E-11	7.86E+09
IMPG2	Weighted median	7	-0.20123	1.842061	0.913012	-3.81167	3.409214	0.817727	0.022111	30.24145
IMPG2	Inverse variance weighted	7	-0.13336	1.614298	0.93416	-3.29738	3.030663	0.87515	0.03698	20.71096
IMPG2	Simple mode	7	-0.20945	2.333022	0.931387	-4.78217	4.363273	0.81103	0.008378	78.51366
IMPG2	Weighted mode	7	-0.20477	2.09642	0.925371	-4.31375	3.904213	0.814835	0.013383	49.61104
PLSCR1	MR Egger	4	-0.40896	1.520988	0.813222	-3.39009	2.572181	0.664344	0.033706	13.09435
PLSCR1	Weighted median	4	-0.19255	0.160948	0.231556	-0.50801	0.122906	0.824852	0.601692	1.130778
PLSCR1	Inverse variance weighted	4	-0.19235	0.154381	0.212781	-0.49494	0.110235	0.825016	0.609608	1.11654
PLSCR1	Simple mode	4	-0.20489	0.477765	0.696964	-1.14131	0.73153	0.814738	0.319401	2.078258
PLSCR1	Weighted mode	4	-0.19481	0.157626	0.304442	-0.50376	0.114135	0.82299	0.604256	1.120904
P3H2	MR Egger	19	-0.94605	0.068083	1.04E-10	-1.07949	-0.81261	0.388273	0.339769	0.4437
P3H2	Weighted median	19	-0.20868	0.020795	1.07E-23	-0.24944	-0.16792	0.811655	0.779239	0.84542
P3H2	Inverse variance weighted	19	0.051783	0.08637	0.548804	-0.1175	0.221068	1.053148	0.889139	1.247409
P3H2	Simple mode	19	-0.25902	0.104416	0.023221	-0.46368	-0.05437	0.771805	0.628965	0.947084
P3H2	Weighted mode	19	-0.21602	0.025206	9.07E-08	-0.26542	-0.16662	0.805719	0.766881	0.846525
MB21D2	MR Egger	3	0.56528	6.885172	0.94785	-12.9297	14.06022	1.75994	2.43E-06	1277246
MB21D2	Weighted median	3	-0.03811	2.834672	0.989274	-5.59406	5.51785	0.962611	0.00372	249.099
MB21D2	Inverse variance weighted	3	-0.01589	2.688412	0.995284	-5.28518	5.253396	0.984234	0.005066	191.2145
MB21D2	Simple mode	3	-0.36281	3.770938	0.932125	-7.75385	7.028232	0.695721	0.000429	1128.035
MB21D2	Weighted mode	3	0.158595	3.256304	0.965581	-6.22376	6.540952	1.171864	0.001982	692.946
DCAF16	MR Egger	4	0.001463	2.985141	0.999653	-5.84941	5.852341	1.001464	0.002882	348.0481
DCAF16	Weighted median	4	-0.18802	0.980658	0.847955	-2.11011	1.734069	0.828598	0.121225	5.663653
DCAF16	Inverse variance weighted	4	-0.18701	0.976775	0.848164	-2.10149	1.727464	0.829432	0.122274	5.626368
DCAF16	Simple mode	4	-0.47425	2.050072	0.831935	-4.4924	3.543888	0.622349	0.011194	34.60119
DCAF16	Weighted mode	4	-0.19978	1.031026	0.858735	-2.22059	1.82103	0.81891	0.108545	6.178219
FAM114A2	MR Egger	4	-0.14644	5.161002	0.97994	-10.262	9.969126	0.863778	3.49E-05	21356.8

FAM114A2	Weighted median	4	-0.34043	0.834611	0.683352	-1.97627	1.295405	0.711463	0.138585	3.652475
FAM114A2	Inverse variance weighted	4	-0.35284	0.733297	0.630398	-1.7901	1.084425	0.702691	0.166943	2.957738
FAM114A2	Simple mode	4	-0.40429	0.912881	0.687847	-2.19354	1.384959	0.667452	0.111522	3.99466
FAM114A2	Weighted mode	4	-0.33172	0.886895	0.733256	-2.07004	1.406593	0.717687	0.126181	4.082024
RANBP17	MR Egger	12	-0.22038	7.75134	0.977877	-15.413	14.97224	0.802211	2.02E-07	3179527
RANBP17	Weighted median	12	-0.39575	2.454091	0.871887	-5.20577	4.414267	0.673174	0.005485	82.62129
RANBP17	Inverse variance weighted	12	-0.29141	1.944739	0.880888	-4.10309	3.520282	0.747212	0.016521	33.79395
RANBP17	Simple mode	12	-0.4671	3.395184	0.893061	-7.12166	6.187462	0.626818	0.000807	486.6095
RANBP17	Weighted mode	12	-0.46969	2.671324	0.863626	-5.70548	4.766108	0.625198	0.003328	117.4612
USP45	MR Egger	5	0.592804	15.90328	0.972607	-30.5776	31.76322	1.809054	5.25E-14	6.23E+13
USP45	Weighted median	5	0.369441	1.295739	0.775552	-2.17021	2.909089	1.446926	0.114154	18.34008
USP45	Inverse variance weighted	5	0.345805	1.090175	0.751091	-1.79094	2.482548	1.413126	0.166803	11.97173
USP45	Simple mode	5	0.371153	1.521966	0.819333	-2.6119	3.354207	1.449405	0.073395	28.62289
USP45	Weighted mode	5	0.36798	1.488381	0.816898	-2.54925	3.285208	1.444813	0.07814	26.71453
RPS3P5	MR Egger	5	-0.46501	2.085511	0.837876	-4.55261	3.622589	0.628128	0.01054	37.43436
RPS3P5	Weighted median	5	-0.11442	0.428371	0.789394	-0.95402	0.725191	0.891886	0.385188	2.065125
RPS3P5	Inverse variance weighted	5	-0.09648	0.357947	0.787509	-0.79806	0.605093	0.908025	0.450202	1.831422
RPS3P5	Simple mode	5	-0.12282	0.491031	0.814807	-1.08524	0.839597	0.884421	0.33782	2.315435
RPS3P5	Weighted mode	5	-0.12184	0.538234	0.832008	-1.17678	0.933096	0.885288	0.30827	2.542368
SEMA3C	MR Egger	3	1.425422	12.74359	0.929086	-23.552	26.40286	4.159612	5.91E-11	2.93E+11
SEMA3C	Weighted median	3	-0.10335	2.253796	0.963425	-4.52079	4.314091	0.901811	0.01088	74.74562
SEMA3C	Inverse variance weighted	3	-0.02729	1.99134	0.989064	-3.93032	3.875732	0.973076	0.019637	48.21798
SEMA3C	Simple mode	3	-0.28835	2.737547	0.925725	-5.65394	5.077241	0.749498	0.003504	160.3311
SEMA3C	Weighted mode	3	-0.28354	2.636237	0.924166	-5.45057	4.883485	0.753113	0.004294	132.0902
GTPBP10	MR Egger	28	0.937309	0.597979	0.129098	-0.23473	2.109347	2.553103	0.790785	8.242861
GTPBP10	Weighted median	28	0.27413	0.060747	6.40E-06	0.155067	0.393194	1.315386	1.167736	1.481705
GTPBP10	Inverse variance	28	0.035548	0.061052	0.560398	-0.08411	0.15521	1.036187	0.919326	1.167903

	weighted									
GTPBP10	Simple mode	28	-0.27915	0.10934	0.016645	-0.49346	-0.06484	0.756427	0.610513	0.937216
GTPBP10	Weighted mode	28	0.307004	0.031072	1.84E-10	0.246102	0.367906	1.359346	1.27903	1.444706
ZNF696	MR Egger	14	0.190578	0.575982	0.746444	-0.93835	1.319502	1.209949	0.391275	3.741558
ZNF696	Weighted median	14	0.203685	0.165279	0.217812	-0.12026	0.527633	1.225912	0.886687	1.694915
ZNF696	Inverse variance weighted	14	0.191064	0.15854	0.228146	-0.11967	0.501802	1.210537	0.88721	1.651695
ZNF696	Simple mode	14	-0.11822	0.554177	0.834384	-1.20441	0.967969	0.888502	0.29987	2.632592
ZNF696	Weighted mode	14	0.203523	0.168891	0.249661	-0.1275	0.534548	1.225713	0.880291	1.706677
HGH1	Wald ratio	1	-0.21236	3.400303	0.950201	-6.87696	6.452231	0.808672	0.001031	634.1155
SMC2	MR Egger	10	0.263986	4.609373	0.955733	-8.77038	9.298357	1.30211	0.000155	10920.06
SMC2	Weighted median	10	-0.34893	0.743278	0.638747	-1.80576	1.107891	0.70544	0.16435	3.027966
SMC2	Inverse variance weighted	10	-0.30044	0.620981	0.628523	-1.51756	0.916687	0.740496	0.219247	2.500992
SMC2	Simple mode	10	-0.28424	0.926473	0.765979	-2.10012	1.53165	0.752589	0.122441	4.625801
SMC2	Weighted mode	10	-0.33843	0.825171	0.691295	-1.95577	1.278901	0.712885	0.141455	3.592687
RNLS	MR Egger	3	-1.0922	8.827591	0.921632	-18.3943	16.20987	0.335477	1.03E-08	10961227
RNLS	Weighted median	3	-0.17326	3.735052	0.963001	-7.49396	7.147441	0.840919	0.000556	1270.85
RNLS	Inverse variance weighted	3	0.026541	3.466413	0.993891	-6.76763	6.82071	1.026897	0.00115	916.6359
RNLS	Simple mode	3	-0.57127	4.263381	0.905673	-8.9275	7.784953	0.564805	0.000133	2404.152
RNLS	Weighted mode	3	0.508503	4.000731	0.910486	-7.33293	8.349937	1.662801	0.000654	4229.914
RPP30	MR Egger	3	0.212663	5.117147	0.973558	-9.81694	10.24227	1.236968	5.45E-05	28064.78
RPP30	Weighted median	3	0.416163	0.936182	0.656657	-1.41875	2.251079	1.516134	0.242016	9.497982
RPP30	Inverse variance weighted	3	0.429952	0.92171	0.640878	-1.3766	2.236504	1.537184	0.252435	9.360553
RPP30	Simple mode	3	0.418959	1.345075	0.784908	-2.21739	3.055305	1.520378	0.108893	21.22766
RPP30	Weighted mode	3	0.414908	0.928313	0.698652	-1.40459	2.234401	1.514231	0.245469	9.340884
GAPDH	MR Egger	4	0.190008	3.56577	0.962347	-6.7989	7.178917	1.209259	0.001115	1311.487
GAPDH	Weighted median	4	-0.23125	0.326768	0.479135	-0.87172	0.409214	0.79354	0.418233	1.505633
GAPDH	Inverse variance weighted	4	-0.22933	0.321554	0.475723	-0.85958	0.400914	0.795065	0.423341	1.493189
GAPDH	Simple mode	4	0.35298	0.98892	0.744771	-1.5853	2.291263	1.423303	0.204886	9.88742

GAPDH	Weighted mode	4	-0.24296	0.346958	0.534185	-0.923	0.437076	0.784302	0.397326	1.548174
CLEC4D	MR Egger	531	0.317443	0.023976	9.15E-35	0.270451	0.364436	1.373612	1.310555	1.439702
CLEC4D	Weighted median	531	-0.08315	0.037345	0.025975	-0.15635	-0.00996	0.920211	0.855262	0.990094
CLEC4D	Inverse variance weighted	531	0.02048	0.010713	0.055903	-0.00052	0.041477	1.020691	0.999484	1.042349
CLEC4D	Simple mode	531	0.109956	0.067458	0.103697	-0.02226	0.242173	1.116229	0.977985	1.274015
CLEC4D	Weighted mode	531	0.19728	0.034941	2.68E-08	0.128797	0.265764	1.218086	1.137459	1.304427
KLRB1	MR Egger	811	0.026821	0.115435	0.816327	-0.19943	0.253074	1.027184	0.819196	1.287978
KLRB1	Weighted median	811	0.225041	0.060228	0.000187	0.106994	0.343089	1.252375	1.112928	1.409294
KLRB1	Inverse variance weighted	811	0.05426	0.032197	0.091946	-0.00885	0.117366	1.055759	0.991192	1.124531
KLRB1	Simple mode	811	0.250664	0.095041	0.008514	0.064384	0.436945	1.284879	1.066501	1.547971
KLRB1	Weighted mode	811	0.269992	0.050677	1.29E-07	0.170665	0.369319	1.309954	1.186094	1.446749
IGHV6-1	MR Egger	3	-10.0928	238.1414	0.973035	-476.85	456.6644	4.14E-05	8.066E-208	2.122E198
IGHV6-1	Weighted median	3	-0.22532	1.689069	0.89388	-3.53589	3.08526	0.798264	0.029133	21.87315
IGHV6-1	Inverse variance weighted	3	-0.19615	1.516127	0.89706	-3.16776	2.775459	0.821889	0.042098	16.04599
IGHV6-1	Simple mode	3	-0.22541	2.052396	0.922572	-4.24811	3.797284	0.798187	0.014291	44.57993
IGHV6-1	Weighted mode	3	-0.22541	1.736156	0.908578	-3.62828	3.177454	0.798187	0.026562	23.98562
IGHV3-43	MR Egger	18	-0.09142	0.71545	0.899913	-1.49371	1.31086	0.912632	0.224539	3.709362
IGHV3-43	Weighted median	18	-0.09094	0.299844	0.76166	-0.67864	0.49675	0.91307	0.507308	1.643372
IGHV3-43	Inverse variance weighted	18	-0.02016	0.235473	0.931774	-0.48169	0.441368	0.980042	0.61774	1.554833
IGHV3-43	Simple mode	18	-0.11451	0.378513	0.765912	-0.8564	0.627371	0.891799	0.424688	1.872681
IGHV3-43	Weighted mode	18	-0.09949	0.339399	0.772964	-0.76471	0.565731	0.905298	0.465467	1.760735
IGHV3-49	MR Egger	6	-0.02808	17.22567	0.998777	-33.7904	33.73423	0.97231	2.11E-15	4.47E+14
IGHV3-49	Weighted median	6	0.391757	1.463721	0.788973	-2.47714	3.260651	1.479578	0.083983	26.06649
IGHV3-49	Inverse variance weighted	6	0.368423	1.279919	0.773462	-2.14022	2.877065	1.445454	0.117629	17.76206
IGHV3-49	Simple mode	6	0.382895	1.830827	0.842594	-3.20553	3.971316	1.466524	0.040538	53.05431
IGHV3-49	Weighted mode	6	0.392294	1.704411	0.827084	-2.94835	3.73294	1.480373	0.052426	41.80183

IGHV1-58	MR Egger	13	-0.28606	5.110402	0.956364	-10.3025	9.730326	0.751216	3.36E-05	16820.03
IGHV1-58	Weighted median	13	-0.35884	0.80578	0.656081	-1.93817	1.220491	0.698487	0.143968	3.388851
IGHV1-58	Inverse variance weighted	13	-0.32286	0.678909	0.634393	-1.65352	1.007804	0.724077	0.191376	2.739578
IGHV1-58	Simple mode	13	-0.36097	1.093078	0.746918	-2.5034	1.781464	0.697	0.081806	5.938544
IGHV1-58	Weighted mode	13	-0.35627	0.731505	0.635012	-1.79001	1.077484	0.700287	0.166958	2.937281
IGHVIII-67-4	MR Egger	18	-0.06726	0.732249	0.92795	-1.50247	1.367944	0.934948	0.222579	3.927268
IGHVIII-67-4	Weighted median	18	-0.07487	0.249879	0.764467	-0.56463	0.414894	0.927865	0.56857	1.51421
IGHVIII-67-4	Inverse variance weighted	18	-0.05292	0.207587	0.798767	-0.45979	0.353947	0.948453	0.631414	1.42468
IGHVIII-67-4	Simple mode	18	-0.08231	0.343992	0.813744	-0.75654	0.591912	0.920984	0.469288	1.807441
IGHVIII-67-4	Weighted mode	18	-0.08378	0.311899	0.791464	-0.6951	0.527544	0.919635	0.499024	1.694765
MTFMT	MR Egger	5	2.039066	0.276252	0.005142	1.497612	2.58052	7.683429	4.471	13.204
MTFMT	Weighted median	5	0.295483	0.090628	0.001113	0.117853	0.473114	1.343776	1.125078	1.604985
MTFMT	Inverse variance weighted	5	0.154249	0.159688	0.334075	-0.15874	0.467238	1.166781	0.853218	1.595581
MTFMT	Simple mode	5	0.344445	0.621388	0.608893	-0.87348	1.562366	1.411206	0.417498	4.770092
MTFMT	Weighted mode	5	0.338007	0.079751	0.013282	0.181695	0.49432	1.402151	1.199248	1.639383
CFAP161	MR Egger	6	0.70619	3.039726	0.827692	-5.25167	6.664054	2.026257	0.005239	783.7214
CFAP161	Weighted median	6	0.388714	1.079424	0.718763	-1.72696	2.504385	1.475083	0.177825	12.23603
CFAP161	Inverse variance weighted	6	0.29641	0.920087	0.747336	-1.50696	2.09978	1.345021	0.221582	8.164373
CFAP161	Simple mode	6	0.404061	1.489673	0.797048	-2.5157	3.32382	1.497895	0.080806	27.76621
CFAP161	Weighted mode	6	0.154679	1.220755	0.90411	-2.238	2.547358	1.167283	0.106672	12.77331
XPO6	MR Egger	4	-0.01669	3.772959	0.996872	-7.41169	7.378313	0.983451	0.000604	1600.886
XPO6	Weighted median	4	0.057245	1.063591	0.957077	-2.02739	2.141884	1.058915	0.131678	8.515463
XPO6	Inverse variance weighted	4	0.061591	1.007975	0.951277	-1.91404	2.037221	1.063527	0.147483	7.66927
XPO6	Simple mode	4	-0.09405	1.518967	0.954525	-3.07122	2.883128	0.91024	0.046364	17.87009
XPO6	Weighted mode	4	0.095446	1.168458	0.940041	-2.19473	2.385623	1.100149	0.111389	10.86583
LPCAT2	MR Egger	37	-0.0697	0.255744	0.78682	-0.57095	0.431561	0.932677	0.564986	1.53966
LPCAT2	Weighted median	37	-0.2004	0.017305	5.16E-31	-0.23432	-0.16648	0.818403	0.79111	0.846637

LPCAT2	Inverse variance weighted	37	-0.07334	0.051554	0.154866	-0.17439	0.027708	0.929286	0.839973	1.028095
LPCAT2	Simple mode	37	-0.32108	0.083326	0.000462	-0.4844	-0.15776	0.725366	0.616068	0.854054
LPCAT2	Weighted mode	37	-0.24264	0.026691	7.44E-11	-0.29496	-0.19033	0.78455	0.744563	0.826686
ARL17A	MR Egger	10	-0.30928	0.420272	0.482808	-1.13301	0.514455	0.733977	0.322062	1.672726
ARL17A	Weighted median	10	-0.15854	0.014584	1.59E-27	-0.18712	-0.12995	0.85339	0.829341	0.878136
ARL17A	Inverse variance weighted	10	-0.07164	0.047511	0.131589	-0.16476	0.021482	0.930865	0.848095	1.021714
ARL17A	Simple mode	10	0.155417	0.080416	0.085308	-0.0022	0.313033	1.168145	0.997804	1.367567
ARL17A	Weighted mode	10	-0.15836	0.026464	0.000207	-0.21023	-0.10649	0.853545	0.810401	0.898985
EEF1DP7	Wald ratio	1	-0.3715	5.994741	0.950586	-12.1212	11.3782	0.689701	5.44E-06	87395.18
DDX42	Wald ratio	1	0.469352	7.573799	0.950586	-14.3753	15.314	1.598958	5.71E-07	4474916
LOC101928517	Wald ratio	1	-0.12237	1.971168	0.950499	-3.98586	3.741119	0.88482	0.018576	42.14512
PPP1R3D	Wald ratio	1	0.488918	12.82994	0.969602	-24.6578	25.63559	1.630551	1.96E-11	1.36E+11
BAIAP2L2	MR Egger	8	0.705176	0.025814	1.59E-07	0.65458	0.755772	2.024203	1.924335	2.129255
BAIAP2L2	Weighted median	8	-0.22391	0.128996	0.082596	-0.47675	0.028919	0.799384	0.6208	1.029341
BAIAP2L2	Inverse variance weighted	8	0.140697	0.120896	0.244512	-0.09626	0.377654	1.151076	0.908228	1.458858
BAIAP2L2	Simple mode	8	-0.44706	0.07233	0.000454	-0.58882	-0.30529	0.639507	0.554979	0.736909
BAIAP2L2	Weighted mode	8	0.246423	0.247985	0.353469	-0.23963	0.732475	1.279441	0.78692	2.080222
PHF5A	MR Egger	5	-0.08691	5.043897	0.987335	-9.97295	9.79913	0.916761	4.66E-05	18018.06
PHF5A	Weighted median	5	-0.2191	1.034374	0.832251	-2.24647	1.808276	0.803244	0.105772	6.099924
PHF5A	Inverse variance weighted	5	-0.20393	0.887331	0.818229	-1.9431	1.53524	0.81552	0.143259	4.642438
PHF5A	Simple mode	5	-0.22968	1.186629	0.855957	-2.55547	2.096116	0.794791	0.077656	8.134517
PHF5A	Weighted mode	5	-0.22402	1.068367	0.844163	-2.31802	1.869983	0.799301	0.098469	6.488187
CSDC2	MR Egger	5	-0.16309	5.856034	0.979531	-11.6409	11.31474	0.849516	8.80E-06	82021.62
CSDC2	Weighted median	5	0.307672	1.592555	0.846807	-2.81374	3.429079	1.360254	0.05998	30.84822
CSDC2	Inverse variance weighted	5	0.261097	1.498865	0.861711	-2.67668	3.198873	1.298354	0.068791	24.5049
CSDC2	Simple mode	5	-0.30365	2.435131	0.906779	-5.07651	4.469203	0.738116	0.006242	87.28711
CSDC2	Weighted mode	5	0.288919	1.703185	0.873531	-3.04932	3.62716	1.334983	0.047391	37.60588
PACSIN2	MR Egger	6	1.516281	0.122374	0.000244	1.276429	1.756134	4.555255	3.583818	5.790011

PACSIN2	Weighted median	6	0.21071	0.076829	0.006095	0.060126	0.361295	1.234555	1.061971	1.435186
PACSIN2	Inverse variance weighted	6	0.143206	0.11163	0.199541	-0.07559	0.362	1.153967	0.927197	1.436199
PACSIN2	Simple mode	6	-0.2625	0.565795	0.66218	-1.37146	0.846456	0.769125	0.253736	2.33137
PACSIN2	Weighted mode	6	0.271812	0.049084	0.002635	0.175607	0.368017	1.31234	1.19197	1.444866

Table S11. MR estimates for the associations between the 20 prioritized genes and immune cell traits in the mediation analyses.

Gene	Panel	Trait	method	nsnp	b	se	pval	lo_ci	up_ci	or	or_l
GAPDH	B cell	IgD- CD27- AC	Inverse variance weighted	3	1.59983	0.217253	1.79E-13	1.174013	2.025646	4.952188	3
KLRB1	B cell	IgD- CD38br %lymphocyte	Inverse variance weighted	218	0.506379	0.016546	1.072E-205	0.473949	0.538809	1.659272	1
HGH1	B cell	IgD- CD38dim %lymphocyte	Inverse variance weighted	2	-0.94565	0.146206	9.94E-11	-1.23221	-0.65908	0.388429	0
RNLS	Treg	CD39+ resting Treg AC	Inverse variance weighted	25	-0.7511	0.073704	2.18E-24	-0.89556	-0.60664	0.471849	
RANBP17	Treg	CD39+ resting Treg % CD4 Treg	Inverse variance weighted	10	0.732066	0.115478	2.31E-10	0.50573	0.958402	2.079373	1
RNLS	Treg	CD39+ resting Treg % CD4 Treg	Inverse variance weighted	94	-0.83856	0.038922	5.946E-103	-0.91485	-0.76227	0.432333	0
SEMA3C	Treg	CD39+ activated Treg AC	Inverse variance weighted	3	-0.87872	0.122083	6.12E-13	-1.11801	-0.63944	0.415313	0
RPP30	Treg	CD39+ activated Treg AC	Inverse variance weighted	3	-1.74393	0.254413	7.14E-12	-2.24258	-1.24528	0.174831	0
PLSCR1	Treg	CD25hi CD45RA+ CD4 not Treg AC	Inverse variance weighted	8	0.455676	0.052214	2.61E-18	0.353337	0.558014	1.577239	1
LOC101928517	Myeloid cell	CD33dim HLA DR- AC	Wald ratio	1	0.497753	0.083923	3.01E-09	0.333263	0.662243	1.645021	1
FAM114A2	B cell	Transitional AC	Inverse variance weighted	4	0.866628	0.135887	1.80E-10	0.60029	1.132966	2.378877	1
FCGR2A	Monocyte	CD14- CD16+ monocyte AC	Wald ratio	1	2.75471	0.411838	2.25E-11	1.947508	3.561911	15.71648	7
P3H2	Monocyte	CD14+ CD16- monocyte AC	Inverse variance weighted	6	-1.21926	0.175653	3.89E-12	-1.56354	-0.87498	0.29545	0
USP45	TBNK	CD8dim AC	Inverse variance weighted	3	1.585853	0.234802	1.44E-11	1.125641	2.046064	4.883453	3
RPS3P5	TBNK	CD8dim AC	Inverse variance weighted	3	-0.44634	0.066112	1.47E-11	-0.57592	-0.31676	0.639964	0
RPS3P5	TBNK	CD8dim %T cell	Inverse variance weighted	4	-0.49243	0.063891	1.28E-14	-0.61766	-0.36721	0.611138	0
RPS3P5	TBNK	CD8dim %leukocyte	Inverse variance weighted	4	-0.47847	0.06475	1.47E-13	-0.60538	-0.35156	0.619734	0
PPP1R3D	TBNK	HLA DR+ CD4+ %lymphocyte	Inverse variance weighted	9	0.732857	0.117119	3.92E-10	0.503303	0.962411	2.081019	1

HGH1	TBNK	CD8br NKT %T cell	Inverse variance weighted	2	0.965194	0.147191	5.48E-11	0.676699	1.253688	2.625296	1
HGH1	TBNK	CD8br NKT %lymphocyte	Inverse variance weighted	2	0.977901	0.148089	4.02E-11	0.687646	1.268156	2.65887	1
CFAP161	Treg	CD39+ CD4+ %CD4+	Inverse variance weighted	9	0.981539	0.14098	3.35E-12	0.705219	1.257859	2.668561	2
RPP30	Treg	CD39+ CD8br AC	Inverse variance weighted	3	-1.83893	0.256294	7.23E-13	-2.34126	-1.33659	0.158988	0
LPCAT2	Treg	CD28+ CD45RA+ CD8br AC	Inverse variance weighted	15	0.403105	0.063388	2.03E-10	0.278863	0.527346	1.496463	1
PHF5A	B cell	BAFF-R on CD24+ CD27+	Inverse variance weighted	5	-1.30988	0.193923	1.43E-11	-1.68997	-0.92979	0.269853	0
PHF5A	B cell	BAFF-R on IgD+ CD24+	Inverse variance weighted	5	-1.32282	0.194764	1.11E-11	-1.70456	-0.94108	0.266383	0
BAIAP2L2	B cell	BAFF-R on IgD+ CD24-	Inverse variance weighted	3	0.841725	0.107709	5.51E-15	0.630614	1.052835	2.320365	1
BAIAP2L2	B cell	BAFF-R on IgD+ CD38- naive	Inverse variance weighted	5	0.939072	0.142652	4.61E-11	0.659475	1.21867	2.557607	1
PHF5A	B cell	BAFF-R on IgD+ CD38- naive	Inverse variance weighted	5	-1.16859	0.172419	1.22E-11	-1.50653	-0.83065	0.310805	0
PHF5A	B cell	BAFF-R on IgD+ CD38- unsw mem	Inverse variance weighted	5	-1.3253	0.171528	1.11E-14	-1.6615	-0.98911	0.265723	0
PACSIN2	B cell	BAFF-R on IgD+ CD38- unsw mem	Wald ratio	1	-4.9208	0.49999	7.44E-23	-5.90078	-3.94082	0.007293	0
IMPG2	B cell	BAFF-R on IgD+ CD38dim	Inverse variance weighted	5	0.660422	0.103854	2.03E-10	0.456868	0.863976	1.935609	1
BAIAP2L2	B cell	BAFF-R on IgD+ CD38dim	Inverse variance weighted	4	0.878789	0.106361	1.43E-16	0.670321	1.087257	2.407982	1
BAIAP2L2	B cell	BAFF-R on IgD- CD27-	Inverse variance weighted	4	0.830445	0.105942	4.55E-15	0.622799	1.038092	2.294341	1
BAIAP2L2	B cell	BAFF-R on IgD- CD38-	Inverse variance weighted	4	0.877729	0.106216	1.41E-16	0.669545	1.085913	2.405431	1
PHF5A	B cell	BAFF-R on IgD- CD38-	Inverse variance weighted	5	-1.28228	0.178043	5.93E-13	-1.63125	-0.93332	0.277403	0
PHF5A	B cell	BAFF-R on IgD- CD38br	Inverse variance weighted	5	-0.70417	0.102719	7.12E-12	-0.9055	-0.50284	0.494519	
CSDC2	B cell	BAFF-R on IgD- CD38br	Inverse variance weighted	4	0.93287	0.143533	8.07E-11	0.651544	1.214195	2.541793	1
PHF5A	B cell	BAFF-R on unsw mem	Inverse variance weighted	5	-1.36536	0.194588	2.27E-12	-1.74675	-0.98397	0.255289	0
BAIAP2L2	B cell	BAFF-R on IgD+	Inverse variance weighted	3	0.848813	0.107762	3.36E-15	0.637599	1.060028	2.336872	1
MTFMT	B cell	CD19 on IgD+ CD38dim	Inverse variance weighted	26	-0.62258	0.059602	1.54E-25	-0.7394	-0.50576	0.53656	0
XPO6	B cell	CD19 on IgD- CD38dim	Wald ratio	1	3.038292	0.470565	1.07E-10	2.115985	3.960599	20.86956	8
XPO6	B cell	CD19 on sw mem	Wald ratio	1	2.979008	0.473158	3.05E-10	2.051618	3.906398	19.6683	7
ARL17A	B cell	CD20 on IgD+ CD38dim	Inverse variance weighted	3	0.406123	0.061762	4.85E-11	0.285069	0.527176	1.500987	1
PHF5A	B cell	CD20 on IgD- CD38dim	Inverse variance weighted	5	0.640367	0.102248	3.78E-10	0.43996	0.840773	1.897176	1
GAPDH	B cell	CD27 on CD24+ CD27+	Wald ratio	1	-2.05741	0.302038	9.64E-12	-2.6494	-1.46542	0.127784	0
CLEC4D	B cell	CD27 on CD24+ CD27+	Inverse variance weighted	18	1.016965	0.104666	2.57E-22	0.81182	1.222111	2.764792	2
IGHV1-58	B cell	CD27 on unsw mem	Inverse variance weighted	9	-0.83584	0.112238	9.55E-14	-1.05583	-0.61585	0.43351	0
GAPDH	B cell	CD27 on sw mem	Wald ratio	1	-2.55655	0.303498	3.65E-17	-3.1514	-1.96169	0.077572	0
CLEC4D	B cell	CD27 on sw mem	Inverse variance weighted	24	0.965348	0.110735	2.84E-18	0.748306	1.182389	2.6257	2
DCAF16	B cell	CD38 on IgD+ CD24-	Inverse variance weighted	4	0.553499	0.085427	9.22E-11	0.386061	0.720936	1.739328	1
IGHV6-1	B cell	IgD on IgD+ CD38dim	Inverse variance weighted	3	-0.91216	0.151266	1.64E-09	-1.20864	-0.61568	0.401654	0

IGHV3-43	B cell	IgD on IgD+ CD38dim	Inverse variance weighted	16	0.371116	0.046754	2.06E-15	0.279479	0.462753	1.449351	
IGHV3-49	B cell	IgD on IgD+ CD38dim	Inverse variance weighted	3	1.502759	0.231418	8.38E-11	1.049179	1.956338	4.49407	2
IGHVIII-67-4	B cell	IgD on IgD+ CD38dim	Inverse variance weighted	13	-0.25202	0.024109	1.41E-25	-0.29927	-0.20477	0.777229	0
EEF1DP7	B cell	IgD on IgD+ CD38dim	Inverse variance weighted	2	1.791484	0.263691	1.09E-11	1.27465	2.308318	5.998349	
DDX42	B cell	IgD on IgD+ CD38dim	Inverse variance weighted	2	-2.29752	0.337712	1.02E-11	-2.95944	-1.63561	0.100508	0
IGHV1-58	B cell	IgD on IgD+	Inverse variance weighted	8	-0.84586	0.128369	4.42E-11	-1.09746	-0.59425	0.42919	0
IGHVIII-67-4	B cell	IgD on IgD+	Inverse variance weighted	13	-0.21847	0.024171	1.59E-19	-0.26584	-0.17109	0.803749	0
IGHVIII-67-4	B cell	IgD on transitional	Inverse variance weighted	9	-0.23934	0.03141	2.54E-14	-0.3009	-0.17777	0.787148	
USP45	TBNK	CD3 on NKT	Inverse variance weighted	3	2.02346	0.255142	2.18E-15	1.523382	2.523538	7.56445	4
RPS3P5	TBNK	CD3 on NKT	Inverse variance weighted	3	-0.56928	0.071836	2.29E-15	-0.71008	-0.42848	0.565935	0
SMC2	Treg	CD3 on CD8br	Inverse variance weighted	7	0.398207	0.061539	9.75E-11	0.277591	0.518824	1.489153	1
KIAA1522	Treg	CD127 on CD45RA- CD4 not Treg	Inverse variance weighted	8	5.627012	0.84232	2.38E-11	3.976065	7.277959	277.8308	5
LOC101928517	Myeloid cell	CD33 on CD66b++ myeloid cell	Wald ratio	1	0.646583	0.096843	2.45E-11	0.456771	0.836395	1.909006	1
GTPBP10	cDC	FSC-A on granulocyte	Inverse variance weighted	13	0.701048	0.08586	3.22E-16	0.532761	0.869334	2.015864	
LINC00339	Monocyte	CD40 on CD14+ CD16- monocyte	Inverse variance weighted	12	4.515926	0.397669	6.93E-30	3.736494	5.295358	91.46223	4
FCGR2A	Monocyte	CD40 on CD14+ CD16- monocyte	Wald ratio	1	2.923068	0.414258	1.71E-12	2.111122	3.735013	18.59826	8
MB21D2	Monocyte	CCR2 on CD14- CD16+ monocyte	Inverse variance weighted	8	-0.19944	0.029728	1.96E-11	-0.2577	-0.14117	0.819192	0
ATRIP	cDC	CCR2 on CD62L+ myeloid DC	Inverse variance weighted	5	1.074693	0.170659	3.03E-10	0.740202	1.409185	2.929094	2
PPP2R5CP	cDC	CD80 on monocyte	Wald ratio	1	1.954845	0.230516	2.25E-17	1.503034	2.406657	7.062827	4
ZNF696	cDC	SSC-A on monocyte	Inverse variance weighted	19	1.276014	0.138236	2.69E-20	1.005071	1.546957	3.582332	2
HGH1	cDC	SSC-A on monocyte	Inverse variance weighted	2	-1.2362	0.165387	7.74E-14	-1.56036	-0.91204	0.290487	0
MTA3	Treg	CD45RA on resting Treg	Inverse variance weighted	4	-1.48232	0.22642	5.88E-11	-1.9261	-1.03853	0.227111	0

Table S12. Summary MR odds ratios for the associations between prioritized genes and immune cell traits in the mediation analyses.

gene	Panel	Trait	se	pval	lo_ci	up_ci	or	or_lci95	or_uci95
GAPDH	B cell	IgD- CD27- AC	0.36409	0.581993	-0.51319	0.914038	1.221919	0.598581	2.494375
KLRB1	B cell	IgD- CD38br %lymphocyte	0.075075	0.883132	-0.15818	0.136111	0.989025	0.853694	1.145809
HGH1	B cell	IgD- CD38dim %lymphocyte	1.233382	0.099394	5.418144	10.253	2528.984	225.4603	28367.55
HGH1	B cell	IgD- CD38dim %lymphocyte	0.156018	0.058051	-0.6015	0.010092	0.744008	0.547989	1.010143
HGH1	B cell	IgD- CD38dim %lymphocyte	0.667149	0.79906	-1.47744	1.13778	0.843806	0.22822	3.119834

HGH1	B cell	IgD- CD38dim %lymphocyte	0.224469	0.041961	-1.50097	-0.62105	0.346105	0.222914	0.537378
HGH1	B cell	IgD- CD38dim %lymphocyte	0.212222	0.05947	0.414985	1.246897	2.295479	1.514349	3.479531
RNLS	Treg	CD39+ resting Treg AC	0.079623	0.053979	-0.00263	0.309494	1.16583	0.997376	1.362736
RANBP17	Treg	CD39+ resting Treg % CD4 Treg	0.079527	0.829612	-0.17299	0.138759	0.983031	0.841149	1.148847
RNLS	Treg	CD39+ resting Treg % CD4 Treg	1.409173	0.572867	-3.88056	1.6434	0.326744	0.020639	5.172725
RNLS	Treg	CD39+ resting Treg % CD4 Treg	0.076893	0.248746	-0.06202	0.239398	1.09274	0.939863	1.270484
RNLS	Treg	CD39+ resting Treg % CD4 Treg	0.088138	0.488914	-0.11176	0.233745	1.062893	0.894263	1.263323
RNLS	Treg	CD39+ resting Treg % CD4 Treg	0.113045	0.508137	-0.13125	0.311882	1.094518	0.876995	1.365994
RNLS	Treg	CD39+ resting Treg % CD4 Treg	0.081017	0.284479	-0.04144	0.276145	1.124515	0.959406	1.318038
SEMA3C	Treg	CD39+ activated Treg AC	0.298227	0.155001	-1.00863	0.16042	0.654356	0.364719	1.174004
RPP30	Treg	CD39+ activated Treg AC	0.044356	0.034484	-0.18889	-0.01502	0.903069	0.827875	0.985094
RPP30	Treg	CD39+ activated Treg AC	0.030556	0.780592	-0.0684	0.051378	0.991525	0.933887	1.052721
RPP30	Treg	CD39+ activated Treg AC	0.021277	0.926682	-0.03974	0.043661	1.00196	0.961034	1.044628
RPP30	Treg	CD39+ activated Treg AC	0.055133	0.712794	-0.12868	0.087442	0.979593	0.879257	1.091379
RPP30	Treg	CD39+ activated Treg AC	0.041705	0.627014	-0.10236	0.061123	0.979593	0.902706	1.063029
PLSCR1	Treg	CD25hi CD45RA+ CD4 not Treg AC	0.135615	0.078273	-0.5046	0.027015	0.787579	0.603749	1.027383
LOC101928517	Myeloid cell	CD33dim HLA DR- AC	0.432466	0.481411	-1.17214	0.523123	0.722882	0.309703	1.68729
LOC101928517	Myeloid cell	CD33dim HLA DR- AC	0.047445	0.56929	-0.06599	0.119993	1.027369	0.936139	1.127489
LOC101928517	Myeloid cell	CD33dim HLA DR- AC	0.162346	0.38635	-0.17756	0.458831	1.151002	0.837307	1.582224
LOC101928517	Myeloid cell	CD33dim HLA DR- AC	0.08871	0.985971	-0.17549	0.172256	0.998385	0.839047	1.187982
LOC101928517	Myeloid cell	CD33dim HLA DR- AC	0.0353	0.416145	-0.03868	0.099692	1.030974	0.962054	1.104831
FAM114A2	B cell	Transitional AC	0.07928	0.978271	-0.15755	0.153229	0.997843	0.854236	1.165592
FCGR2A	Monocyte	CD14- CD16+ monocyte AC	0.127553	0.434179	-0.1456	0.354414	1.110055	0.864508	1.425345
FCGR2A	Monocyte	CD14- CD16+ monocyte AC	0.026552	0.020408	0.009526	0.113612	1.063504	1.009572	1.120317
FCGR2A	Monocyte	CD14- CD16+ monocyte AC	0.04563	0.092091	-0.01257	0.166296	1.079893	0.987506	1.180923
FCGR2A	Monocyte	CD14- CD16+ monocyte AC	0.036894	0.178307	-0.0189	0.125725	1.054865	0.981278	1.133971
FCGR2A	Monocyte	CD14- CD16+ monocyte AC	0.028492	0.073113	0.001204	0.112892	1.058707	1.001205	1.119511
P3H2	Monocyte	CD14+ CD16- monocyte AC	0.157264	0.783163	-0.26496	0.351516	1.044229	0.767238	1.42122

USP45	TBNK	CD8dim AC	0.216884	0.429657	-0.63806	0.212122	0.80818	0.528314	1.236299
USP45	TBNK	CD8dim AC	0.048242	0.33848	-0.04838	0.140732	1.047259	0.952773	1.151116
USP45	TBNK	CD8dim AC	0.118042	0.208573	-0.08292	0.379799	1.16002	0.920421	1.46199
USP45	TBNK	CD8dim AC	0.09469	0.625067	-0.13421	0.23698	1.05273	0.87441	1.267416
USP45	TBNK	CD8dim AC	0.048278	0.473743	-0.05518	0.134071	1.040235	0.946316	1.143474
RPS3P5	TBNK	CD8dim AC	0.973658	0.535665	-1.18646	2.630284	2.05837	0.305302	13.87771
RPS3P5	TBNK	CD8dim AC	0.121719	0.962462	-0.23284	0.244298	1.005745	0.79228	1.276724
RPS3P5	TBNK	CD8dim AC	0.454977	0.271171	-1.3924	0.391111	0.606141	0.248479	1.478623
RPS3P5	TBNK	CD8dim AC	0.184163	0.581776	-0.47431	0.24761	0.892838	0.622315	1.28096
RPS3P5	TBNK	CD8dim AC	0.113901	0.947714	-0.21514	0.231356	1.008144	0.806432	1.260308
RPS3P5	TBNK	CD8dim %T cell	0.567376	0.158569	-0.17233	2.051783	2.559283	0.841702	7.781767
RPS3P5	TBNK	CD8dim %T cell	0.104007	0.826689	-0.18108	0.226625	1.023034	0.834368	1.254359
RPS3P5	TBNK	CD8dim %T cell	0.347435	0.069291	-1.31209	0.049852	0.531995	0.269256	1.051115
RPS3P5	TBNK	CD8dim %T cell	0.172013	0.720211	-0.27256	0.401734	1.066721	0.761431	1.494414
RPS3P5	TBNK	CD8dim %T cell	0.093751	0.677102	-0.14274	0.224763	1.041863	0.866978	1.252025
RPS3P5	TBNK	CD8dim %leukocyte	0.577855	0.130668	-0.12128	2.143913	2.749221	0.885788	8.532761
RPS3P5	TBNK	CD8dim %leukocyte	0.099136	0.723628	-0.15925	0.229362	1.035678	0.852783	1.257797
RPS3P5	TBNK	CD8dim %leukocyte	0.336349	0.074909	-1.25829	0.060199	0.549336	0.28414	1.062048
RPS3P5	TBNK	CD8dim %leukocyte	0.146275	0.388875	-0.15234	0.421064	1.14381	0.8587	1.523582
RPS3P5	TBNK	CD8dim %leukocyte	0.096099	0.54733	-0.1276	0.249108	1.062639	0.880207	1.282881
PPP1R3D	TBNK	HLA DR+ CD4+ %lymphocyte	0.080435	0.594449	-0.11483	0.200475	1.043754	0.891519	1.221983
HGH1	TBNK	CD8br NKT %T cell	16.19155	0.375106	-55.959	7.511842	3.02E-11	4.98E-25	1829.581
HGH1	TBNK	CD8br NKT %T cell	0.170558	0.002945	0.172848	0.841435	1.660538	1.188685	2.319694
HGH1	TBNK	CD8br NKT %T cell	0.579599	0.494511	-0.74006	1.531969	1.485802	0.477085	4.627281
HGH1	TBNK	CD8br NKT %T cell	0.211659	0.039329	0.620686	1.450389	2.81662	1.860204	4.264773
HGH1	TBNK	CD8br NKT %T cell	0.240403	0.112809	-1.12491	-0.18253	0.520107	0.324682	0.833159
HGH1	TBNK	CD8br NKT %lymphocyte	13.3861	0.346024	-48.3903	4.083266	2.39E-10	9.65E-22	59.33898
HGH1	TBNK	CD8br NKT %lymphocyte	0.161503	0.002474	0.17224	0.805333	1.630337	1.187963	2.237441
HGH1	TBNK	CD8br NKT %lymphocyte	0.571481	0.498106	-0.73294	1.507266	1.472797	0.480494	4.514372
HGH1	TBNK	CD8br NKT %lymphocyte	0.218169	0.042582	0.59558	1.450804	2.782061	1.814082	4.266544
HGH1	TBNK	CD8br NKT %lymphocyte	0.260377	0.132774	-1.15169	-0.13101	0.526581	0.316102	0.877208
CFAP161	Treg	CD39+ CD4+ %CD4+	0.078107	0.283003	-0.06923	0.236946	1.087472	0.933108	1.267373
RPP30	Treg	CD39+ CD8br AC	0.396848	0.189062	-0.2005	1.355141	1.781257	0.81832	3.877308

RPP30	Treg	CD39+ CD8br AC	0.038204	0.140089	-0.01851	0.131247	1.057987	0.981659	1.140249
RPP30	Treg	CD39+ CD8br AC	0.097763	0.13473	-0.33784	0.045391	0.863964	0.71331	1.046437
RPP30	Treg	CD39+ CD8br AC	0.053291	0.260979	-0.03999	0.168908	1.066581	0.960797	1.184011
RPP30	Treg	CD39+ CD8br AC	0.035766	0.130806	-0.00989	0.130307	1.062055	0.990154	1.139178
LPCAT2	Treg	CD28+ CD45RA+ CD8br AC	0.098742	0.165992	-0.33031	0.056757	0.872165	0.718701	1.058399
PHF5A	B cell	BAFF-R on CD24+ CD27+	0.026629	0.558254	-0.03652	0.067869	1.015799	0.964141	1.070225
PHF5A	B cell	BAFF-R on CD24+ CD27+	0.010211	0.729077	-0.02355	0.016477	0.99647	0.976725	1.016613
PHF5A	B cell	BAFF-R on CD24+ CD27+	0.017032	0.077205	-0.06348	0.003285	0.97035	0.938491	1.00329
PHF5A	B cell	BAFF-R on CD24+ CD27+	0.023554	0.888997	-0.04947	0.042865	0.996704	0.951736	1.043797
PHF5A	B cell	BAFF-R on CD24+ CD27+	0.009638	0.733122	-0.02219	0.015589	0.996704	0.978053	1.015711
PHF5A	B cell	BAFF-R on IgD+ CD24+	0.026497	0.705801	-0.04188	0.061984	1.010101	0.958982	1.063945
PHF5A	B cell	BAFF-R on IgD+ CD24+	0.011027	0.858717	-0.02358	0.01965	0.998039	0.9767	1.019844
PHF5A	B cell	BAFF-R on IgD+ CD24+	0.017188	0.080831	-0.0637	0.00368	0.970438	0.93829	1.003687
PHF5A	B cell	BAFF-R on IgD+ CD24+	0.02344	0.867719	-0.04986	0.042022	0.996087	0.95136	1.042917
PHF5A	B cell	BAFF-R on IgD+ CD24+	0.009509	0.681564	-0.02256	0.014717	0.996087	0.977694	1.014826
BAIAP2L2	B cell	BAFF-R on IgD+ CD24-	0.106531	0.178063	-0.062	0.355602	1.158124	0.939884	1.42704
BAIAP2L2	B cell	BAFF-R on IgD+ CD24-	0.023542	0.448906	-0.02831	0.063968	1.017986	0.972082	1.066058
BAIAP2L2	B cell	BAFF-R on IgD+ CD24-	0.050814	0.103417	-0.01684	0.182348	1.086272	0.983297	1.200032
BAIAP2L2	B cell	BAFF-R on IgD+ CD24-	0.038842	0.845319	-0.06849	0.08377	1.007669	0.933802	1.087378
BAIAP2L2	B cell	BAFF-R on IgD+ CD24-	0.031188	0.985701	-0.06057	0.061693	1.000563	0.941232	1.063635
BAIAP2L2	B cell	BAFF-R on IgD+ CD38- naive	0.058974	0.616262	-0.0841	0.147078	1.031989	0.919338	1.158444
BAIAP2L2	B cell	BAFF-R on IgD+ CD38- naive	0.03065	0.763372	-0.05085	0.069302	1.00927	0.950424	1.07176
BAIAP2L2	B cell	BAFF-R on IgD+ CD38- naive	0.023441	0.789277	-0.03968	0.052209	1.006284	0.961097	1.053596
BAIAP2L2	B cell	BAFF-R on IgD+ CD38- naive	0.042161	0.508694	-0.05302	0.112253	1.030061	0.948363	1.118796
BAIAP2L2	B cell	BAFF-R on IgD+ CD38- naive	0.034968	0.893501	-0.06365	0.073422	1.004896	0.938329	1.076184
PHF5A	B cell	BAFF-R on IgD+ CD38- naive	0.026958	0.186489	-0.0167	0.088979	1.036802	0.983441	1.093057
PHF5A	B cell	BAFF-R on IgD+ CD38- naive	0.01026	0.946692	-0.0208	0.019424	0.999314	0.979419	1.019614
PHF5A	B cell	BAFF-R on IgD+ CD38- naive	0.01753	0.249203	-0.05456	0.014159	0.980003	0.946903	1.01426
PHF5A	B cell	BAFF-R on IgD+ CD38- naive	0.021077	0.922296	-0.04338	0.039244	0.997935	0.95755	1.040024
PHF5A	B cell	BAFF-R on IgD+ CD38- naive	0.008671	0.741702	-0.01412	0.019869	1.002879	0.985979	1.020067
PHF5A	B cell	BAFF-R on IgD+ CD38- unsw mem	0.027409	0.382812	-0.02957	0.077872	1.024445	0.970863	1.080984
PHF5A	B cell	BAFF-R on IgD+ CD38- unsw mem	0.010065	0.708424	-0.02349	0.015963	0.996243	0.976783	1.016091
PHF5A	B cell	BAFF-R on IgD+ CD38- unsw mem	0.017495	0.114873	-0.06187	0.006706	0.972793	0.940002	1.006729

PHF5A	B cell	BAFF-R on IgD+ CD38- unsw mem	0.021637	0.854699	-0.04639	0.038424	0.996024	0.954668	1.039172
PHF5A	B cell	BAFF-R on IgD+ CD38- unsw mem	0.008826	0.65378	-0.02128	0.013315	0.996024	0.978941	1.013404
PACSIN2	B cell	BAFF-R on IgD+ CD38- unsw mem	0.036997	0.076516	-0.13962	0.005405	0.935094	0.869687	1.00542
PACSIN2	B cell	BAFF-R on IgD+ CD38- unsw mem	0.012117	0.479822	-0.01519	0.032311	1.008598	0.984927	1.032838
PACSIN2	B cell	BAFF-R on IgD+ CD38- unsw mem	0.014783	0.092883	-0.05382	0.004134	0.975464	0.947606	1.004142
PACSIN2	B cell	BAFF-R on IgD+ CD38- unsw mem	0.020856	0.209288	-0.01431	0.067444	1.026922	0.98579	1.069771
PACSIN2	B cell	BAFF-R on IgD+ CD38- unsw mem	0.013532	0.251384	-0.0108	0.042248	1.015848	0.989258	1.043153
IMPG2	B cell	BAFF-R on IgD+ CD38dim	0.138899	0.478566	-0.37067	0.173816	0.906264	0.690274	1.189837
BAIAP2L2	B cell	BAFF-R on IgD+ CD38dim	0.109193	0.024598	0.043973	0.47201	1.294328	1.044954	1.603214
BAIAP2L2	B cell	BAFF-R on IgD+ CD38dim	0.022733	0.599972	-0.03263	0.056479	1.011993	0.967892	1.058104
BAIAP2L2	B cell	BAFF-R on IgD+ CD38dim	0.051172	0.074556	-0.00905	0.191546	1.095542	0.990993	1.21112
BAIAP2L2	B cell	BAFF-R on IgD+ CD38dim	0.034947	0.419753	-0.03993	0.097062	1.028977	0.960856	1.101928
BAIAP2L2	B cell	BAFF-R on IgD+ CD38dim	0.029573	0.937937	-0.05564	0.060284	1.002323	0.945877	1.062138
BAIAP2L2	B cell	BAFF-R on IgD- CD27-	0.123736	0.361818	-0.12767	0.357381	1.121714	0.880148	1.42958
BAIAP2L2	B cell	BAFF-R on IgD- CD27-	0.024254	0.097621	-0.00736	0.087715	1.040995	0.992666	1.091677
BAIAP2L2	B cell	BAFF-R on IgD- CD27-	0.059225	0.058943	-0.00423	0.227936	1.11835	0.995781	1.256005
BAIAP2L2	B cell	BAFF-R on IgD- CD27-	0.033259	0.223221	-0.02372	0.106652	1.042336	0.976555	1.112547
BAIAP2L2	B cell	BAFF-R on IgD- CD27-	0.030467	0.278039	-0.02599	0.093443	1.034303	0.974348	1.097947
BAIAP2L2	B cell	BAFF-R on IgD- CD38-	0.148582	0.747315	-0.2428	0.339646	1.049617	0.784431	1.404451
BAIAP2L2	B cell	BAFF-R on IgD- CD38-	0.025992	0.532827	-0.03473	0.067156	1.016343	0.965863	1.069463
BAIAP2L2	B cell	BAFF-R on IgD- CD38-	0.065449	0.058254	-0.00433	0.252226	1.131955	0.995675	1.286886
BAIAP2L2	B cell	BAFF-R on IgD- CD38-	0.035739	0.405453	-0.03981	0.100292	1.030706	0.960977	1.105494
BAIAP2L2	B cell	BAFF-R on IgD- CD38-	0.027303	0.828762	-0.04755	0.059482	1.005985	0.953566	1.061286
PHF5A	B cell	BAFF-R on IgD- CD38-	0.026226	0.553567	-0.03578	0.06703	1.015749	0.964855	1.069328
PHF5A	B cell	BAFF-R on IgD- CD38-	0.010468	0.656497	-0.02517	0.015861	0.995355	0.975142	1.015987
PHF5A	B cell	BAFF-R on IgD- CD38-	0.017036	0.083301	-0.06289	0.003887	0.970927	0.939042	1.003894
PHF5A	B cell	BAFF-R on IgD- CD38-	0.024599	0.742663	-0.05633	0.0401	0.991919	0.945229	1.040915
PHF5A	B cell	BAFF-R on IgD- CD38-	0.009536	0.629558	-0.02331	0.014067	0.995387	0.976955	1.014167
PHF5A	B cell	BAFF-R on IgD- CD38br	0.057413	0.185303	-0.19161	0.033454	0.92397	0.825632	1.03402
PHF5A	B cell	BAFF-R on IgD- CD38br	0.029205	0.779778	-0.06541	0.049075	0.991867	0.936686	1.0503
PHF5A	B cell	BAFF-R on IgD- CD38br	0.029741	0.94851	-0.06021	0.056371	0.998081	0.941564	1.057991
PHF5A	B cell	BAFF-R on IgD- CD38br	0.054385	0.936681	-0.10222	0.110973	1.004388	0.902834	1.117365
PHF5A	B cell	BAFF-R on IgD- CD38br	0.026153	0.736534	-0.06019	0.042331	0.991111	0.941586	1.04324

CSDC2	B cell	BAFF-R on IgD- CD38br	0.031689	0.00453	0.037127	0.161349	1.104329	1.037825	1.175095
CSDC2	B cell	BAFF-R on IgD- CD38br	0.019311	0.007069	0.014166	0.089866	1.053393	1.014267	1.094027
CSDC2	B cell	BAFF-R on IgD- CD38br	0.019517	0.4785	-0.02442	0.052084	1.013928	0.975875	1.053465
CSDC2	B cell	BAFF-R on IgD- CD38br	0.042903	0.135404	-0.01789	0.150286	1.068437	0.982267	1.162167
CSDC2	B cell	BAFF-R on IgD- CD38br	0.019159	0.027349	0.007351	0.082455	1.045926	1.007378	1.08595
PHF5A	B cell	BAFF-R on unsw mem	0.025174	0.56744	-0.03487	0.063814	1.014579	0.965734	1.065894
PHF5A	B cell	BAFF-R on unsw mem	0.010524	0.775026	-0.02364	0.017619	0.996997	0.976642	1.017776
PHF5A	B cell	BAFF-R on unsw mem	0.01674	0.067943	-0.06337	0.002253	0.969905	0.938599	1.002256
PHF5A	B cell	BAFF-R on unsw mem	0.029112	0.896336	-0.06087	0.053252	0.996199	0.940947	1.054695
PHF5A	B cell	BAFF-R on unsw mem	0.008852	0.668483	-0.02116	0.01354	0.996199	0.979065	1.013632
BAIAP2L2	B cell	BAFF-R on IgD+	0.125807	0.204534	-0.08286	0.410307	1.177891	0.920484	1.507281
BAIAP2L2	B cell	BAFF-R on IgD+	0.024346	0.103287	-0.00806	0.087379	1.040459	0.991977	1.09131
BAIAP2L2	B cell	BAFF-R on IgD+	0.060197	0.054683	-0.00232	0.233647	1.122615	0.997678	1.263198
BAIAP2L2	B cell	BAFF-R on IgD+	0.036342	0.300833	-0.03289	0.109566	1.03908	0.967641	1.115793
BAIAP2L2	B cell	BAFF-R on IgD+	0.02993	0.344819	-0.02988	0.087442	1.029197	0.970558	1.091379
MTFMT	B cell	CD19 on IgD+ CD38dim	0.064886	0.294915	-0.19514	0.059215	0.934296	0.822721	1.061003
XPO6	B cell	CD19 on IgD- CD38dim	1.158392	0.820156	-1.98319	2.557704	1.332765	0.137629	12.90615
XPO6	B cell	CD19 on IgD- CD38dim	0.046691	3.45E-10	0.201567	0.384596	1.340552	1.223318	1.46902
XPO6	B cell	CD19 on IgD- CD38dim	0.162929	0.400946	-0.18249	0.456192	1.146656	0.833191	1.578053
XPO6	B cell	CD19 on IgD- CD38dim	0.048517	0.003345	0.208036	0.398221	1.354088	1.231257	1.489173
XPO6	B cell	CD19 on IgD- CD38dim	0.042819	0.002101	0.219204	0.387053	1.354088	1.245085	1.472634
XPO6	B cell	CD19 on sw mem	1.39311	0.903498	-2.54691	2.914084	1.201521	0.078323	18.43193
XPO6	B cell	CD19 on sw mem	0.047055	2.59E-09	0.188013	0.372469	1.323449	1.206849	1.451313
XPO6	B cell	CD19 on sw mem	0.15839	0.392596	-0.17503	0.445854	1.145007	0.839429	1.561824
XPO6	B cell	CD19 on sw mem	0.047372	0.003397	0.201894	0.387591	1.34278	1.223718	1.473427
XPO6	B cell	CD19 on sw mem	0.048197	0.00362	0.200276	0.389209	1.34278	1.22174	1.475813
ARL17A	B cell	CD20 on IgD+ CD38dim	0.325124	0.056232	-0.01651	1.25798	1.860298	0.983629	3.518306
PHF5A	B cell	CD20 on IgD- CD38dim	0.131721	0.473622	-0.35257	0.163782	0.909927	0.702883	1.177958
GAPDH	B cell	CD27 on CD24+ CD27+	0.715242	0.574134	-0.92578	1.877972	1.60978	0.396223	6.540228
GAPDH	B cell	CD27 on CD24+ CD27+	0.04669	0.007272	-0.21683	-0.03381	0.882214	0.805065	0.966756
GAPDH	B cell	CD27 on CD24+ CD27+	0.138761	0.08391	-0.51181	0.032132	0.786755	0.59941	1.032654
GAPDH	B cell	CD27 on CD24+ CD27+	0.194577	0.212043	-0.68897	0.073769	0.735208	0.502091	1.076558
GAPDH	B cell	CD27 on CD24+ CD27+	0.045993	0.187816	-0.16831	0.011988	0.924818	0.845096	1.012061

CLEC4D	B cell	CD27 on CD24+ CD27+	0.855739	0.217915	-2.77779	0.576703	0.33269	0.062176	1.780159
CLEC4D	B cell	CD27 on CD24+ CD27+	0.022885	0.494607	-0.02922	0.060485	1.015753	0.971199	1.062352
CLEC4D	B cell	CD27 on CD24+ CD27+	0.017987	0.120469	-0.00732	0.063187	1.028325	0.992702	1.065226
CLEC4D	B cell	CD27 on CD24+ CD27+	0.038575	0.724029	-0.06174	0.089469	1.013959	0.940123	1.093594
CLEC4D	B cell	CD27 on CD24+ CD27+	0.040093	0.73955	-0.06502	0.092144	1.013655	0.93705	1.096523
IGHV1-58	B cell	CD27 on unsw mem	0.163181	0.306025	-0.1528	0.486867	1.181792	0.858299	1.62721
GAPDH	B cell	CD27 on sw mem	0.33421	0.595255	-0.85305	0.457059	0.820375	0.426115	1.579422
GAPDH	B cell	CD27 on sw mem	0.042243	0.040333	-0.16941	-0.00382	0.917034	0.844166	0.996192
GAPDH	B cell	CD27 on sw mem	0.099321	0.072226	-0.37322	0.016121	0.836483	0.688514	1.016251
GAPDH	B cell	CD27 on sw mem	0.061924	0.171691	-0.22434	0.018405	0.902158	0.799047	1.018576
GAPDH	B cell	CD27 on sw mem	0.033785	0.166941	-0.1232	0.009234	0.944609	0.884085	1.009277
CLEC4D	B cell	CD27 on sw mem	0.28201	0.298904	-0.19932	0.906164	1.423935	0.819291	2.474812
CLEC4D	B cell	CD27 on sw mem	0.042031	0.213791	-0.03013	0.134635	1.053643	0.970322	1.144119
CLEC4D	B cell	CD27 on sw mem	0.031483	0.161119	-0.01759	0.105826	1.045106	0.982565	1.111628
CLEC4D	B cell	CD27 on sw mem	0.058605	0.423179	-0.06264	0.167096	1.053618	0.939285	1.181868
CLEC4D	B cell	CD27 on sw mem	0.055318	0.398545	-0.05619	0.160654	1.053618	0.945355	1.174279
DCAF16	B cell	CD38 on IgD+ CD24-	0.163125	0.666323	-0.24938	0.390063	1.072872	0.77928	1.477075
IGHV6-1	B cell	IgD on IgD+ CD38dim	0.260081	0.934419	-0.48767	0.531842	1.022329	0.614053	1.702065
IGHV6-1	B cell	IgD on IgD+ CD38dim	0.095946	0.373839	-0.27338	0.102729	0.918213	0.760804	1.108191
IGHV6-1	B cell	IgD on IgD+ CD38dim	0.076735	0.068917	-0.28998	0.010822	0.869725	0.748279	1.010881
IGHV6-1	B cell	IgD on IgD+ CD38dim	0.134445	0.550903	-0.34681	0.180214	0.920077	0.70694	1.197473
IGHV6-1	B cell	IgD on IgD+ CD38dim	0.111991	0.461563	-0.30563	0.133375	0.917477	0.736659	1.142678
IGHV3-43	B cell	IgD on IgD+ CD38dim	1.383866	0.378711	-4.08082	1.34393	0.254502	0.016894	3.834083
IGHV3-43	B cell	IgD on IgD+ CD38dim	0.183266	0.151729	-0.0965	0.621906	1.300441	0.908011	1.862474
IGHV3-43	B cell	IgD on IgD+ CD38dim	0.384589	0.170188	-0.22629	1.281298	1.694695	0.797485	3.601309
IGHV3-43	B cell	IgD on IgD+ CD38dim	0.182131	0.2514	-0.12084	0.593118	1.266352	0.886178	1.809622
IGHV3-43	B cell	IgD on IgD+ CD38dim	0.178843	0.208473	-0.09237	0.608696	1.29455	0.911768	1.838032
IGHV3-49	B cell	IgD on IgD+ CD38dim	0.093486	0.132693	-0.33972	0.02675	0.855146	0.711973	1.027111
IGHV3-49	B cell	IgD on IgD+ CD38dim	0.037787	0.456309	-0.10221	0.045914	0.972243	0.902838	1.046984
IGHV3-49	B cell	IgD on IgD+ CD38dim	0.030991	0.633175	-0.07553	0.045952	0.985318	0.927249	1.047024
IGHV3-49	B cell	IgD on IgD+ CD38dim	0.064535	0.971309	-0.1241	0.128875	1.002389	0.88329	1.137548
IGHV3-49	B cell	IgD on IgD+ CD38dim	0.03561	0.320165	-0.10726	0.032329	0.963226	0.898289	1.032858
IGHVIII-67-4	B cell	IgD on IgD+ CD38dim	0.717075	0.585522	-0.85914	1.95179	1.726892	0.423525	7.041277

IGHVIII-67-4	B cell	IgD on IgD+ CD38dim	0.266896	0.996737	-0.52421	0.522025	0.998909	0.592024	1.685438
IGHVIII-67-4	B cell	IgD on IgD+ CD38dim	0.22512	0.642725	-0.54567	0.336804	0.900836	0.579455	1.400465
IGHVIII-67-4	B cell	IgD on IgD+ CD38dim	0.320831	0.988666	-0.62369	0.633971	1.005156	0.535966	1.885081
IGHVIII-67-4	B cell	IgD on IgD+ CD38dim	0.288392	0.987391	-0.56011	0.570392	1.005156	0.571149	1.76896
EEF1DP7	B cell	IgD on IgD+ CD38dim	1.21227	0.207787	-0.43575	4.31635	6.960845	0.646781	74.91466
EEF1DP7	B cell	IgD on IgD+ CD38dim	0.083228	0.000537	0.124959	0.451214	1.333872	1.133101	1.570217
EEF1DP7	B cell	IgD on IgD+ CD38dim	0.147224	0.117934	-0.05837	0.518743	1.258832	0.943297	1.679914
EEF1DP7	B cell	IgD on IgD+ CD38dim	0.09777	0.018579	0.183071	0.566327	1.454554	1.200899	1.761785
EEF1DP7	B cell	IgD on IgD+ CD38dim	0.077847	0.00824	0.226275	0.531435	1.460611	1.25392	1.701372
DDX42	B cell	IgD on IgD+ CD38dim	1.191053	0.350714	-3.64772	1.021208	0.268943	0.02605	2.776547
DDX42	B cell	IgD on IgD+ CD38dim	0.052586	8.67E-05	-0.30948	-0.10334	0.813498	0.733828	0.901817
DDX42	B cell	IgD on IgD+ CD38dim	0.129567	0.372967	-0.36939	0.138517	0.890978	0.691157	1.148569
DDX42	B cell	IgD on IgD+ CD38dim	0.062433	0.013186	-0.38754	-0.14281	0.767072	0.678723	0.866922
DDX42	B cell	IgD on IgD+ CD38dim	0.057881	0.014146	-0.35423	-0.12733	0.786014	0.701716	0.880439
IGHV1-58	B cell	IgD on IgD+	0.097374	0.140162	-0.03703	0.344673	1.166281	0.963645	1.411528
IGHV1-58	B cell	IgD on IgD+	0.039254	0.66	-0.05967	0.094206	1.017418	0.942076	1.098786
IGHV1-58	B cell	IgD on IgD+	0.031212	0.626551	-0.04599	0.076364	1.015303	0.955053	1.079355
IGHV1-58	B cell	IgD on IgD+	0.064025	0.550575	-0.08625	0.164724	1.040015	0.917361	1.179068
IGHV1-58	B cell	IgD on IgD+	0.034227	0.449007	-0.04037	0.093802	1.027078	0.960438	1.098342
IGHVIII-67-4	B cell	IgD on IgD+	0.783411	0.585486	-0.93855	2.13242	1.816541	0.391194	8.435259
IGHVIII-67-4	B cell	IgD on IgD+	0.29512	0.995956	-0.57993	0.576938	0.998505	0.559937	1.780579
IGHVIII-67-4	B cell	IgD on IgD+	0.236991	0.634561	-0.57715	0.351856	0.893467	0.561498	1.421704
IGHVIII-67-4	B cell	IgD on IgD+	0.321796	0.989831	-0.62609	0.635347	1.004639	0.534678	1.887678
IGHVIII-67-4	B cell	IgD on IgD+	0.326872	0.989989	-0.63604	0.645296	1.004639	0.529384	1.906552
IGHVIII-67-4	B cell	IgD on transitional	9.806699	0.883065	-21.043	17.39929	0.161728	7.26E-10	36009359
IGHVIII-67-4	B cell	IgD on transitional	0.151325	0.267298	-0.46446	0.128731	0.845468	0.628473	1.137384
IGHVIII-67-4	B cell	IgD on transitional	0.133553	0.175896	-0.44253	0.081	0.834632	0.642411	1.084371
IGHVIII-67-4	B cell	IgD on transitional	0.217779	0.940564	-0.40851	0.445185	1.018507	0.664641	1.560778
IGHVIII-67-4	B cell	IgD on transitional	0.153893	0.261487	-0.54002	0.063246	0.787899	0.582739	1.065288
USP45	TBNK	CD3 on NKT	0.119894	0.122204	-0.54569	-0.07571	0.732936	0.579442	0.927089
USP45	TBNK	CD3 on NKT	0.052525	0.100392	-0.01665	0.189247	1.09013	0.983485	1.208339
USP45	TBNK	CD3 on NKT	0.123555	0.087455	-0.03102	0.453321	1.235101	0.969461	1.57353
USP45	TBNK	CD3 on NKT	0.182896	0.256892	-0.10302	0.613929	1.291047	0.902107	1.847676

USP45	TBNK	CD3 on NKT	0.047099	0.407694	-0.04708	0.137548	1.046273	0.954012	1.147457
RPS3P5	TBNK	CD3 on NKT	0.729094	0.231165	-0.18929	2.668757	3.454687	0.827544	14.42203
RPS3P5	TBNK	CD3 on NKT	0.125731	0.695121	-0.19716	0.295709	1.05051	0.821061	1.34408
RPS3P5	TBNK	CD3 on NKT	0.459257	0.192078	-1.49922	0.301063	0.549317	0.223303	1.351295
RPS3P5	TBNK	CD3 on NKT	0.213566	0.972061	-0.41047	0.42671	1.008153	0.663338	1.532208
RPS3P5	TBNK	CD3 on NKT	0.113526	0.634563	-0.16266	0.282361	1.061677	0.849878	1.326258
SMC2	Treg	CD3 on CD8br	0.059606	0.251051	-0.04841	0.185244	1.07081	0.952741	1.203512
KIAA1522	Treg	CD127 on CD45RA- CD4 not Treg	0.073443	0.074035	-0.01275	0.275146	1.140194	0.987331	1.316724
LOC101928517	Myeloid cell	CD33 on CD66b++ myeloid cell	0.088571	0.943709	-0.17993	0.167266	0.993687	0.835327	1.182069
LOC101928517	Myeloid cell	CD33 on CD66b++ myeloid cell	0.021442	0.501772	-0.05643	0.027624	0.985701	0.945134	1.028009
LOC101928517	Myeloid cell	CD33 on CD66b++ myeloid cell	0.044544	0.422458	-0.05157	0.123038	1.036378	0.949733	1.130927
LOC101928517	Myeloid cell	CD33 on CD66b++ myeloid cell	0.037754	0.809682	-0.08321	0.06479	0.990834	0.920162	1.066935
LOC101928517	Myeloid cell	CD33 on CD66b++ myeloid cell	0.022438	0.81871	-0.04919	0.038771	0.994806	0.952004	1.039532
GTPBP10	cDC	FSC-A on granulocyte	0.072522	0.305096	-0.06777	0.216518	1.077212	0.934479	1.241746
LINC00339	Monocyte	CD40 on CD14+ CD16- monocyte	0.124206	0.619188	-0.18171	0.305175	1.063676	0.83384	1.356862
FCGR2A	Monocyte	CD40 on CD14+ CD16- monocyte	0.095383	0.726549	-0.2215	0.152398	0.966038	0.801313	1.164624
FCGR2A	Monocyte	CD40 on CD14+ CD16- monocyte	0.017036	0.281973	-0.05172	0.015062	0.981838	0.949595	1.015176
FCGR2A	Monocyte	CD40 on CD14+ CD16- monocyte	0.042245	0.579306	-0.05938	0.106221	1.023697	0.942349	1.112068
FCGR2A	Monocyte	CD40 on CD14+ CD16- monocyte	0.039934	0.558939	-0.1025	0.054036	0.976057	0.902574	1.055522
FCGR2A	Monocyte	CD40 on CD14+ CD16- monocyte	0.017606	0.201957	-0.05874	0.010274	0.976057	0.942949	1.010327
MB21D2	Monocyte	CCR2 on CD14- CD16+ monocyte	0.076441	0.361556	-0.08008	0.219569	1.072235	0.923043	1.245539
ATRIP	cDC	CCR2 on CD62L+ myeloid DC	63.26822	0.553839	-81.9589	166.0525	1.82E+18	2.55E-36	1.31E+72
ATRIP	cDC	CCR2 on CD62L+ myeloid DC	0.100037	0.374261	-0.10719	0.284958	1.092955	0.898357	1.329706
ATRIP	cDC	CCR2 on CD62L+ myeloid DC	0.084004	0.494162	-0.10721	0.222081	1.059115	0.898334	1.248673
ATRIP	cDC	CCR2 on CD62L+ myeloid DC	0.12392	0.499552	-0.15099	0.334772	1.096243	0.859853	1.397621
ATRIP	cDC	CCR2 on CD62L+ myeloid DC	0.130927	0.517435	-0.16378	0.349449	1.097279	0.848926	1.418286
PPP2R5CP	cDC	CD80 on monocyte	0.135683	0.187204	-0.07615	0.455723	1.208989	0.926673	1.577314
PPP2R5CP	cDC	CD80 on monocyte	0.039929	0.022093	0.013127	0.16965	1.095695	1.013214	1.184891

PPP2R5CP	cDC	CD80 on monocyte	0.075025	0.230417	-0.05707	0.237026	1.094149	0.944525	1.267474
PPP2R5CP	cDC	CD80 on monocyte	0.103648	0.011865	0.099844	0.506146	1.353908	1.104999	1.658886
PPP2R5CP	cDC	CD80 on monocyte	0.041278	0.035874	0.015695	0.177506	1.10142	1.015819	1.194235
ZNF696	cDC	SSC-A on monocyte	0.851979	0.143188	0.332255	3.672013	7.404839	1.394108	39.33099
ZNF696	cDC	SSC-A on monocyte	0.048285	0.057338	-0.18642	0.002862	0.912309	0.829928	1.002866
ZNF696	cDC	SSC-A on monocyte	0.081551	0.623917	-0.19982	0.119855	0.960804	0.818874	1.127333
ZNF696	cDC	SSC-A on monocyte	0.059089	0.135721	-0.23559	-0.00397	0.887116	0.790101	0.996042
ZNF696	cDC	SSC-A on monocyte	0.058812	0.185611	-0.2159	0.014646	0.904271	0.805818	1.014754
HGH1	cDC	SSC-A on monocyte	1.259122	0.065029	9.815751	14.75151	216128.6	18320.04	2549754
HGH1	cDC	SSC-A on monocyte	0.129695	0.000109	-0.756	-0.2476	0.60544	0.46954	0.780674
HGH1	cDC	SSC-A on monocyte	0.529466	0.710684	-1.23415	0.841355	0.821684	0.291081	2.319509
HGH1	cDC	SSC-A on monocyte	0.14339	0.029992	-1.09027	-0.52818	0.445203	0.336126	0.589676
HGH1	cDC	SSC-A on monocyte	0.153497	0.034148	-1.11008	-0.50837	0.445203	0.329533	0.601474
MTA3	Treg	CD45RA on resting Treg	0.052767	0.140696	-0.02569	0.181159	1.080837	0.974641	1.198605

Table S13. Colocalization posterior probabilities (PPH0–PPH4) for blood eQTL and immune cell trait signals at loci of the prioritized genes.

gene	Panel	Trait	PPH0	PPH1	PPH2	PPH3	PPH4
SEMA3C	Treg	CD39+ activated Treg AC	2.49E-12	0.004685	5.28E-13	0	0.995315
RPP30	Treg	CD39+ activated Treg AC	1.02E-05	0.028547	5.39E-06	0.01406	0.957377
PLSCR1	Treg	CD25hi CD45RA+ CD4 not Treg AC	2.47E-07	0.118688	3.50E-09	0.000803	0.880509
FAM114A2	B cell	Transitional AC	1.11E-10	0.042766	3.19E-12	0.000272	0.956962
RPP30	Treg	CD39+ CD8br AC	0.000107	0.279264	2.76E-07	0	0.720629
BAIAP2L2	B cell	BAFF-R on IgD+ CD38dim	4.60E-71	1.63E-05	2.81E-69	0	0.999984
CLEC4D	B cell	CD27 on sw mem	6.14E-10	0.311451	3.15E-12	0.000909	0.68764

IGHV3-49	B cell	IgD on IgD+ CD38dim	0.026042	0.024254	0.002218	0.001119	0.946367
IGHV3-43	B cell	IgD on IgD+ CD38dim	8.90E-35	0.082537	2.78E-36	0.001661	0.915802

Figure S1. SMR plots for eight prioritized genes whose blood expression is associated with neuroblastoma risk. For each gene, the upper panel shows the relationship between cis-eQTL effect sizes and GWAS effect sizes, with the top cis-eQTL indicated by a red triangle and other cis-eQTLs in blue, and the lower panel displays the regional cis-eQTL association signals (–log10 P) together with nearby genes along the chromosome. Panels A–B, FAM182B; C–D, BTNL2; E–F, CPNE1; G–H, DNAJC9; I–J, EP400NL; K–L, NBPF3; M–N, TTC18; O–P, ZNF559.

A

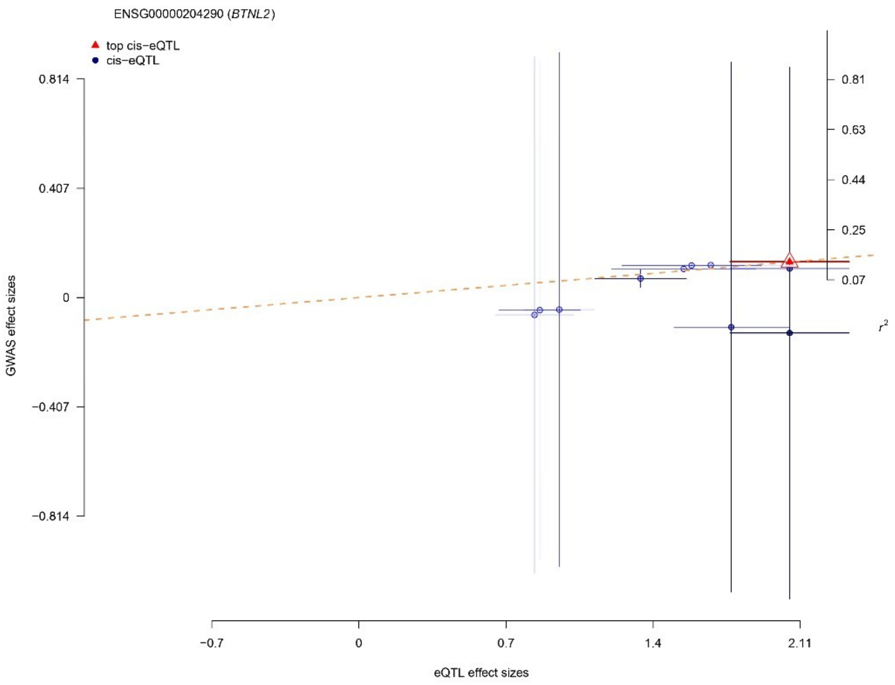

ENSG00000204290 (*BTNL2*)
top cis−eQTL
cis−eQTL
0.814
0.407
0
−0.407
−0.814
GWAS effect sizes
0.81
0.63
0.44
0.25
0.07
r^2
−0.7
0
0.7
1.4
2.11
eQTL effect sizes

B

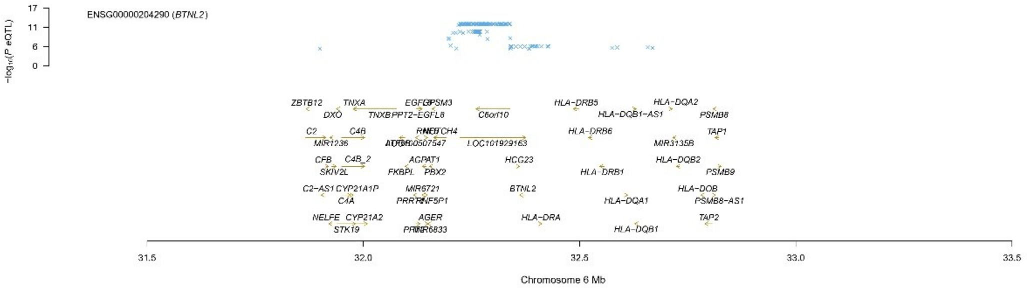
ENSG00000204290 (BTNL2)
−log10(P eQTL)
Chromosome 6 Mb
31.5
32.0
32.5
33.0
33.5

C

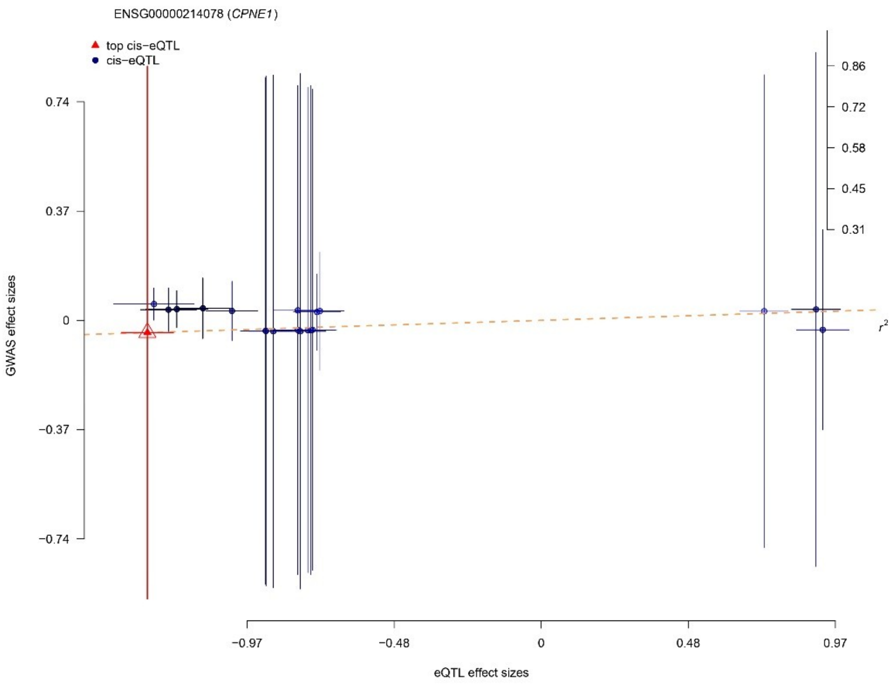

ENSG00000214078 (*CPNE1*)
top cis−eQTL
cis−eQTL
GWAS effect sizes
0.74
0.37
0
−0.37
−0.74
0.86
0.72
0.58
0.45
0.31
r2
−0.97
−0.48
0
0.48
0.97
eQTL effect sizes

D

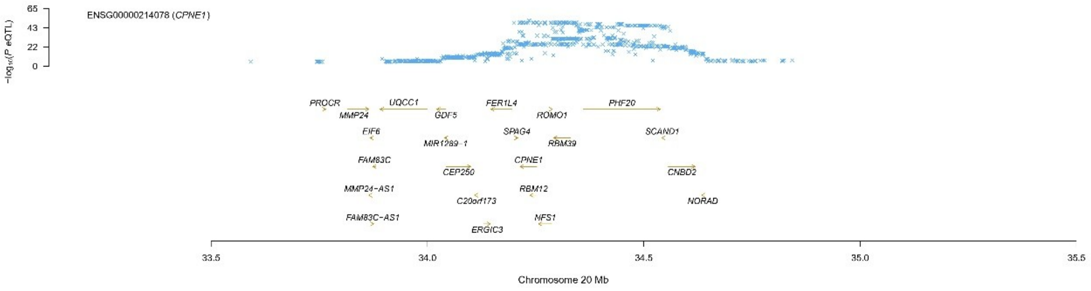
ENSG00000214078 (CPNE1)
65
43
22
0
−log10(P eQTL)
PROCR
MMP24
UQCC1
GDF5
FER1L4
ROMO1
PHF20
EIF6
MIR1289−1
SPAG4
RBM39
SCAND1
FAM83C
CEP250
CPNE1
CNBD2
MMP24−AS1
C20orf173
RBM12
NORAD
FAM83C−AS1
ERGIC3
NFS1
33.5
34.0
34.5
35.0
35.5
Chromosome 20 Mb

E

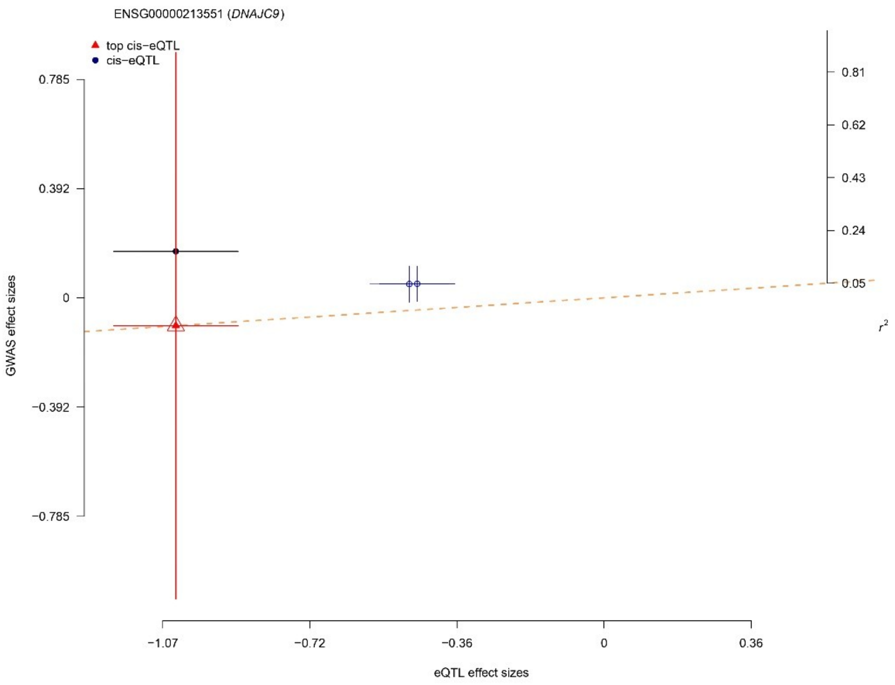

ENSG00000213551 (*DNAJC9*)
top cis−eQTL
cis−eQTL
0.785
0.392
0
−0.392
−0.785
GWAS effect sizes
0.81
0.62
0.43
0.24
0.05
r^2
−1.07
−0.72
−0.36
0
0.36
eQTL effect sizes

F

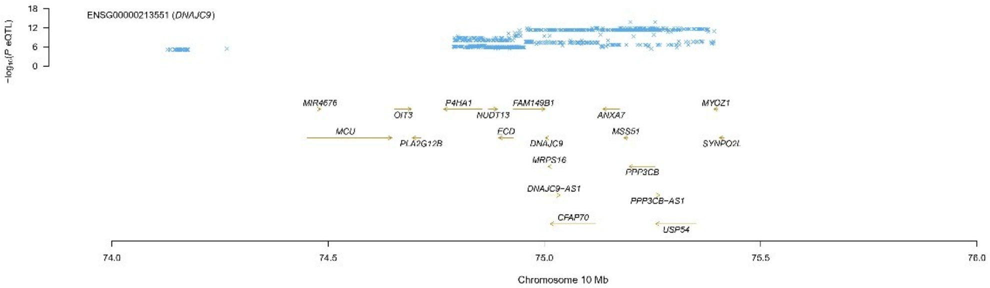

18
12
6
0
−log10(P eQTL)
ENSG00000213551 (DNAJC9)
MIR4676
OIT3
P4HA1
NUDT13
FAM149B1
ANXA7
MYOZ1
MCU
PLA2G12B
FCD
DNAJC9
MSS51
SYNPO2L
MRPS16
PPP3CB
DNAJC9−AS1
PPP3CB−AS1
CFAP70
USP54
74.0
74.5
75.0
75.5
76.0
Chromosome 10 Mb

G

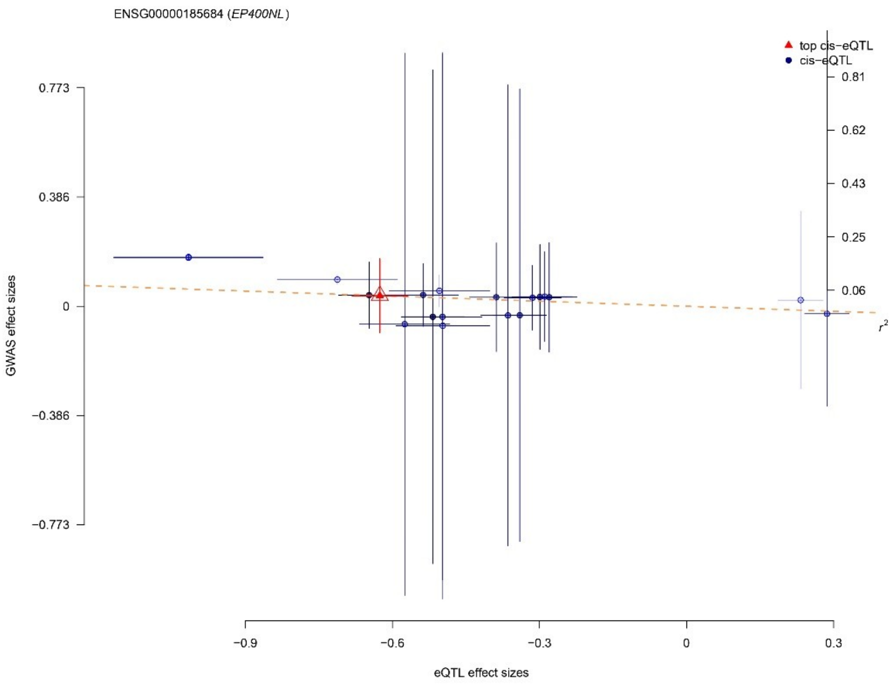
ENSG00000185684 (*EP400NL*)
top cis−eQTL
cis−eQTL
0.773
0.386
0
−0.386
−0.773
GWAS effect sizes
0.81
0.62
0.43
0.25
0.06
r^2
−0.9
−0.6
−0.3
0
0.3
eQTL effect sizes

H

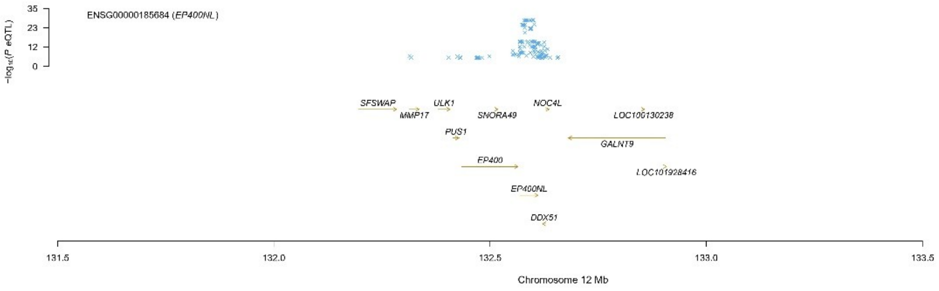
ENSG00000185684 (EP400NL)
35
23
12
0
−log10(P eQTL)
SFSWAP
MMP17
ULK1
SNORA49
NOC4L
LOC100130238
PUS1
GALNT9
EP400
LOC101928416
EP400NL
DDX51
131.5
132.0
132.5
133.0
133.5
Chromosome 12 Mb

I

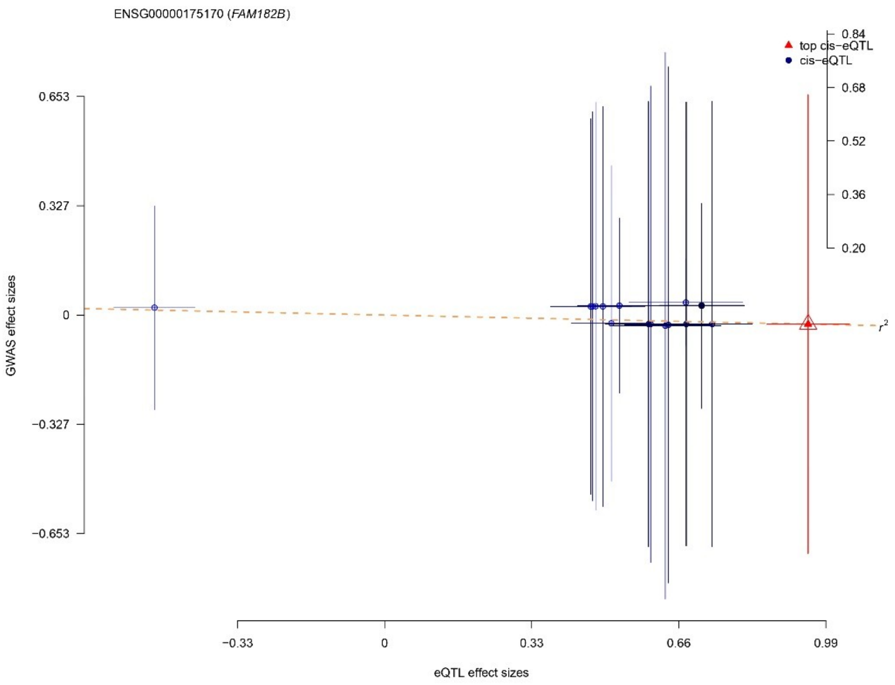

ENSG00000175170 (*FAM182B*)
top cis−eQTL
cis−eQTL
0.84
0.68
0.52
0.36
0.20
r^2
0.653
0.327
0
−0.327
−0.653
GWAS effect sizes
−0.33
0
0.33
0.66
0.99
eQTL effect sizes

J

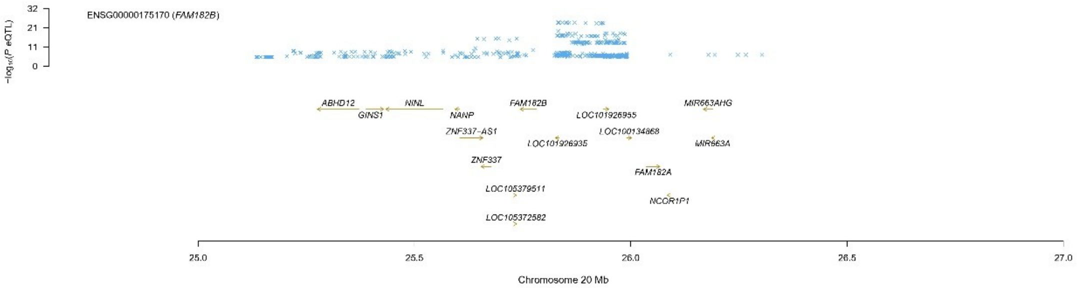

ENSG00000175170 (FAM182B)
−log10(P eQTL)
32
21
11
0
ABHD12
GINS1
NINL
NANP
FAM182B
LOC101926955
MIR663AHG
ZNF337-AS1
LOC101926935
LOC100134868
MIR663A
ZNF337
FAM182A
LOC105379511
NCOR1P1
LOC105372582
25.0
25.5
26.0
26.5
27.0
Chromosome 20 Mb

K

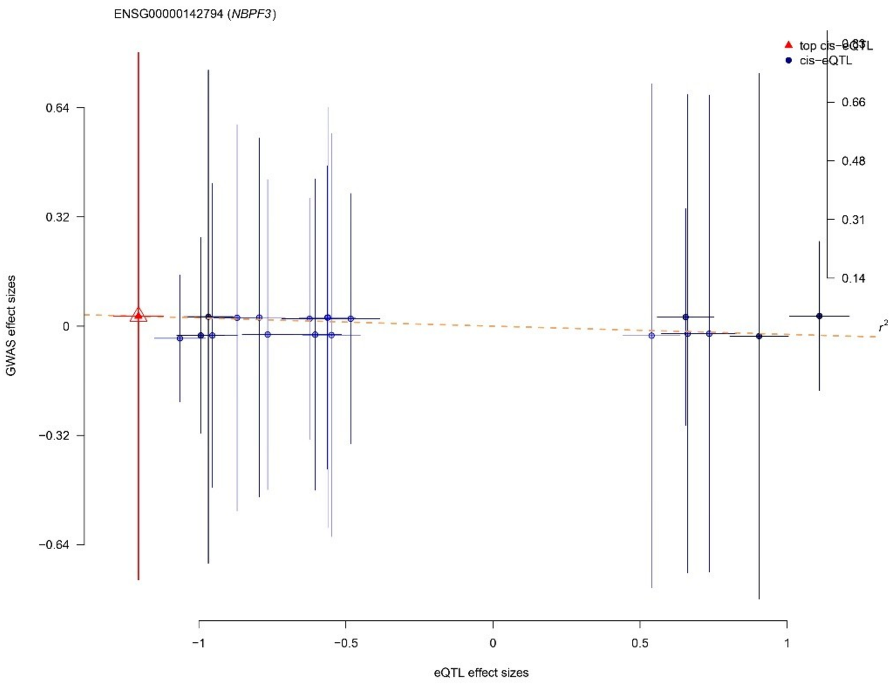

ENSG00000142794 (NBPF3)
top cis−eQTL
cis−eQTL
0.83
0.66
0.48
0.31
0.14
r^2
0.64
0.32
0
−0.32
−0.64
GWAS effect sizes
−1
−0.5
0
0.5
1
eQTL effect sizes

L

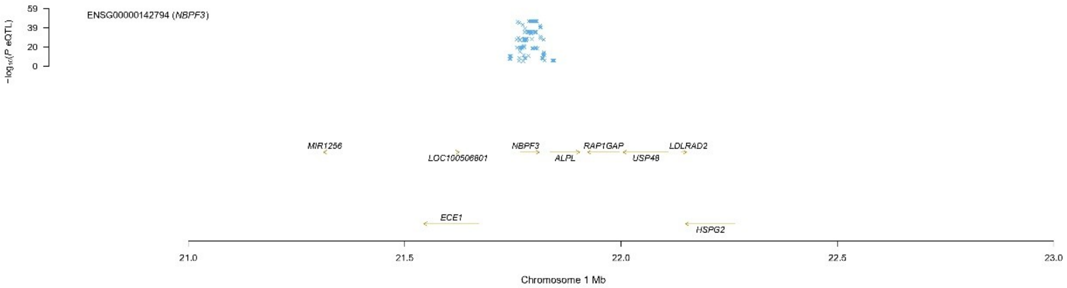

ENSG00000142794 (NBPF3)
59
39
20
0
−log10(P eQTL)
MIR1256
LOC100506801
NBPF3
ALPL
RAP1GAP
USP48
LDLRAD2
ECE1
HSPG2
21.0
21.5
22.0
22.5
23.0
Chromosome 1 Mb

M

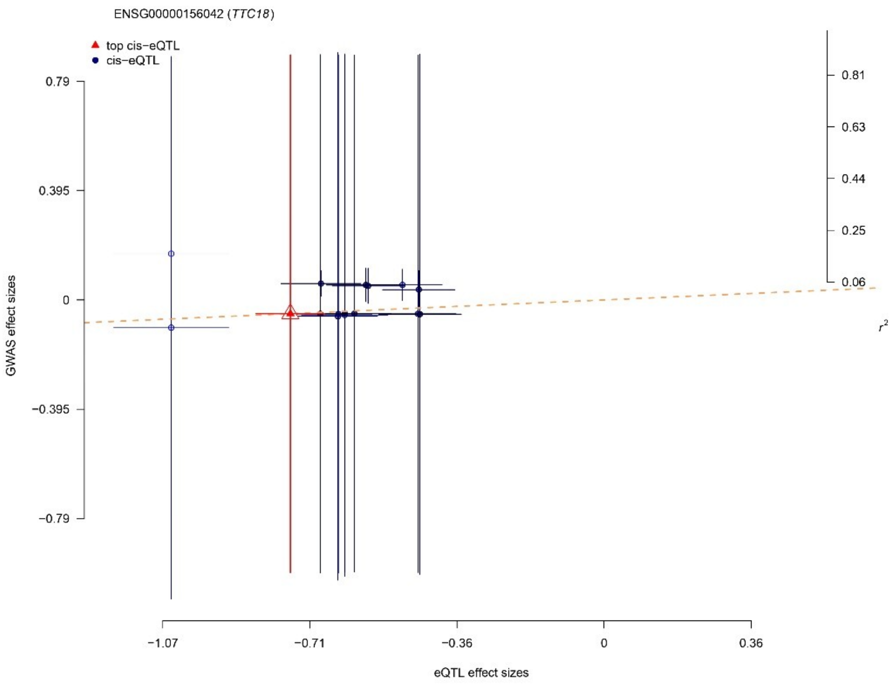

ENSG00000156042 (*TTC18*)
top cis−eQTL
cis−eQTL
0.79
0.395
0
−0.395
−0.79
GWAS effect sizes
0.81
0.63
0.44
0.25
0.06
r^2
−1.07
−0.71
−0.36
0
0.36
eQTL effect sizes

N

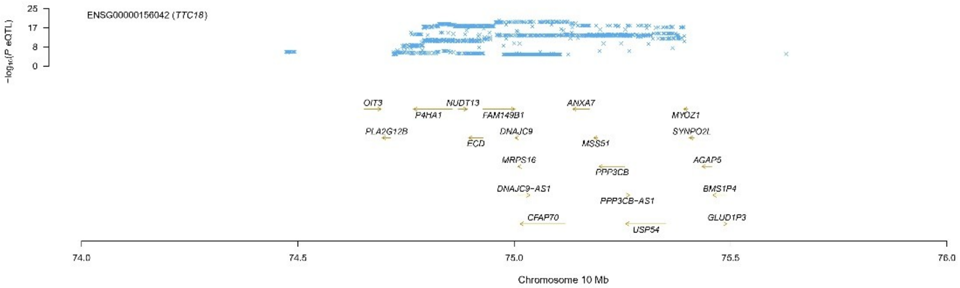

ENSG00000156042 (TTC18)
−log10(P eQTL)
25
17
8
0
OIT3
PLA2G12B
P4HA1
NUDT13
FCD
FAM149B1
DNAJC9
MRPS16
DNAJC9−AS1
CFAP70
ANXA7
MSS51
PPP3CB
PPP3CB−AS1
USP54
MYOZ1
SYNPO2L
AGAP5
BMS1P4
GLUD1P3
74.0
74.5
75.0
75.5
76.0
Chromosome 10 Mb

O

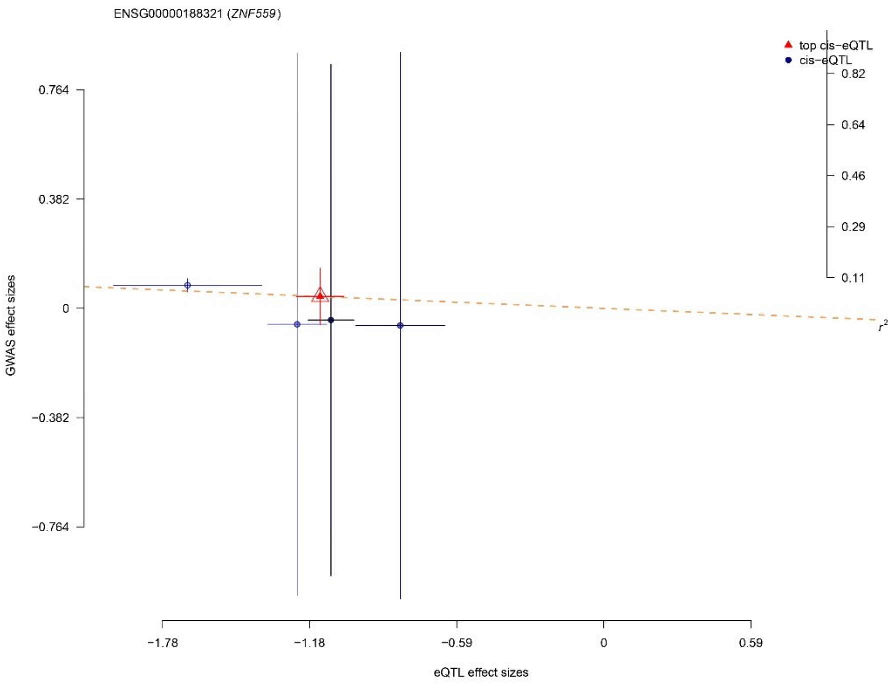
ENSG00000188321 (*ZNF559*)
top cis−eQTL
cis−eQTL
0.82
0.64
0.46
0.29
0.11
r^2
0.764
0.382
0
−0.382
−0.764
GWAS effect sizes
−1.78
−1.18
−0.59
0
0.59
eQTL effect sizes

P

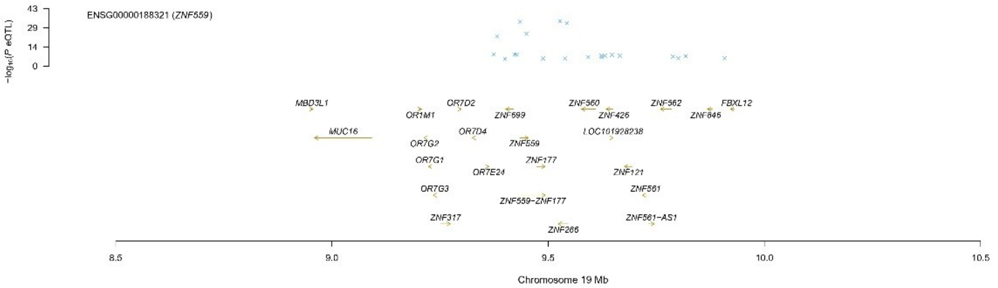

Figure S2. Colocalization plots for blood eQTLs and immune cell traits at loci of prioritized genes. For each gene–immune cell pair, the left panel displays the –log10(P) values for blood eQTLs (x-axis) versus immune cell eQTLs (y-axis), and the upper-right and lower-right panels show the regional association signals for blood eQTLs and immune cell traits, respectively, with colors indicating linkage disequilibrium (r^2) with the top variant. Panels A–I correspond to the following gene–immune cell pairs: A, BAIAP2L2_BAFF-R on IgD+ CD38dim cells; B, CLEC4D_CD27 on switched memory (sw mem) cells; C, FAM114A2 on transitional activated cells (Transitional AC); D, IGHV3-43_IgD on IgD+ CD38dim cells; E, IGHV3-49_IgD on IgD+ CD38dim cells; F, PLSCR1 on CD25hi CD45RA+ CD4 non-Treg activated cells (CD25hi CD45RA+ CD4 not Treg AC); G, RPP30 on CD39+ activated Treg cells (CD39+ activated Treg AC); H, RPP30 on CD39+ CD8bright activated cells (CD39+ CD8br AC); I, SEMA3C on CD39+ activated Treg cells (CD39+ activated Treg AC).

A

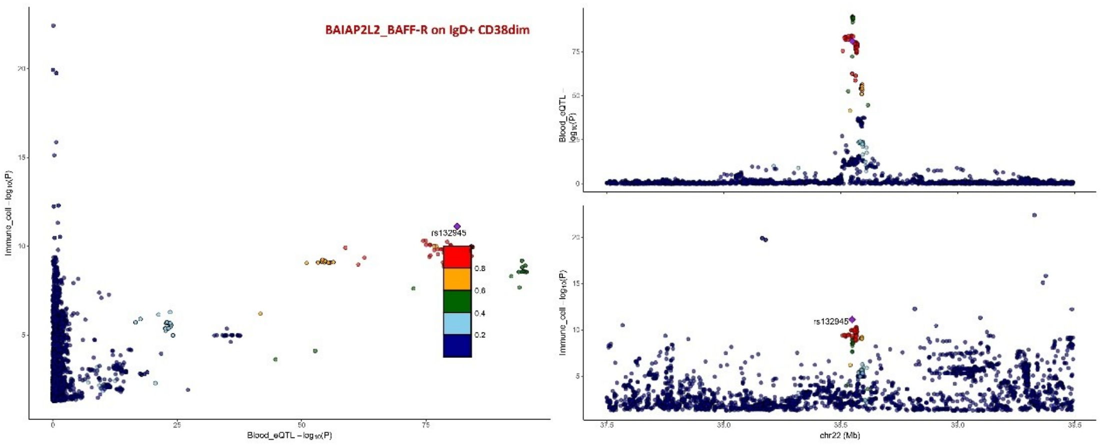
BAIAP2L2_BAFF-R on IgD+ CD38dim
rs132945
0.8
0.6
0.4
0.2
Blood_eQTL -log10(P)
Immune_cell -log10(P)
chr22 (Mb)

B

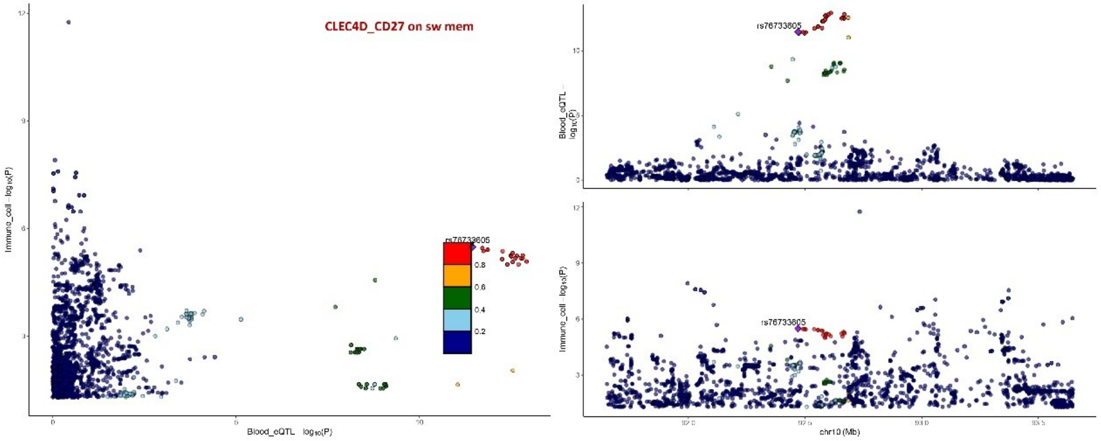
CLEC4D_CD27 on sw mem
rs76733605
0.8
0.6
0.4
0.2
Blood_eQTL -log10(P)
Immune_cell -log10(P)
chr10 (Mb)

C

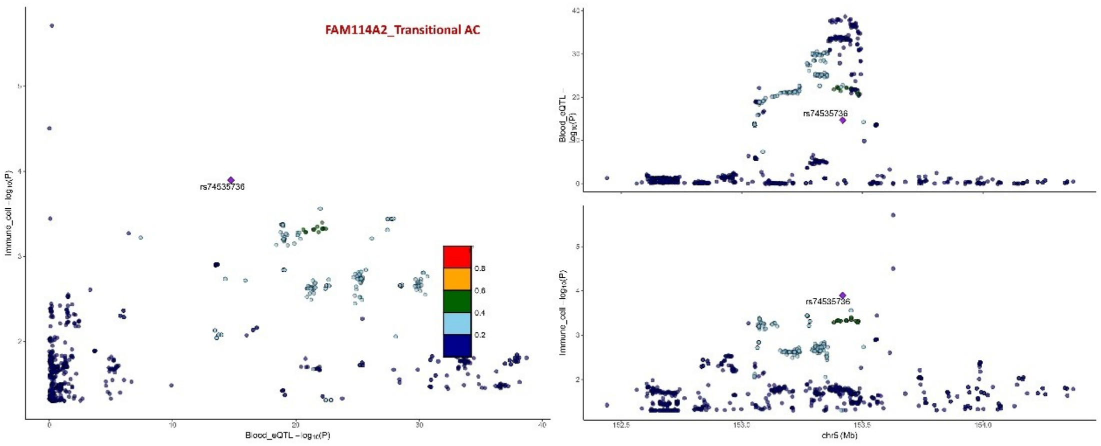

FAM114A2_Transitional AC
rs74535736
Immuno_cell -log10(P)
Blood_eQTL -log10(P)
0.8
0.6
0.4
0.2
rs74535736
rs74535736
chr5 (Mb)

D

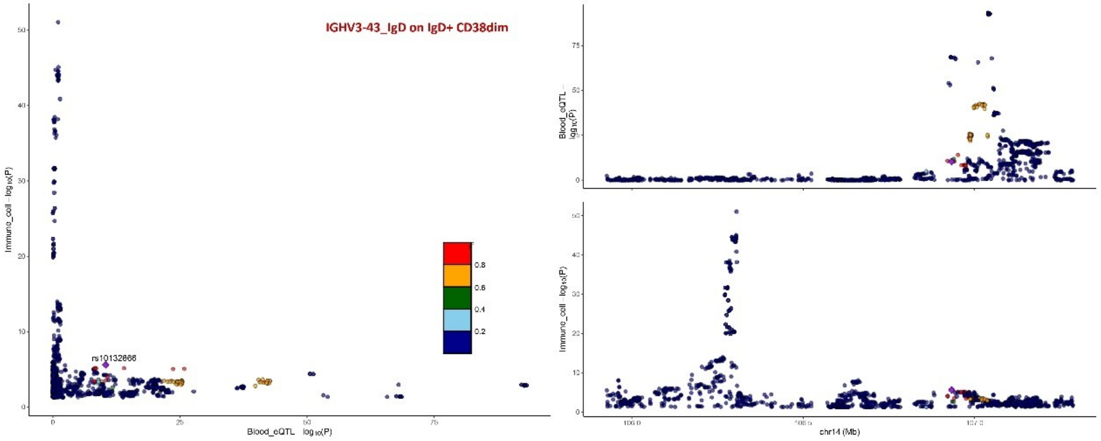

IGHV3-43_IgD on IgD+ CD38dim
rs10132866
Immuno_cell -log10(P)
Blood_eQTL -log10(P)
0.8
0.6
0.4
0.2
chr14 (Mb)

E

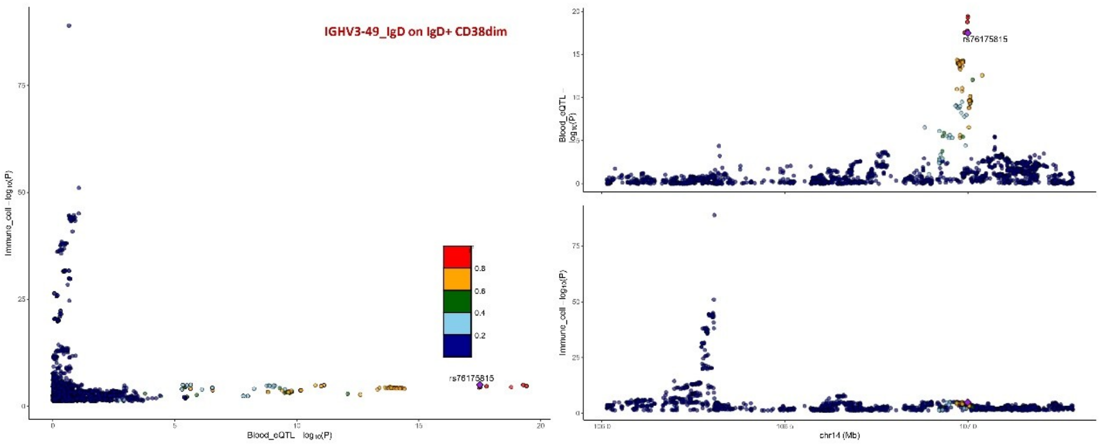

IGHV3-49_IgD on IgD+ CD38dim
rs76175815
Blood_eQTL -log10(P)
Immuno_cell -log10(P)
chr14 (Mb)
0.8
0.6
0.4
0.2

F

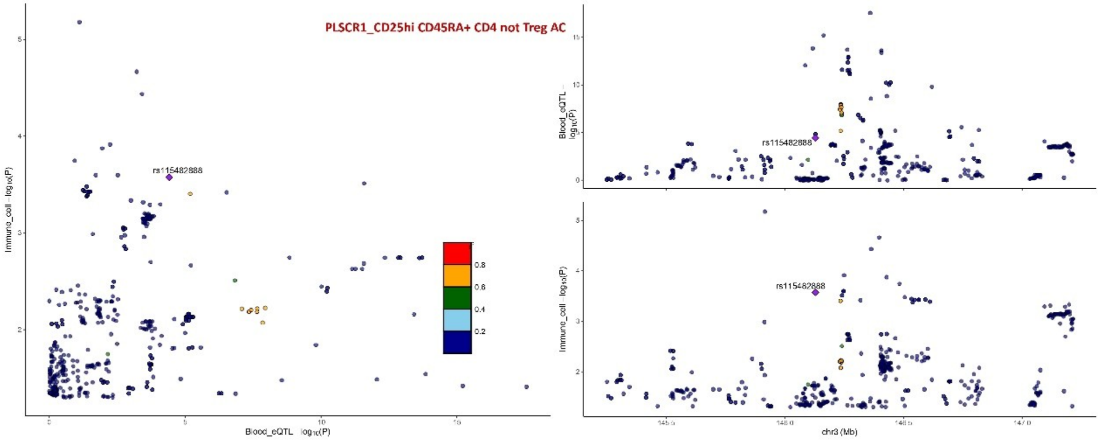

PLSCR1_CD25hi CD45RA+ CD4 not Treg AC
rs115482888
Blood_eQTL -log10(P)
Immuno_cell -log10(P)
chr3 (Mb)
0.8
0.6
0.4
0.2

G

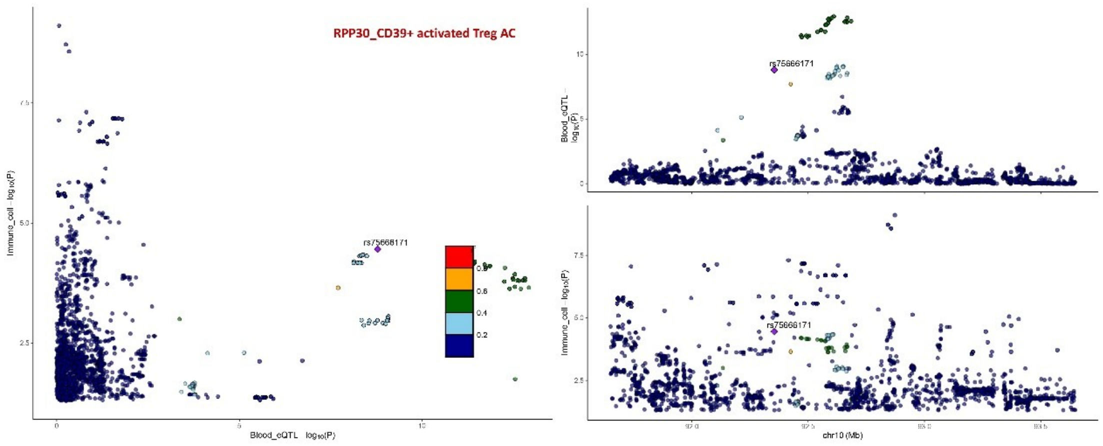

RPP30_CD39+ activated Treg AC
rs75666171
Immuno_cell -log10(P)
Blood_eQTL -log10(P)
chr10 (Mb)

H

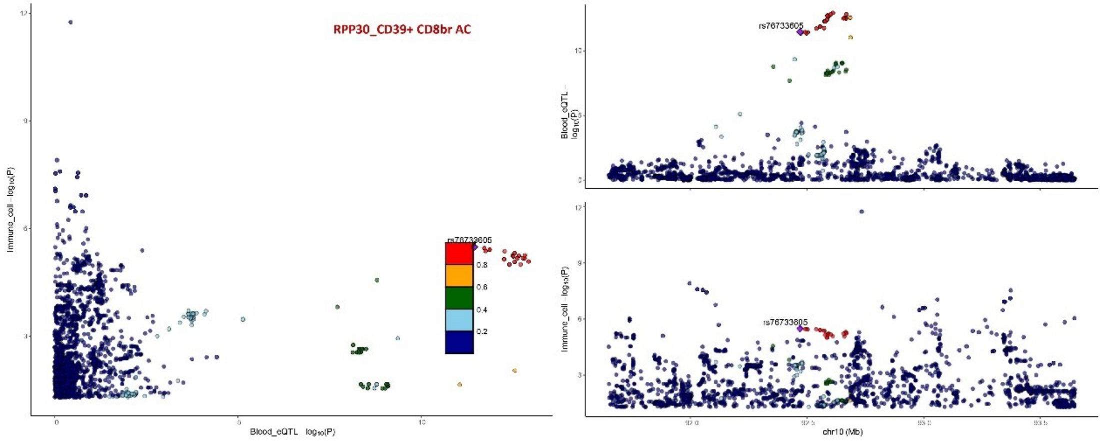

RPP30_CD39+ CD8br AC
rs76733605
0.8
0.6
0.4
0.2
Immuno_cell -log10(P)
Blood_eQTL -log10(P)
chr10 (Mb)

I

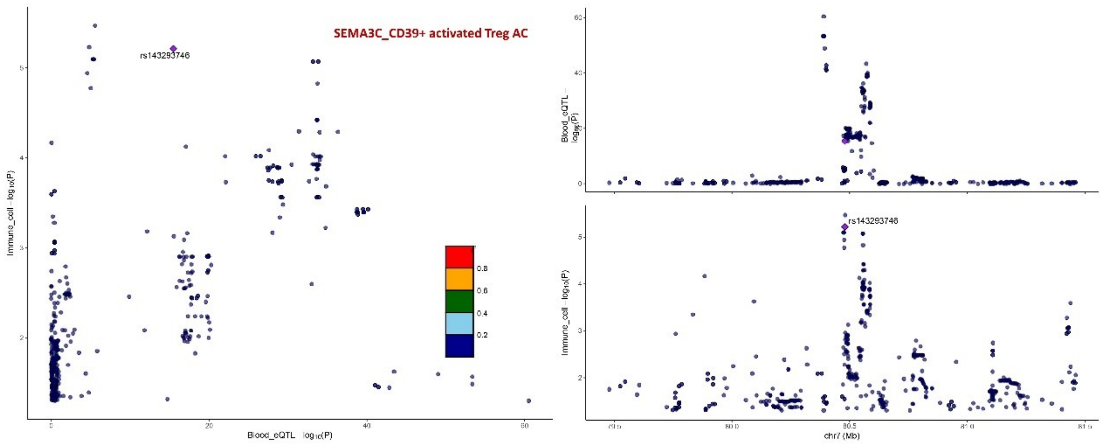
SEMA3C_CD39+ activated Treg AC
rs143293746
Immuno_cell -log10(P)
Blood_eQTL -log10(P)
0.8
0.6
0.4
0.2
Blood_eQTL -log10(P)
Immuno_cell -log10(P)
rs143293746
chr7 (Mb)
